# Time to Sustained Recovery Among Outpatients With COVID-19 Receiving Montelukast vs Placebo: The ACTIV-6 Randomized Clinical Trial

**DOI:** 10.1001/jamanetworkopen.2024.39332

**Published:** 2024-10-18

**Authors:** Russell L. Rothman, Thomas G. Stewart, Ahmad Mourad, David R. Boulware, Matthew W. McCarthy, Florence Thicklin, Idania T. Garcia del Sol, Jose Luis Garcia, Carolyn T. Bramante, Nirav S. Shah, Upinder Singh, John C. Williamson, Paulina A. Rebolledo, Prasanna Jagannathan, Tiffany Schwasinger-Schmidt, Adit A. Ginde, Mario Castro, Dushyantha Jayaweera, Mark Sulkowski, Nina Gentile, Kathleen McTigue, G. Michael Felker, Allison DeLong, Rhonda Wilder, Sean Collins, Sarah E. Dunsmore, Stacey J. Adam, George J. Hanna, Elizabeth Shenkman, Adrian F. Hernandez, Susanna Naggie, Christopher J. Lindsell

**Affiliations:** 1 Vanderbilt University Medical Center, Nashville, Tennessee; 2 School of Data Science, University of Virginia, Charlottesville; 3 Department of Medicine, Duke University School of Medicine, Durham, North Carolina; 4 Duke Clinical Research Institute, Duke University School of Medicine, Durham, North Carolina; 5 Department of Medicine, University of Minnesota Medical School, Minneapolis; 6 Weill Cornell Medicine, New York, New York; 7 ACTIV-6 Stakeholder Advisory Committee, Pittsburgh, Pennsylvania; 8 L&A Morales Healthcare, Inc, Hialeah, Florida; 9 The Angel Medical Research Corporation, Miami Lakes, Florida; 10 Endeavor Health, Evanston, Illinois; 11 Stanford University School of Medicine, California; 12 Section on Infectious Diseases, Department of Internal Medicine, Wake Forest University School of Medicine, Winston-Salem, North Carolina; 13 Division of Infectious Diseases, Department of Medicine, Emory University School of Medicine, Atlanta, Georgia; 14 Department of Global Health, Rollins School of Public Health, Emory University, Atlanta, Georgia; 15 Center for Clinical Research, University of Kansas School of Medicine–Wichita; 16 University of Colorado School of Medicine, Aurora; 17 Division of Pulmonary, Critical Care and Sleep Medicine, University of Kansas Medical Center, Kansas City; 18 Department of Medicine, Miller School of Medicine, University of Miami, Miami, Florida; 19 Division of Infectious Diseases, Johns Hopkins University, Baltimore, Maryland; 20 Department of Emergency Medicine, Lewis Katz School of Medicine at Temple University, Philadelphia, Pennsylvania; 21 Department of Medicine, University of Pittsburgh Medical Center, Pittsburgh, Pennsylvania; 22 Geriatric Research Education and Clinical Center, Veterans Affairs Tennessee Valley Healthcare System, Nashville; 23 National Center for Advancing Translational Sciences, Bethesda, Maryland; 24 Foundation for the National Institutes of Health, Bethesda, Maryland; 25 Biomedical Advanced Research and Development Authority, Washington, DC; 26 Department of Health Outcomes & Biomedical Informatics, College of Medicine, University of Florida, Gainesville

## Abstract

**Importance:**

The effect of montelukast in reducing symptom duration among outpatients with mild to moderate COVID-19 is uncertain.

**Objective:**

To assess the effectiveness of montelukast compared with placebo in treating outpatients with mild to moderate COVID-19.

**Design, Setting, and Participants:**

This randomized clinical trial (Accelerating COVID-19 Therapeutic Interventions and Vaccines [ACTIV]–6) was conducted from January 27 through June 23, 2023, during the circulation of Omicron subvariants. Participants aged 30 years or older with confirmed SARS-CoV-2 infection and 2 or more acute COVID-19 symptoms for less than 7 days were included across 104 US sites.

**Interventions:**

Participants were randomized 1:1 to receive montelukast, 10 mg once daily, or matched placebo for 14 days.

**Main Outcomes and Measures:**

The primary outcome was time to sustained recovery (defined as ≥3 consecutive days without symptoms). Secondary outcomes included time to death; time to hospitalization or death; a composite of health care utilization events (hospitalization, urgent care clinic visit, emergency department visit, or death); COVID-19 clinical progression scale score; and difference in mean time unwell. A modified intention-to-treat approach was used for the analysis.

**Results:**

Among 1250 participants who were randomized and received the study drug or placebo, the median age was 53 years (IQR, 42-62 years), 753 (60.2%) were female, and 704 (56.3%) reported receiving 2 or more doses of a SARS-CoV-2 vaccine. Among 628 participants who received montelukast and 622 who received placebo, differences in time to sustained recovery were not observed (adjusted hazard ratio [AHR], 1.02; 95% credible interval [CrI], 0.92-1.12; *P* = .63 for efficacy). Unadjusted median time to sustained recovery was 10 days (95% CI, 10-11 days) in both groups. No deaths occurred, and hospitalizations were reported for 2 participants (0.3%) in each group; the composite of health care utilization events was reported for 18 participants (2.9%) in the montelukast group and 18 (2.9%) in the placebo group (AHR, 1.01; 95% CrI, 0.45-1.84; *P* = .48 for efficacy). Five participants (0.4%) experienced serious adverse events (3 [0.5%] in the montelukast group and 2 [0.3%] in the placebo group).

**Conclusions and Relevance:**

In this randomized clinical trial of outpatients with mild to moderate COVID-19, treatment with montelukast did not reduce duration of COVID-19 symptoms. These findings do not support the use of montelukast for the treatment of mild to moderate COVID-19.

**Trial Registration:**

ClinicalTrials.gov Identifier: NCT04885530

## Key Points

**Question:**

Does a 14-day course of montelukast, 10 mg once daily, reduce symptom duration among outpatient adults (aged ≥30 years) with mild to moderate COVID-19 compared with placebo?

**Findings:**

In this randomized clinical trial of 1250 participants in the US (enrolled during the circulation of Omicron subvariants), there was no difference in time to sustained recovery between the montelukast and placebo groups.

**Meaning:**

Administration of montelukast at a daily dose of 10 mg for 14 days did not result in a shortened duration of symptoms in outpatient adults with mild to moderate COVID-19.

## Teaser

This randomized clinical trial compares the effectiveness of montelukast vs placebo to reduce time to recovery from mild to moderate symptoms of COVID-19 among US adult outpatients.

## Introduction

Recent clinical trials have evaluated novel and repurposed oral therapies for outpatients with mild to moderate COVID-19, without evidence of improved time to symptom recovery or clinical events.^1,2,3^ Montelukast, an orally active leukotriene receptor antagonist with anti-inflammatory effects, has been shown to suppress oxidative stress and cytokine production.^4^ While montelukast is currently approved for the treatment of asthma and allergic rhinitis, in silico screening (based on in vitro studies for other RNA viruses) supports the plausibility of antiviral activity through inhibition of SARS-CoV-2 protease and polymerase enzymes.^5^ Montelukast may also ameliorate extrapulmonary manifestations of COVID-19 either directly through blocking of cysteinyl leukotriene receptors or indirectly through inhibition of the NF-κB signaling pathway.^6^ Three prior clinical studies of hospitalized patients with COVID-19 suggested a potential benefit of montelukast for improving symptoms.^7,8,9^ However, these studies were small and had significant design limitations. To our knowledge, no clinical trials have assessed the potential role of montelukast in outpatients with mild to moderate COVID-19.

The ongoing Accelerating Coronavirus Disease 2019 Therapeutic Interventions and Vaccines (ACTIV-6) platform randomized clinical trial evaluates repurposed medications in the outpatient setting.^10^ For this study, the ACTIV-6 platform evaluated the effect of montelukast on time to sustained recovery in nonhospitalized adults with mild to moderate COVID-19.

## Methods

### Trial Design and Oversight

The design and rationale for ACTIV-6 has been previously published.^11^ ACTIV-6 (NCT04885530) is a double-blind, placebo-controlled platform randomized clinical trial evaluating repurposed medications for the treatment of outpatients with mild to moderate COVID-19 in the US.^12^ Using a hybrid decentralized approach that allows virtual enrollment as well as enrollment through diverse health care and community settings, ACTIV-6 has achieved broad reach. The complete protocol and statistical analysis plan are provided in Supplement 1. The trial protocol was approved by a central institutional review board (WCG IRB), with review at each site. Each study participant provided electronic informed consent. An independent data and safety monitoring committee oversaw participant safety, efficacy, and trial conduct. Reporting followed the Consolidated Standards of Reporting Trials (CONSORT) guideline.^13^

### Participants

The montelukast arm was open for enrollment from January 27 through June 23, 2023, during which 104 sites were open. The Omicron subvariants were circulating during this time. Participants were identified by enrolling sites or by self-referral through the central study call center.

Study eligibility criteria included age of 30 years or older, SARS-CoV-2 infection within the past 10 days, and actively experiencing 2 or more COVID-19 symptoms for fewer than 7 days from the time of consent (full eligibility criteria are in Supplement 1). Participants were required to provide documentation of SARS-CoV-2 infection, which could include a picture (with date) of a home antigen or polymerase chain reaction (PCR) test or a picture, screenshot, or document of a test that was conducted at a clinic site (including antigen or PCR tests). Patient characteristics and outcomes were collected by self-report via direct REDCap or telephone survey. Race and ethnicity categories from the survey were American Indian or Alaska Native; Asian; Black, African American, or African; Middle Eastern or North African; Native Hawaiian or Other Pacific Islander; White; Hispanic or Latino; and not Hispanic or Latino. Participants could select any combination of these descriptors, “none of the above,” or “prefer not to answer.” These data were collected to assess generalizability based on representativeness of the study population due to the disparities of rates of infection and severe outcomes across these populations.^14^ Individuals were excluded from participation if they had current or recent hospitalization for COVID-19; ongoing or planned participation in other interventional trials for COVID-19; or current or recent use of, known allergy or sensitivity to, or contraindication to montelukast. Receipt of COVID-19 vaccinations or current use of approved or emergency use authorization therapeutics for outpatient treatment of COVID-19 were allowed.

### Randomization

The period of enrollment for montelukast did not overlap with the enrollment period of other active drugs in the adaptive ACTIV-6 platform. Consequently, the randomization process simplified to a 1:1 matched placebo allocation provided by a random number generator with no pooled placebo contribution.

### Interventions

A 14-day supply of either montelukast or matched placebo was dispensed to each participant via home delivery from a centralized pharmacy. Participants were instructed to self-administer oral montelukast at a dose of one 10-mg tablet or matching placebo daily for 14 days.

### Outcome Measures

The primary outcome was time to sustained recovery within 28 days, defined as the time from receipt of drug to the third of 3 consecutive days without COVID-19 symptoms.^10,12^ Participants who died within the follow-up period were deemed to have not recovered. Secondary outcomes included 3 time-to-event end points administratively censored at day 28: time to death, time to hospitalization or death, or time to first health care utilization (a composite of urgent care clinic visits, emergency department visits, hospitalization, or death). Additional secondary outcomes included mean time spent unwell through day 14 and the World Health Organization COVID-19 Clinical Progression Scale on days 7, 14, and 28. Quality-of-life measures using the PROMIS-29 questionnaire^15^ were being collected through day 180 and are not included in this report.

### Trial Procedures

The ACTIV-6 platform was designed to be conducted remotely, with all screening and eligibility procedures reported by participants and confirmed at the site level. Positive laboratory results for SARS-CoV-2 were verified by study staff prior to randomization. Participants self-reported demographic information, medical history, use of concomitant medications, and COVID-19 symptoms and completed quality-of-life surveys.

A centralized investigational pharmacy packaged and provided active or placebo study products via courier to participants. On February 23, 2023, the ACTIV-6 study team was notified of a voluntary recall of a batch of montelukast by the manufacturer (Intas Pharmaceuticals Ltd). Although the recall was voluntary, with an abundance of caution, enrollment was paused and distribution of the study drug and placebo ceased. By February 27, 2023, a replacement product had been sourced that matched the original in appearance apart from debossing. Details about drug appearance were removed from the protocol to minimize risk of unblinding, and the study arm was reopened on March 3, 2023. Notification of the recall was sent to the 149 participants who were either currently taking the study drug or placebo or to whom the drug was in the process of being shipped and who would have been eligible for inclusion in the modified intention-to-treat (MITT) analysis cohort. While these participants were told that their study medication was considered safe, adherence was expected to be influenced by the communication; thus, the ACTIV-6 investigators decided a priori to exclude all notified participants from the MITT assessment of the primary and secondary outcomes. However, they would be included in any analyses that adjusted for adherence to the study drug. The target recruitment was increased to achieve a minimum of 1200 participants in the MITT analysis set.

Daily assessments and adverse events were reported by participants via the study portal during the first 14 days of the study regardless of symptom status. If participants had not recovered by day 14, the daily assessments continued until sustained recovery or day 28. Planned remote follow-up visits occurred on days 28, 90, and 120. Additional study procedure details are provided in Supplement 1.

### Statistical Analysis

Cox proportional hazards regression was used for the time-to-event analysis, and cumulative probability ordinal regression models were used for ordinal outcomes. Longitudinal ordinal regression models were used to estimate the differences in mean time spent unwell.

The planned primary end point analysis was a bayesian proportional hazards model. The primary inferential decision-making quantity was the posterior distribution for the treatment-assignment hazard ratio (HR), with an HR greater than 1 indicating a beneficial effect. If the posterior probability of benefit exceeded 0.95 during interim or final analyses, intervention efficacy would be met. To preserve a type I error less than 0.05, the prior for the treatment effect parameter on the log relative-hazard scale was a normal distribution centered at 0 and scaled to an SD of 0.1. All other parameter priors were weakly informative, using the default of 2.5 times the ratio of the SD of the outcome divided by the SD of the variable. The study was designed to have 80% power to detect an HR of 1.2 in the primary end point from a total sample size of 1200 participants, with planned interim analyses at 300, 600, and 900 participants.

The model for the primary end point included the following variables: randomization assignment, age, sex, duration of symptoms prior to study drug or placebo receipt, calendar time, vaccination status, geographic location, call center indicator, and baseline symptom severity. The proportional hazards assumption of the primary end point was evaluated by generating visual diagnostics, such as log-log plots and plots of time-dependent regression coefficients, for each model variable.

Secondary end points were analyzed with bayesian regression models (either proportional hazards or proportional odds). Weakly informative priors were used for all parameters. Secondary end points were not used for formal decision-making, and no decision threshold was selected. With the exception of time unwell, the same covariates used in the primary end point model were used in the secondary end point analyses provided that the end point accrued sufficient events to be analyzed with covariate adjustment. For secondary end points, HR and odds ratio (OR) estimates less than 1 favored montelukast.

All available data were used to compare each active study drug with the placebo control regardless of postrandomization adherence. The MITT cohort comprised all participants who were randomized, who did not withdraw before delivery of the study drug or placebo, who were not notified of the drug recall, and for whom the courier confirmed drug delivery. Study day 1 was defined as the day of study drug or placebo delivery. Participants who opted to discontinue data collection were censored at the time of last contact, including those who did not complete any surveys or telephone calls after receipt of the study drug or placebo. Missing data among covariates for both primary and secondary analyses were addressed with conditional mean imputations.

A predefined analysis examined potential variations in treatment effects based on participant characteristics. The assessment of treatment effect heterogeneity encompassed age, symptom duration, body mass index (BMI; calculated as weight in kilograms divided by height in meters squared), symptom severity on day 1, calendar time (indicative of circulating SARS-CoV-2 variants), sex, and vaccination status. Continuous variables were analyzed as such without stratifying into subgroups. A priori, there was concern that the call center could enroll a different and larger population than sites, and an indicator for call center was specified in the model with the intent to assess heterogeneity of treatment effect by site should this occur (eMethods in Supplement 2). Post hoc, it was identified that 1 site had recruited 573 participants. As a sensitivity analysis, the primary end point model was expanded to include site-indicator variables from all sites, not just the call center. In an additional post hoc sensitivity analysis, the baseline hazard of recovery was stratified by site (with sites contributing <11 participants grouped together). In addition, the possibility of a differential treatment effect by site was assessed.

Analyses were performed with R, version 4.3 (R Project for Statistical Computing)^16^ with the following primary packages: rstanarm,^17,18^ rmsb,^19^ and survival.^20^ For the heterogeneity of treatment effect analysis, 2-sided *P* < .05 was considered significant.

## Results

### Study Population

**Figure 1.**
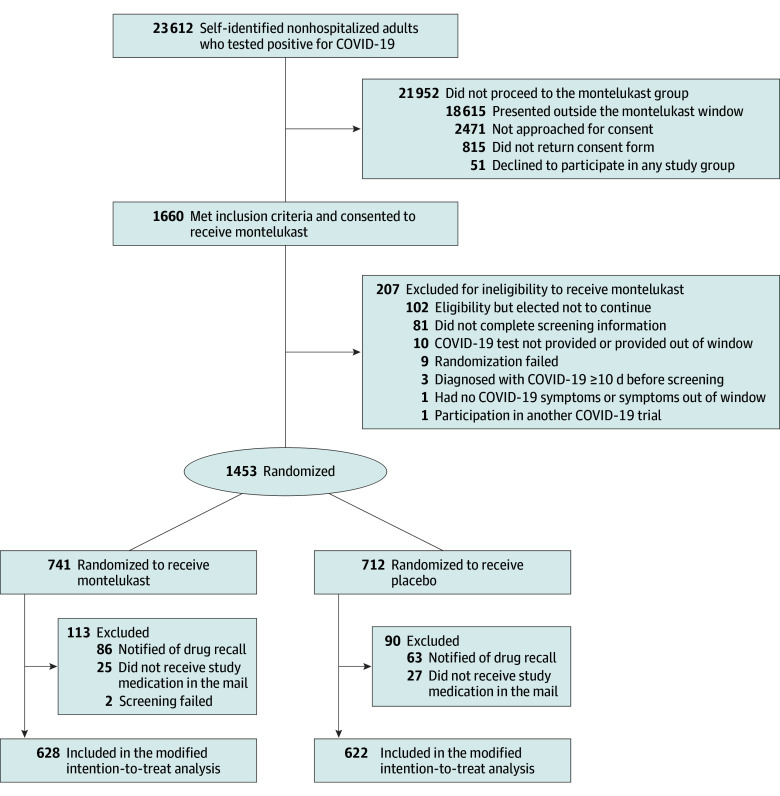
Participant Flow in a Trial of Montelukast for Mild to Moderate COVID-19 Randomization failures occurred when an individual was no longer eligible for inclusion at the time of randomization.

Of 1453 participants enrolled in the montelukast study arm, 1250 were included in the MITT cohort (participants who were randomized, received the study drug or placebo, did not withdraw from the study before receiving the study drug or placebo, and were not notified of the medication recall); 628 were assigned to receive montelukast and 622 to matched placebo (Figure 1). The median age was 53 years (IQR, 42-62 years); 753 participants (60.2%) were female, and 497 (39.8%) were male. Overall, 6 (0.5%) were American Indian or Alaska Native; 45 (3.6%), Asian; 160 (12.8%), Black, African American, or African; 19 (1.5%), Middle Eastern or North African; 3 (0.2%), Native Hawaiian or Other Pacific Islander; 978 (78.2%), White; 35 (2.8%), none of these races; and 13 (1.0%) preferred not to answer. A total of 807 participants (64.6%) identified as Hispanic or Latino ethnicity and 443 (35.4%) as not Hispanic or Latino. The most common comorbidities were obesity, assessed as BMI greater than 30 (567 of 1249 participants [45.4%]), and hypertension (277 of 1197 [23.1%]). Overall, 704 participants (56.3%) reported having received at least 2 doses of a SARS-CoV-2 vaccine and 153 (12.2%) reported taking a COVID-19 therapy (Table 1).

**Table 1.** Baseline Participant Characteristics

Variable	Participants^a^	
	Montelukast arm (n = 628)	Placebo arm (n = 622)
Age, median (IQR), y	52.0 (42.0-61.0)	54.0 (43.0-62.0)
Age <50 y	278 (44.27)	241 (38.75)
Sex^b^		
Female	388 (61.78)	365 (58.68)
Male	240 (38.22)	257 (41.32)
Race^c^		
American Indian or Alaska Native	3 (0.48)	3 (0.48)
Asian	23 (3.66)	22 (3.54)
Black, African American, or African	85 (13.54)	75 (12.06)
Middle Eastern or North African	8 (1.27)	11 (1.77)
Native Hawaiian or Other Pacific Islander	2 (0.32)	1 (0.16)
White	488 (77.71)	490 (78.78)
None of the above	16 (2.55)	19 (3.05)
Prefer not to answer	7 (1.11)	6 (0.96)
Ethnicity		
Hispanic or Latino	415 (66.08)	392 (63.02)
Not Hispanic or Latino	213 (33.92)	230 (36.98)
Region^d^		
Midwest	83 (13.22)	98 (15.76)
Northeast	30 (4.78)	22 (3.54)
South	491 (78.18)	465 (74.76)
West	24 (3.82)	37 (5.95)
Recruited via call center^e^	4 (0.64)	4 (0.64)
BMI		
Median (IQR)	29.7 (26.6-33.2)	29.3 (26.4-32.5)
>30, No./total No. (%)	291/627 (46.41)	276/622 (44.37)
Weight, median (IQR), kg	81.6 (74.8-92.1)	82.3 (72.6-92.0)
Medical history, No./total No. (%)^f^		
Hypertension	138/603 (22.89)	139/594 (23.40)
Diabetes	46/603 (7.63)	68/593 (11.47)
Smoker, past year	51/603 (8.46)	49/594 (8.25)
Asthma	50/601 (8.32)	48/593 (8.09)
Heart disease	14/601 (2.33)	24/593 (4.05)
Malignant cancer	14/597 (2.35)	10/588 (1.70)
COPD	10/603 (1.66)	6/593 (1.01)
Chronic kidney disease	4/602 (0.66)	6/593 (1.01)
SARS-CoV-2 vaccine status		
Not vaccinated	279 (44.43)	267 (42.93)
1 dose	0	0
≥2 doses	349 (55.57)	355 (57.07)
Time between symptom onset and receipt of drug, median (IQR), d	5 (4-6)	5 (4-6)^g^
Time between symptom onset and enrollment, median (IQR), d	4 (2-5)^h^	4 (2-5)^i^
Symptom burden on study day 1, No./total No. (%)^j^		
None	21/558 (3.76)	20/553 (3.62)
Mild	186/558 (33.33)	183/553 (33.09)
Moderate	341/558 (61.11)	337/553 (60.94)
Severe	10/558 (1.79)	13/553 (2.35)
COVID-19 medications		
Remdesivir	5 (0.80)	2 (0.32)
Nirmatrelvir plus ritonavir	64 (10.19)	64 (10.29)
Monoclonal antibodies	5 (0.80)	4 (0.64)
Molnupiravir	5 (0.80)	4 (0.64)

Abbreviations: BMI, body mass index (calculated as weight in kilograms divided by height in meters squared); COPD, chronic obstructive pulmonary disease.

^a^ Data are presented as number (percentage) of participants unless otherwise indicated.

^b^ Participants also had the option to select “unknown,” “undifferentiated,” or “prefer not to answer.” Only female and male were selected in this cohort.

^c^ Participants were presented with each of these options and may have selected any combination of the race descriptors, including “none of the above” and “prefer not to answer.” Consequently, the sums of counts over all categories do not match the column totals.

^d^ Northeast includes Connecticut, Maine, Massachusetts, New Hampshire, Rhode Island, Vermont, New Jersey, New York, and Pennsylvania; Midwest includes Indiana, Illinois, Michigan, Ohio, Wisconsin, Iowa, Kansas, Minnesota, Missouri, Nebraska, North Dakota, and South Dakota; South includes Delaware, District of Columbia, Florida, Georgia, Maryland, North Carolina, South Carolina, Virginia, West Virginia, Alabama, Kentucky, Mississippi, Tennessee, Arkansas, Louisiana, Oklahoma, and Texas; and West includes Arizona, Colorado, Idaho, New Mexico, Montana, Utah, Nevada, Wyoming, Alaska, California, Hawaii, Oregon, and Washington.

^e^ Patients may have alternatively been recruited at local clinical sites.

^f^ Medical history was provided by participants responding to the following prompts: “Has a doctor told you that you have any of the following?” “Have you ever experienced any of the following? (select all that apply),” and “Have you ever smoked tobacco products?”

^g^ Among 621 participants.

^h^ Among 619 participants.

^i^ Among 605 participants.

^j^ Each day, participants were asked, “Please choose the response that best describes the severity of your COVID-19 symptoms today.”

On study day 1, 41 of 1111 participants (3.7%) reported no symptoms while the majority reported mild (369 of 1111 [33.2%]) or moderate (678 of 1111 [61.0%]) symptoms. Baseline symptom burden for the 13 COVID-19–related symptoms is reported in eTable 1 in Supplement 2. Participants were enrolled within a median of 4 days (IQR, 2-5 days) of reported symptom onset, and the study drug or placebo was delivered within a median of 5 days (IQR, 4-6 days) from symptom onset; 1127 patients (90.2%) obtained their study drug within 7 days (eFigure 1 in Supplement 2).

### Primary Outcome

**Figure 2.**
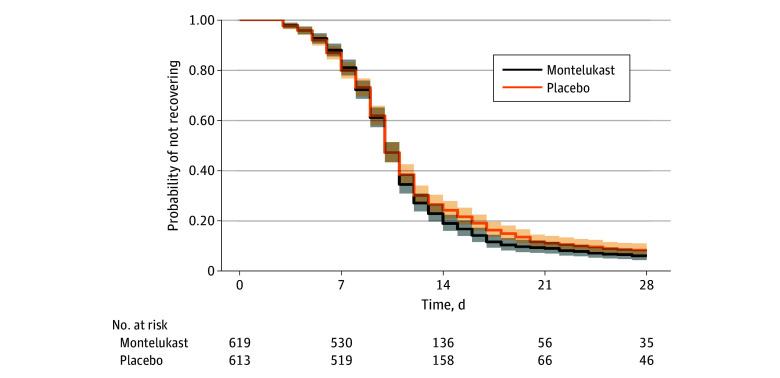
Primary Outcome of Time to Sustained Recovery Sustained recovery was defined as the third of 3 consecutive days without symptoms. Eighteen participants were censored for complete nonresponse and 28 after partial response; all others were followed up until recovery, death, or the end of short-term 28-day follow-up. Median time to sustained recovery was 10 days (95% CI, 10-11 days) in both groups. Shading denotes the pointwise 95% CIs.

**Figure 3.**
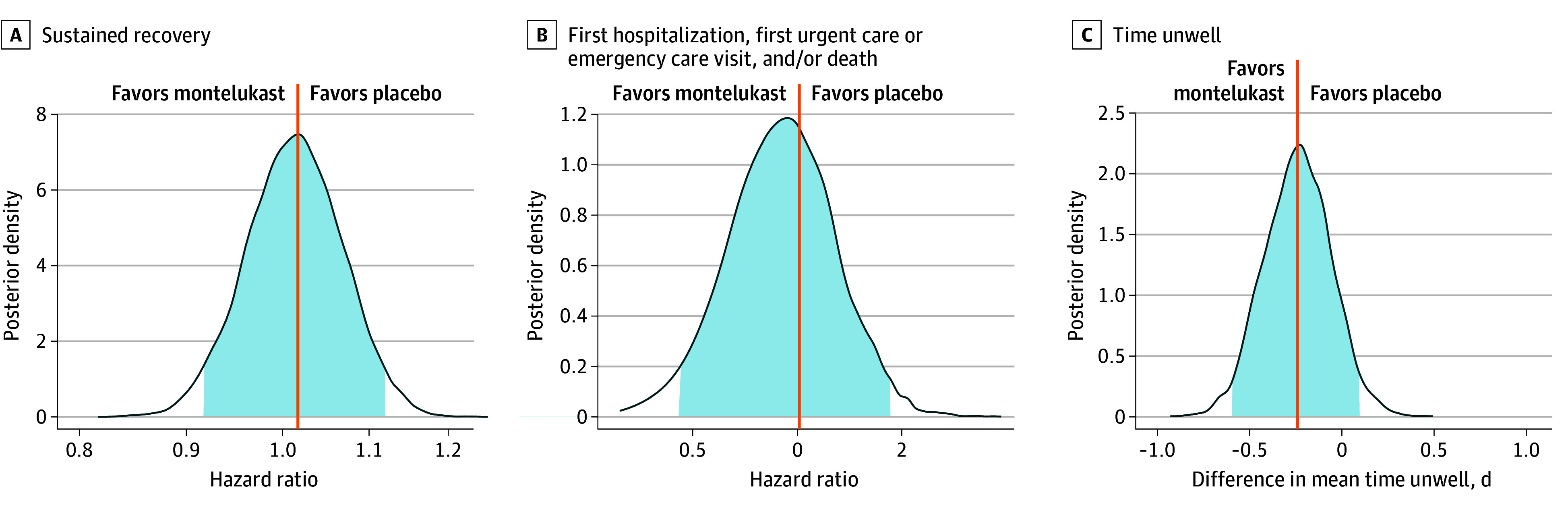
Posterior Distribution of Treatment Effect Hazard Ratio for Time to Outcomes Posterior density is the relative likelihood of posterior probability distribution. Outcomes with higher posterior density are more likely than outcomes with lower posterior density. Blue density lines represent kernel density estimates constructed from posterior draws; vertical lines, estimated means of the posterior distribution; shading, the 95% credible interval. Posterior density plots of all the covariates in the primary outcome model are shown in eFigure 10 in Supplement 2.

Differences in time to sustained recovery were not observed in either unadjusted Kaplan-Meier curves (Figure 2) or covariate-adjusted regression models (Table 2). The median time to sustained recovery was 10 days (95% CI, 10-11 days) in both the montelukast and placebo groups. The adjusted HR (AHR) was 1.02 (95% credible interval [CrI], 0.92-1.12; *P* = .63 for efficacy) (Figure 3). Sensitivity analyses yielded similar estimates of the treatment effect (eFigure 2 in Supplement 2).

**Table 2.** Primary and Secondary Outcomes

Outcome	Participants, No. (%)		Adjusted estimate HR (95% CrI)^a^	Posterior *P* value (efficacy)
	Montelukast arm (n = 628)	Placebo arm (n = 622)		
**Primary end point**				
Time to sustained recovery^b^				
Skeptical prior (primary analysis)	NA	NA	1.02 (0.91 to 1.12)	.63
Noninformative prior (sensitivity analysis)	NA	NA	1.02 (0.90 to 1.14)	.65
No prior (sensitivity analysis)	NA	NA	1.01 (0.90 to 1.14)^c^	NA
**Secondary end point** ^d^				
Hospitalization, urgent care, ED visit, or death through day 28^e^	18 (2.87)	18 (2.89)	1.01 (0.45 to 1.84)^c^	.48
Mortality at day 28	0	0	NA	NA
Hospitalization or death through day 28	2 (0.32)	2 (0.32)	1.00 (0.14 to 7.07)^c^	NA
**Secondary end point**	**Mean (95% CrI)**		**OR (95% CrI)**	**Posterior *P* value (efficacy)**
Clinical progression ordinal outcome scale^f^				
Day 7 (n = 1158)	NA	NA	1.31 (0.50 to 2.29)	.27
Day 14 (n = 1141)	NA	NA	0.71 (0.16 to 1.49)	.82
Day 28 (n = 1169)	NA	NA	1.48 (0.33 to 2.96)	.29
Time unwell, d^g^	11.77 (11.58-11.97)	12.01 (11.83-12.17)	NA^h^	.91

Abbreviations: CrI, credible interval; ED, emergency department; HR, hazard ratio; NA, not applicable; OR, odds ratio.

^a^ Unless otherwise noted, a highest-density credible interval is shown. Adjustment variables for time to sustained recovery, mortality, composite clinical end points, and clinical progression in addition to randomization assignment were age (as restricted cubic splines), sex, duration of symptoms prior to receipt of study drug, calendar time (as restricted cubic splines), vaccination status, geographic region (Northeast, Midwest, South, and West), call center indicator, and baseline symptom severity.

^b^ Time from receipt of study drug to achieving the third of 3 days of recovery. An HR greater than 1.0 is favorable for faster recovery for montelukast compared with placebo.

^c^ Low event rate precluded covariate adjustment. Maximum partial likelihood estimate (no prior) with a 95% CI.

^d^ For secondary outcomes, an HR or OR less than 1 favors montelukast.

^e^ A priori, death was a component of the composite outcome; however, no deaths were observed.

^f^ The COVID-19 clinical outcome scale ranges from 0 (no clinical or virologic evidence of infection) to 8 (death), with higher scores indicating more severe illness. See the eMethods in Supplement 3 for a full description.

^g^ Adjustment variables for mean time unwell in addition to randomization assignment included age and calendar time.

^h^ Difference in means was −0.24 (95% CI, −0.60 to 0.10), favoring montelukast.

### Secondary Outcomes

No deaths occurred in either group. Two participants (0.3%) in each study group were hospitalized (Table 2 and eFigure 3 in Supplement 2). There were 18 participants (2.9%) in the montelukast group and 18 (2.9%) in the placebo group with reported hospital admission, emergency department or urgent care visits, or death (Table 2 and eFigure 4 in Supplement 2). The AHR for the composite health care outcome was 1.01 (95% CrI, 0.45-1.84; *P* = .48 for efficacy) (Figure 3).

With clinical events like hospitalization and death being rare among participants, the COVID clinical progression scale (eMethods in Supplement 2) simplified to a self-reported evaluation of home activity levels (limited vs not) collected on study days 7, 14, and 28 (eFigure 5 in Supplement 2). By day 7, 564 (89.8%) of those receiving montelukast and 557 (89.6%) of those receiving placebo reported no limitations in activity, not meeting the prespecified thresholds for a beneficial treatment effect (OR, 1.31; 95% CrI, 0.50-2.29; *P* = .27 for efficacy). Likewise, the difference in mean time unwell was similar between the montelukast and placebo groups (11.8 days [95% CI, 11.6-12.0 days] vs 12.0 days [95% CI, 11.8-12.2 days]; difference, −0.24 days [95% CrI, −0.60 to 0.10 days]; *P* = .91 for >0 days of benefit; *P* < .001 for >1 day of benefit) (Figure 3).

### Adverse Events and Tolerability

Of the 628 participants assigned to montelukast, all but 28 (4.5%) reported taking their study medication at least once. Among the 622 assigned to placebo, 32 (5.1%) did not report taking their study medication at least once. Five participants (0.4%) experienced 1 serious adverse event, all of whom reported taking the study drug (eTable 2 in Supplement 2). The 3 events reported in the montelukast group (0.5% of participants) were pneumonia, lower-extremity cellulitis, and ovarian torsion. The 2 events reported in the placebo group (0.3% of participants) were pneumonia and acute appendicitis. A priori, neuropsychiatric adverse events were identified as being of special interest, but no such events were reported.

### Heterogeneity of Treatment Effect Analyses

Analyses of a priori–defined characteristics found that as time from symptom onset to receipt of study drug increased beyond 9 days, the treatment effect favored placebo (eFigure 6 in Supplement 2), but this represented only 28 study participants (2.2%). Similarly, the treatment effect in participants no longer reporting symptoms on study day 1 favored placebo, but this represented just 41 participants (3.3%) (eFigure 7 in Supplement 2). eFigures 8 and 9 in Supplement 2 show that the main results and treatment effect were not influenced by site. No other factors were associated with treatment effect.

## Discussion

In this randomized clinical trial of 1250 adults with mild to moderate COVID-19, montelukast, 10 mg daily for 14 days, did not improve time to sustained recovery compared with placebo. Several recent studies^7,8,9^ have suggested a possible benefit from montelukast for inpatients with COVID-19, but these studies all had significant design limitations. In an open-label clinical trial, 180 hospitalized patients with moderate to severe COVID-19 were randomized to 1 of 3 arms: gabapentin, gabapentin plus montelukast (10 mg daily), or dextromethorphan (control) for 5 days.^9^ The authors found that gabapentin plus montelukast reduced the frequency and severity of cough to a greater extent than gabapentin alone; however, the dextromethorphan group had better outcomes than either of the 2 experimental groups. Another study randomized 180 hospitalized patients with COVID-19 to receive standard of care alone or into 1 of 2 experimental groups: montelukast, 10 mg daily or 20 mg daily, for 5 days.^8^ The study found that levels of inflammatory markers were significantly lower at day 5 in the montelukast groups compared with group receiving standard of care alone; however, only the higher-dose montelukast group had improved pulmonary function testing. Too few clinical events of interest occurred to adequately assess differences between the groups. A third study, a retrospective study of 92 hospitalized patients, compared the COVID-19 ordinal scale scores of 30 patients receiving montelukast with those of 62 patients not receiving monteleukast.^7^ The authors reported significantly fewer clinical deterioration events at day 3 of hospitalization in participants receiving montelukast (10% vs 32%; *P* = .02). It is possible that these 3 studies found a potential benefit from montelukast for more severe COVID-19, while the ACTIV-6 trial did not find a benefit in patients with less severe disease.

### Limitations

Our study has 3 key limitations. First, due to the dynamic nature of the pandemic, including increased population-level immunity to COVID-19, evolving viral variants over time, and the characteristics of the enrolled population, there were few clinical events observed in this trial. This limited our ability to adequately evaluate the treatment effect of montelukast on relevant clinical outcomes. Second, a notable constraint of the decentralized trial approach is the necessity to send the study drug via courier, which does introduce a delay from time of enrollment to time of receipt of drug, as was evident by a difference in the median time from symptom onset to these 2 time points. This delay might be significant for a proposed antiviral mechanism of action. We achieved a median time from symptom onset to receiving study drug of 5 days (IQR, 4-6 days), with 1127 patients (90.2%) obtaining their study drug within a 7-day time frame. We were able to include time to receipt of study drug in the analysis of heterogeneity of treatment effect to understand effects of administration timing on the primary outcome. We excluded participants notified of the medication recall; however, the recall was unrelated to participant characteristics or patient outcomes, and the risk of imbalance from the recall was no larger than the risk of imbalance due to randomization. Finally, 573 enrollments (45.8%) occurred at 1 site. There was no evidence of a difference in treatment effect for this site compared with other sites, and we expect that the results generalize broadly to the US population.

## Conclusions

In this randomized clinical trial among outpatient adults with mild to moderate COVID-19, treatment with montelukast, 10 mg daily for 14 days, did not shorten time to sustained recovery compared with placebo. These findings do not support the use of montelukast for the treatment of mild to moderate COVID-19.

## Notes

Supplement 1.
Trial Protocol and Statistical Analysis Plan

Supplement 2.
eAppendix
eMethods **eTable 1.** Baseline Symptom Prevalence and Severity **eTable 2.** Serious Adverse Events **eFigure 1.** Time From Symptom Onset to Receipt of Drug **eFigure 2.** Sensitivity Analysis of Hazard Ratio for Time to Sustained Recovery **eFigure 3.** All-Cause Hospitalization or Death for Montelukast Vs Placebo **eFigure 4.** All-Cause Hospitalization, Urgent Care, Emergency Department Visit, or Death for Montelukast Vs Placebo **eFigure 5.** Participants’ COVID-19 Clinical Progression Scale at Day 7, 14, and 28 **eFigure 6.** Kaplan-Meier Curves of Sustained Recovery by Onset of Symptoms and Symptom Severity **eFigure 7.** Heterogeneity of Treatment Effect Between Montelukast and Placebo for Time to Recovery **eFigure 8.** Posterior Distribution of Treatment Effect Adjusted for Site **eFigure 9.** Heterogeneity of Treatment Effect by Site **eFigure 10.** Posterior Densities of Primary End Point Model

Supplement 3.
Accelerating COVID-19 Therapeutic Interventions and Vaccines (ACTIV)–6 Study Group and Investigators

Supplement 4.
Data Sharing Statement

## References

[1] Venkatesan P. Repurposing drugs for treatment of COVID-19. Lancet Respir Med. 2021;9(7):e63. doi:10.1016/S2213-2600(21)00270-8 34090608 PMC8175045

[2] Bramante CT, Huling JD, Tignanelli CJ, ; COVID-OUT Trial Team. Randomized trial of metformin, ivermectin, and fluvoxamine for COVID-19. N Engl J Med. 2022;387(7):599-610. doi:10.1056/NEJMoa2201662 36070710 PMC9945922

[3] Hammond J, Fountaine RJ, Yunis C, . Nirmatrelvir for vaccinated or unvaccinated adult outpatients with COVID-19. N Engl J Med. 2024;390(13):1186-1195. doi:10.1056/NEJMoa2309003 38598573 PMC11156287

[4] Sanghai N, Tranmer GK. Taming the cytokine storm: repurposing montelukast for the attenuation and prophylaxis of severe COVID-19 symptoms. Drug Discov Today. 2020;25(12):2076-2079. doi:10.1016/j.drudis.2020.09.013 32949526 PMC7493735

[5] Copertino DC, Duarte RRR, Powell TR, de Mulder Rougvie M, Nixon DF. Montelukast drug activity and potential against severe acute respiratory syndrome coronavirus 2 (SARS-CoV-2). J Med Virol. 2021;93(1):187-189. doi:10.1002/jmv.26299 32658304 PMC7405283

[6] Al-Kuraishy HM, Al-Gareeb AI, Almulaiky YQ, Cruz-Martins N, El-Saber Batiha G. Role of leukotriene pathway and montelukast in pulmonary and extrapulmonary manifestations of COVID-19: the enigmatic entity. Eur J Pharmacol. 2021;904:174196. doi:10.1016/j.ejphar.2021.174196 34004207 PMC8123523

[7] Khan AR, Misdary C, Yegya-Raman N, . Montelukast in hospitalized patients diagnosed with COVID-19. J Asthma. 2022;59(4):780-786. doi:10.1080/02770903.2021.1881967 33577360 PMC7938648

[8] Kerget B, Kerget F, Aydın M, Karaşahin Ö. Effect of montelukast therapy on clinical course, pulmonary function, and mortality in patients with COVID-19. J Med Virol. 2022;94(5):1950-1958. doi:10.1002/jmv.27552 34958142 PMC9015221

[9] Soltani R, Nasirharandi S, Khorvash F, Nasirian M, Dolatshahi K, Hakamifard A. The effectiveness of gabapentin and gabapentin/montelukast combination compared with dextromethorphan in the improvement of COVID-19- related cough: a randomized, controlled clinical trial. Clin Respir J. 2022;16(9):604-610. doi:10.1111/crj.13529 35908849 PMC9353294

[10] McCarthy MW, Naggie S, Boulware DR, ; Accelerating COVID-19 Therapeutic Interventions and Vaccines (ACTIV)-6 Study Group and Investigators. Effect of fluvoxamine vs placebo on time to sustained recovery in outpatients with mild to moderate COVID-19: a randomized clinical trial. JAMA. 2023;329(4):296-305. doi:10.1001/jama.2022.24100 36633838 PMC9857647

[11] Accelerating Covid-19 Therapeutic Interventions and Vaccines (ACTIV)-6 Study Group. ACTIV-6: operationalizing a decentralized, outpatient randomized platform trial to evaluate efficacy of repurposed medicines for COVID-19. J Clin Transl Sci. 2023;7(1):e221. doi:10.1017/cts.2023.644 38028354 PMC10643936

[12] Naggie S, Boulware DR, Lindsell CJ, ; Accelerating COVID-19 Therapeutic Interventions and Vaccines (ACTIV-6) Study Group and Investigators. Effect of ivermectin vs placebo on time to sustained recovery in outpatients with mild to moderate COVID-19: a randomized clinical trial. JAMA. 2022;328(16):1595-1603. doi:10.1001/jama.2022.18590 36269852 PMC9587497

[13] Schulz KF, Altman DG, Moher D; CONSORT Group. CONSORT 2010 Statement: updated guidelines for reporting parallel group randomised trials. Trials. 2010;11:32. doi:10.1186/1745-6215-11-32 20334632 PMC2857832

[14] Magesh S, John D, Li WT, . Disparities in COVID-19 outcomes by race, ethnicity, and socioeconomic status: a systematic-review and meta-analysis. JAMA Netw Open. 2021;4(11):e2134147. doi:10.1001/jamanetworkopen.2021.34147 34762110 PMC8586903

[15] Hays RD, Spritzer KL, Schalet BD, Cella D. PROMIS-29 v2.0 profile physical and mental health summary scores. Qual Life Res. 2018;27(7):1885-1891. doi:10.1007/s11136-018-1842-3 29569016 PMC5999556

[16] R Core Team. R: A Language and Environment for Statistical Computing. R Project for Statistical Computing; 2023.

[17] Goodrich B, Gabry J, Ali I, Brilleman S. (2023). rstanarm: bayesian applied regression modeling via Stan. *R package*, version 2.26.1. Accessed September 5, 2024. https://mc-stan.org/rstanarm/

[18] Brilleman SL, Elçi EM, Novik JB, . Bayesian survival analysis using the rstanarm R package. *arXiv*. Preprint posted online February 22, 2020. doi:10.48550/arXiv.2002.09633

[19] Harrell FE. Bayesian regression modeling strategies. R package rmsb, version 0.0.2. R Project for Statistical Computing; 2021.

[20] Therneau TM. Survival analysis. R package survival. R Project for Statistical Computing; 2023.

