# Supplementary material for: Time to Sustained Recovery Among Outpatients With COVID-19 Receiving Montelukast vs Placebo: The ACTIV-6 Randomized Clinical Trial
Source: JAMA Netw Open. 2024 Oct 18;7(10):e2439332. doi: 10.1001/jamanetworkopen.2024.39332 (PMC11581631; doi:10.1001/jamanetworkopen.2024.39332)
Supplement: Supplement 1. — Trial Protocol and Statistical Analysis Plan [file jamanetwopen-e2439332-s001.pdf]

ACTIV-6: COVID-19 Outpatient Randomized Trial to Evaluate Efficacy of Repurposed Medications

**IND Number:**	155481
**Principal Investigator:**	Adrian Hernandez, MD, MHS<br>Duke Clinical Research Institute<br>200 Morris St.<br>Durham, NC 27701<br>Phone: 919-668-7515<br>Email: adrian.hernandez@duke.edu
**Co-Principal Investigator/IND Sponsor:**	Susanna Naggie, MD, MHS<br>Duke Clinical Research Institute<br>200 Morris St.<br>Durham, NC 27701<br>Phone: 919-684-2584<br>Email: susanna.naggie@duke.edu
**Data Coordinating Center Investigator**	Chris Lindsell, PhD<br>Duke Clinical Research Institute<br>200 Morris St.<br>Durham, NC 27701<br>Phone: 919-668-8700<br>Email: chris.lindsell@duke.edu
**Lead Funding Agency:**	National Center for Advancing Translational Sciences
**Version:**	7.0
**Date:**	08DEC2022

Statement of Compliance

This trial will be conducted in compliance with the International Council for Harmonisation (ICH) E6 (R2) guideline for Good Clinical Practice (GCP), and the applicable regulatory requirements from the United States (US) Code of Federal Regulations (CFR), including 45 CFR 46 (Human Subjects Protection); 21 CFR 312 (Investigational New Drug); 21 CFR 50 (Informed Consent), and 21 CFR 56 (Institutional Review Board).

All individuals who are responsible for the conduct, management, or oversight of this study have completed Human Subjects Protection and ICH GCP Training.

Site Principal Investigator Statement

I have read the protocol, including all appendices, and the package insert(s)/product label(s), and I agree that the protocol contains all necessary details for my staff and me to conduct this study as described. I will personally oversee the conduct of this study as outlined herein and will make a reasonable effort to complete the study within the time designated. I agree to make all reasonable efforts to adhere to the attached protocol.

I will provide all study personnel under my supervision with copies of the protocol and access to all information provided by the sponsor or the sponsor's representative. I will discuss this material with study personnel to ensure that they are fully informed about the efficacy and safety parameters and the conduct of the study in general. I am aware that, before beginning this study, the IRB, or equivalent oversite entity must approve this protocol in the clinical facility where it will be conducted.

I agree to obtain informed consent from participants, as required by the IRB of record and according to government regulations and ICH guidelines. I further agree to report to the sponsor or its representative any adverse events in accordance with the terms of this protocol and the US CFR, Title 21, part 312.64, ICH GCP 4.11. I further agree to ensure the study is conducted in accordance with the provisions as stated and will comply with the prevailing local laws and customs.

____
Site Principal Investigator Name (Print)

____________________________________ _________________________
Site Principal Investigator Signature Date

Table of Contents

1. Protocol Summary 12
1.1. Synopsis 12
1.2. Schema 19

2. Introduction 21
2.1. Study Rationale 21
2.2. Background 21
2.3. Benefit/Risk Assessment 22
2.3.1. Risk Assessment 22
2.3.2. Benefit Assessment 22

3. Objectives and Endpoints 23

4. Study Design 25
4.1. Overall Design 25
4.2. End of Study Definition 26

5. Study Population 27
5.1. Inclusion Criteria 27
5.2. Exclusion Criteria 27
5.3. Recruitment and Engagement 27
5.3.1. Participant Recruitment 27
5.3.2. Participant Engagement 27
5.3.3. Participant Randomization Process 28
5.4. Screen Failures 28
5.5. Enrollment 29

6. Study Drug(s) 30
6.1. Repurposed Medication Treatments 30
6.2. Placebo 30
6.3. Study Drug Accountability 30
6.4. Concomitant Therapy 30
6.5. Intervention After the End of the Study 30

7. Participant Withdrawal/Termination and Study Termination 31
7.1. Participant Withdrawal/Termination 31
7.2. Premature Termination or Suspension of the Study 31
7.3. Lost to Follow-up 31

8. Study Assessments and Procedures 33
8.1. Schedule of Events 34
8.1.1. Screening 35
8.1.2. Intervention Period 35
8.1.3. Follow-up Period 36
8.1.4. Final Visit 37
8.2. Clinical Assessments 37
8.3. Quality of Life Questionnaires 38

9. Safety Assessments 40

9.1. Adverse Events and Serious Adverse Events 40
9.1.1. Adverse Device Effect (ADE) and Unanticipated Adverse Device Effect (UADE)....40
9.1.2. Collection Period for AE and SAE Information....41
9.1.3. Assessing Causality of a Serious Adverse Event....42
9.1.4. Reporting and Monitoring of SAEs 42
9.1.5. Events of Special Interest....43
9.2. Unanticipated Problem (UP) and Terminations....44
9.2.1. Definition of Unanticipated Problem....44
9.2.2. Reporting of an Unanticipated Problem 44

10. Statistical Considerations....45
10.1. Statistical Hypotheses....45
10.1.1. Primary Hypothesis....45
10.2. Sample Size Determination 45
10.3. Randomization....45
10.4. Blinding 46
10.5. Populations for Analyses 46
10.6. Statistical Analyses....46
10.6.1. General Considerations....47
10.6.2. Statistical modeling....47
10.6.3. Assessing Effectiveness (Primary Objective)....47
10.6.4. Interim Analyses (IA), Early Stopping, and Type-I Error Control....47
10.6.5. Sensitivity and Supplementary Analyses....49
10.6.6. Differential Treatment Effects and Subgroup Analyses 49
10.6.7. Secondary Clinical Endpoint 50
10.6.8. Exploratory Analysis 50
10.6.9. Adherence and Retention Analysis....50
10.7. Interim Reporting....50
10.8. Independent Data Monitoring Committee (IDMC) 51
10.9. Adjudication Committee....51

11. Ethical Standards....52
11.1. Institutional Review Board (IRB)....52
11.2. Informed Consent Process 52
11.3. Participant and Data Confidentiality....52
11.4. Site Management and Quality Assurance....53
11.5. Site Monitoring....53

12. Data Handling and Record Keeping....55
12.1. Data Collection and Management Responsibilities....55
12.2. Study Records Retention 55
12.3. Protocol Deviations....55
12.4. Publication and Data Sharing Policy 55

13. Study Leadership....57

14. Summary of Changes....58

15. References....65

16. Appendix A (Enrollment Closed 04FEB2022) – Ivermectin 400 71
16.1. Risk Assessment 71
16.2. Additional Appendix-Level Exclusion Criteria 72
16.2.1. Precautions 72
16.3. Ivermectin Information 73
16.3.1. Formulation, Appearance, Packaging, and Labeling 73
16.3.2. Drug Dispensing, Storage, and Stability 73
16.3.3. Dosing and Administration 73
16.3.4. Rationale for Selection of Dose 73
16.4. Placebo Information 75
16.4.1. Formulation, Appearance, Packaging, and Labeling 75
16.4.2. Drug Dispensing, Storage, and Stability 75
16.4.3. Dosing and Administration 75
16.5. Events of Special Interest 75
17. Appendix B (Enrollment Closed 27May2022) – Fluvoxamine Maleate 76
17.1. Risk Assessment 76
17.2. Additional Appendix-Level Exclusion Criteria 77
17.2.1. Precautions 78
17.3. Fluvoxamine Information 78
17.3.1. Formulation, Appearance, Packaging, and Labeling 78
17.3.2. Drug Dispensing, Storage, and Stability 79
17.3.3. Dosing and Administration 79
17.3.4. Rationale for Selection of Dose 79
17.4. Placebo Information 82
17.4.1. Formulation, Appearance, Packaging, and Labeling 82
17.4.2. Drug Dispensing, Storage, and Stability 82
17.4.3. Dosing and Administration 82
17.5. Events of Special Interest 82
18. Appendix C (Enrollment Closed 08FEB2022) – Fluticasone Furoate 83
18.1. Risk Assessment 83
18.2. Additional Appendix-Level Exclusion Criteria 84
18.2.1. Precautions 84
18.3. Fluticasone Furoate Information 84
18.3.1. Formulation, Appearance, Packaging, and Labeling 85
18.3.2. Drug Dispensing, Storage, and Stability 85
18.3.3. Dosing and Administration 85
18.3.4. Rationale for Selection of Dose 85
18.4. Placebo Information 86
18.4.1. Formulation, Appearance, Packaging, and Labeling 86
18.4.2. Drug Dispensing, Storage, and Stability 87
18.4.3. Dosing and Administration 87
18.5. Events of Special Interest 87

18.6. Safety Reporting for Fluticasone Furoate 87

19. Appendix D – Ivermectin 600 88
19.1. Risk Assessment 88
19.2. Additional Appendix-Level Exclusion Criteria 88
19.2.1. Precautions 88
19.3. Ivermectin Information 88
19.3.1. Formulation, Appearance, Packaging, and Labeling 88
19.3.2. Drug Dispensing, Storage, and Stability 88
19.3.3. Dosing and Administration 88
19.3.4. Rationale for Selection of Dose 89
19.4. Placebo Information 89
19.4.1. Formulation, Appearance, Packaging, and Labeling 89
19.4.2. Drug Dispensing, Storage, and Stability 89
19.4.3. Dosing and Administration 89
19.5. Events of Special Interest 89

20. Appendix E – Fluvoxamine Maleate 100 90
20.1. Risk Assessment 90
20.2. Additional Appendix-Level Exclusion Criteria 90
20.2.1. Precautions 90
20.3. Fluvoxamine Information 90
20.3.1. Formulation, Appearance, Packaging, and Labeling 90
20.3.2. Drug Dispensing, Storage, and Stability 90
20.3.3. Dosing and Administration 90
20.3.4. Rationale for Selection of Dose 90
20.4. Placebo Information 91
20.4.1. Formulation, Appearance, Packaging, and Labeling 91
20.4.2. Drug Dispensing, Storage, and Stability 91
20.4.3. Dosing and Administration 91
20.5. Events of Special Interest 92

21. Appendix F – Montelukast 93
21.1. Risk Assessment 93
21.2. Additional Appendix-Level Exclusion Criteria 94
21.2.1. Precautions 94
21.3. Study Drug Information 94
21.3.1. Formulation, Appearance, Packaging, and Labeling 94
21.3.2. Drug Dispensing, Storage, and Stability 94
21.3.3. Dosing and Administration 94
21.3.4. Rationale for Selection of Dose 94
21.4. Placebo Information 97
21.4.1. Formulation, Appearance, Packaging, and Labeling 97
21.4.2. Drug Dispensing, Storage, and Stability 97
21.4.3. Dosing and Administration 97
21.5. Events of Special Interest 97

Abbreviations

ACE	Angiotensin-converting Enzyme
ADE	Adverse Device Effect
AE	Adverse Event
ARB	Angiotensin II Receptor Blockers
ARNI	Angiotensin Receptor Neprilysin Inhibitor
BiPAP	Bilevel Positive Airway Pressure
BMD	Bone Mineral Density
BMI	Body Mass Index
CCC	Clinical Coordinating Center
CEA	Clinical Event Ascertainment
CFR	Code of Federal Regulations
CNS	Central Nervous System
CONSORT	Consolidated Standards of Reporting Trials
COPD	Chronic Obstructive Pulmonary Disease
cOR	Common Odds Ratio
COVID-19	Coronavirus Disease 2019
CPAP	Continuous Positive Airway Pressure
C-SSRS	Columbia-Suicide Severity Rating Scale
DCC	Data Coordinating Center
DIC	Disseminated Intravascular Coagulation
DUA	Data Use Agreement
ECMO	Extracorporeal Membrane Oxygenation
EDC	Electronic Data Capture
ESI	Event of Special Interest
FDA	Food and Drug Administration
GCP	Good Clinical Practice
HIPAA	Health Insurance Portability and Accountability Act
HIV	Human Immunodeficiency Virus
IA	Interim Analysis
IC_{50}	Half-Maximal Inhibitory Concentration
ICF	Informed Consent Form
ICH	International Council for Harmonisation
ICMJE	International Committee of Medical Journal Editors
ICS	Inhaled Corticosteroid
ICU	Intensive Care Unit
IDMC	
IDS	Investigational Drug Service
IRB	Institutional Review Board
KO	Knockout
LPS	Lipopolysaccharide
MAOI	Monoamine Oxidase Inhibitors
mITT	Modified Intention to Treat
MOP	Manual of Procedures
NCATS	National Center for Advancing Translational Sciences
NIH	National Institute of Health

OCD	Obsessive Compulsive Disorder
OHRP	Office for Human Research Protections
OR	Odds Ratio
PASC	Post-Acute Sequelae of SARS-CoV-2 Infection
PCORI	Patient-Centered Outcomes Research Institute
PCR	Polymerase Chain Reaction
PHI	Personal Health Information
PHQ	Patient Health Questionnaire
PI	Principal Investigator
PPOS	Predicted Probability of Success
PROMIS	Patient-reported Outcomes Measurement Information System
QOL	Quality of Life
RNA	Ribonucleic Acid
RRT	Renal Replacement Therapy
SAE	Serious Adverse Event
SAP	Statistical Analysis Plan
SARS-CoV-1/2	Severe Acute Respiratory Syndrome Coronavirus 1/2
SD	Standard Deviation
sIA	Screening Interim Analysis
SNRI	Selective Norepinephrine Reuptake Inhibitor
SSRI	Selective Serotonin Norepinephrine Reuptake Inhibitor
SUSAR	Serious Unexpected Suspected Adverse Reaction
UADE	Unanticipated Adverse Device Effect
UP	Unanticipated Problems
US	United States
WT	Wildtype

Table of Figures

Figure 1: ACTIV-6 Study Schema 19

Figure 2. ACTIV-6 Interim Analysis Schema 20

Figure 3: Operational Structure Diagram 57

Figure 4: Survival curve of WT and S1R KO mice 80

Figure 5: Summary of JAMA Randomization Clinical Trial of Fluvoxamine for Early COVID-19 81

Figure 6: Summary of study results for the prospective, nonrandomized observational cohort study with fluvoxamine in participants diagnosed with COVID-19. 81

Figure 7. Probability of efficacy for the primary outcome in the ITT and mITT populations of TOGETHER 91

List of Tables

Table 1: Schedule of Events 34

Table 2: ACTIV-6 Sample Size Estimates and Power 45

Table 3: Ivermectin Adverse Event Table for Doses ≥ 300 µg/kg 71

Table 4: Ivermectin 400 Dosing Schedule 73

Table 5. Fluvoxamine Adverse events occurring in 10-week studies of adult OCD or depression 76

Table 6. Fluvoxamine Adverse events that occurred in COVID-19 study participants receiving 300 mg/day for 15 days 77

Table 7. Fluticasone Adverse reactions that occurred in ≥3% of adults and adolescents with asthma in a 24-week trial. 83

Table 8. Ivermectin 600 Dosing Schedule 88

Table 9. Montelukast sodium adverse reactions occurring at a higher incidence than placebo, in ≥1% of adults and adolescents ≥ 15 years of age. 93

Table 10. Ongoing clinical trials with montelukast 96

1. Protocol Summary

1.1. Synopsis

Title	ACTIV-6: COVID-19 Outpatient Randomized Trial to Evaluate Efficacy of Repurposed Medications
Clinical study phase	III
Rationale	Coronavirus Disease 2019 (COVID-19) is caused by a novel coronavirus, severe acute respiratory syndrome coronavirus 2 (SARS-CoV-2), that first emerged in December 2019 and has since resulted in a global pandemic unseen in almost a century in cases and mortality. Since 2020, advances have been made for treatment of COVID-19 and vaccination for prevention of SARS-CoV-2 infection. However, the pandemic continues to evolve with new variants and surges of infections in different regions of the world, requiring an ongoing evidence-generating clinical trial platform, in particular for the treatment of COVID-19 in the outpatient setting. This platform protocol can serve as an evidence generation system for prioritized drugs, repurposed from other Food and Drug Administration (FDA) indications with an established safety record in humans and preliminary data of efficacy. The ultimate goal is to evaluate if repurposed medications can make participants feel better faster and reduce death and hospitalization.
Primary Objective	• To evaluate the effectiveness of repurposed medications [(study drug(s)] in nonhospitalized participants with mild to moderate COVID-19
Secondary Objectives	• To evaluate the clinical outcomes in participants in a study drug arm versus those in the placebo arm<br>• To describe symptom resolution in participants in a study drug arm versus those in the placebo arm<br>• To describe the quality of life (QOL) in participants in a study drug arm versus those in the placebo arm<br>• To compare illness severity trajectories in participants in a study drug arm versus those in the placebo arm
Exploratory Objectives	• To describe long-term COVID-19-related symptoms in participants in a study drug arm versus those in the placebo arm

Intervention	All interventions will occur in addition to standard of care. Each study drug appendix describes a different study drug and matching placebo. The following arms will be included in each study appendix:<br>• Study Drug Arm: repurposed medications (see Appendices)<br>• Placebo Arm: placebo control<br>*While each appendix describes the placebo that matches the study drug, for comparative analysis the control group will comprise eligible, concurrently enrolled participants from all study arms who were assigned to placebo.*
Study Design	This study is a platform protocol designed to be flexible so that it is suitable for a wide range of settings within healthcare systems and in community settings where it can be integrated into routine COVID-19 testing programs and subsequent treatment plans. This platform protocol will enroll participants in an outpatient setting with a confirmed positive polymerase chain reaction (PCR) or antigen test for SARS-CoV-2. Each appendix will describe a repurposed medication (study drug) to meet the protocol objectives.<br>When only one study drug/appendix is under study, allocation between study drug and placebo will be 1:1. If multiple study drugs/appendices are under study, participants will also be randomized among the study drugs for which eligibility is confirmed. Since the route of administration of each study drug may differ, the placebos may also differ. To achieve blinding and an equitable randomization probability, a two-step randomization process will be used.<br>In the first step, the participant will be randomized m:1 active study drug to placebo, where m is the number of active study drugs for which the participant is eligible (note, if the same study drug is tested at multiple doses, each dose will count as one study drug). Then, participants will be randomized among the m study drugs for which they are eligible. Participants will carry their 'study drug' versus 'placebo' randomization with them into the study drug appendix. In this way, a participant allocated to placebo who is randomized to study drug A will be given the placebo that matches study drug A. This achieves equal probability of exposure among the placebo and active study drugs for which the participant is eligible, and equitable distribution among all study arms for which a participant is eligible. Sites will be informed to which study drug appendix the

	participant is randomized, but not whether they are allocated to the study drug arm or placebo arm within that appendix.<br>For analysis, concurrent placebo participants who were eligible for the study drug appendix will be pooled. This will result in approximately a 1:1 allocation ratio for any study drug to placebo. If a study drug appendix is stopped for efficacy and becomes standard of care, the active study drug arm may serve as a concurrent placebo for other study drugs.<br>Each study drug appendix will go through Screening Interim Analyses to assess efficacy/futility prior to evaluation of the primary objective. This Screening Interim Analysis provides an innovative approach to evaluate the potential for repurposed drugs to reduce symptom burden and prevent disease progression in the outpatient setting at various points throughout enrollment.<br>Participants will receive complete supply of repurposed medication (study drug) or placebo with length of treatment and amount of study drug/placebo depending on the study drug appendix and arm to which they are randomized.<br>This study is designed so that it can be done completely remotely. However, screening and enrollment may occur in-person at sites and unplanned study visits may occur in-person or remotely, as deemed appropriate by an investigator for safety purposes. Participants will be on-study for 120 days[1], during which they will complete various questionnaires.
Population	Up to 15,000 adults
Study Duration	24 months
Study Location	Up to 280 sites
Inclusion Criteria	1. Completed Informed Consent<br>2. Age ≥ 30 years old<br>3. Confirmed SARS-CoV-2 infection (or reinfection) by any authorized or approved PCR or antigen test collected within 10 days of screening<br>4. Two or more current symptoms of acute infection for ≤ 7 days. Symptoms include the following: fatigue, dyspnea, fever, cough,

[1] 120 days on-study was implemented after closing arms Ivermectin 400, Fluvoxamine Maleate, and Fluticasone Furoate; in those arms, participants were on study for 90 days.

	nausea, vomiting, diarrhea, body aches, chills, headache, sore throat, nasal symptoms, new loss of sense of taste or smell.
Exclusion Criteria	1. Current or recent (within 10 days of screening) hospitalization for COVID-19 infection<br>2. Current or planned participation in another interventional trial to treat COVID-19, at the discretion of the study principal investigator (PI)<br>3. Current or recent use (within the last 14 days) of study drug or study drug/device combination*<br>4. Known allergy/sensitivity or any hypersensitivity to components of the study drug or placebo*<br>5. Known contraindication(s) to study drug including prohibited concomitant medications (see Appendices)*<br>**If only one study drug appendix is open at the time of enrollment. If multiple study drug appendices are open, a participant may opt-out of any study drug appendix or be excluded from any study drug appendix based on contraindications listed in the study drug appendix, current use of study drug, or known allergy/sensitivity/hypersensitivity and still remain eligible for the remaining study drug appendices.*
Sample Size Considerations	This study will enroll up to 15,000 adults, depending on the number of study drug appendices that are added and adjustments to sample size depending on the data.<br>An estimated sample size of approximately 1,200 participants per study drug appendix is expected to be sufficient to conclude whether there is meaningful evidence of benefit. A screening interim analyses (sIA) will occur at n=300 and n=600 to inform termination of the arm, continuation of enrollment, or transition to assessment of the primary objective. Interim analyses (IAs) of the primary objective are planned at n=300, n=600 and n=900.<br>As described in the statistical analysis plan (SAP), the type I error for the primary objective is controlled at < 0.05.
General Statistical Consideration for Primary Analysis	The primary objective of effectiveness will be determined based on the endpoints of hospitalization/death or time to recovery over 28 days, the choice of which will be specified per appendix. The choice will be documented prior to interim analyses or prior to unblinding. The choice will be guided by emerging data on study drugs in the platform and on overall event rates in the trial, as well

	as external drivers, such as case rates, availability of other effective therapies, and vaccine effectiveness.<br><br>The outcomes of interest for this platform (symptoms, hospitalization, and mortality) are collected using a web-assisted symptom diary according to the schedule in **Table 1**. Symptoms will be graded on an ordinal scale as none, mild, moderate, or severe.<br><br>The risk difference in hospitalization or death will be used to draw conclusions about clinical events. Time to recovery, measured as the time to achieving three consecutive days of self-reported symptom freedom, will be used to draw conclusions about symptom burden.<br><br>The primary analysis will be implemented separately for each study drug, where the matching placebo arm will consist of concurrently randomized participants that meet the inclusion and exclusion criteria for that study drug appendix. A modified intention to treat (mITT) approach will be used for primary analyses; all participants who receive study drug will be included as assigned. It is possible that the delivery of medications (placebo or study drug) does not occur (failure of delivery, participant death, or participant withdrawal); this will result in exclusion of the participant for the mITT analysis. All available data will be used to compare each study drug versus placebo control, regardless of post-randomization adherence to study protocols.
Independent Data Monitoring Committee (IDMC)	Frequent IDMC reviews will be conducted to ensure the safety of study participants and evaluate the accumulating endpoint data by treatment group. Regular IDMC meetings will monitor the following parameters at a minimum:<br>• Recruitment progress<br>• Enrollment overall and by subgroups<br>• Adherence, retention, and status of data collection<br>• Serious adverse events<br>• Assessment for futility<br>• Probability for benefit across endpoints
Interim Analysis	Interim analyses (IA) will be performed per study drug appendix, after approximately every 300 participants (~150 in study drug arm and ~150 in placebo arm) have completed the Day 14 Visit. Placebo control participants contributing to this count will be drawn from across study drug appendices, and will include participants who were eligible for the study drug appendix of interest regardless

	of final study drug arm allocation. The following decision thresholds will be checked during IA(s):<br>i) Screening IA (n=300):<br>a. The study drug is found to have benefit (efficacy). Study drug appendix will proceed to primary objective IA at n=300. *Note: this is also a check for harm as all assessments are two-tailed.*<br>b. The study drug is not found to have benefit, enrollment continues in the study drug appendix and sIA is repeated at n=600.<br>ii) Screening IA (n=600):<br>a. It would be futile to attempt to show a benefit of the study drug based on the predicted probability of success (PPOS) and other factors. The study drug appendix will be terminated.<br>b. Futility is not determined. Study drug appendix will proceed to primary objective IA at n=600.<br>iii) Primary Objective IA (n=300): if the criteria for proceeding to the primary objective are met when n=300, a primary objective IA will be conducted for the primary objective when n=300. The following decisions will be assessed:<br>a. The study drug is found to have benefit (efficacy), the study drug appendix will be terminated as the primary endpoint has been met.<br>b. It would be futile to attempt to show a benefit of the study drug based on the PPOS and other factors. The study drug appendix will be terminated.<br>c. Efficacy/futility is undeterminable, enrollment will continue in the study drug appendix and the primary objective IA will be assessed at n=600.<br>iv) Primary Objective IA (n=600, 900): if the criteria for proceeding to the primary objective IA are met when n=600 or n=900, a primary objective IA will be conducted. The following decisions will be assessed:<br>a. The study drug is found to have benefit (efficacy), the study drug appendix will be terminated as the primary endpoint has been met.<br>b. It would be futile to attempt to show a benefit of the study drug based on the PPOS and other factors. The study drug appendix will be terminated.

	c. Efficacy/futility is undeterminable, enrollment will continue in the study drug appendix and the primary objective will be assessed after another 300 participants have been enrolled, or until n=1200.<br><br>A posterior probability of meaningful benefit for a study drug in comparison to the placebo control of greater than the appendix-specified threshold will result in a declaration of overall superiority. A PPOS when n=1200 of less than the appendix-specified threshold will result in a declaration of futility.<br><br>Futility is a low probability of achieving any conclusions within a reasonable time frame or based on other factors for the trial. Prior to each IA, the target date for study completion will be specified, and accrual will be projected by that target date. The PPOS of any study drug given expected accrual at a prespecified point in time will be provided to the IDMC. A statistical model may be used to predict accrual. Futility assessment will use the lowest of either the planned accrual or predicted accrual at study closure.

1.2. Schema

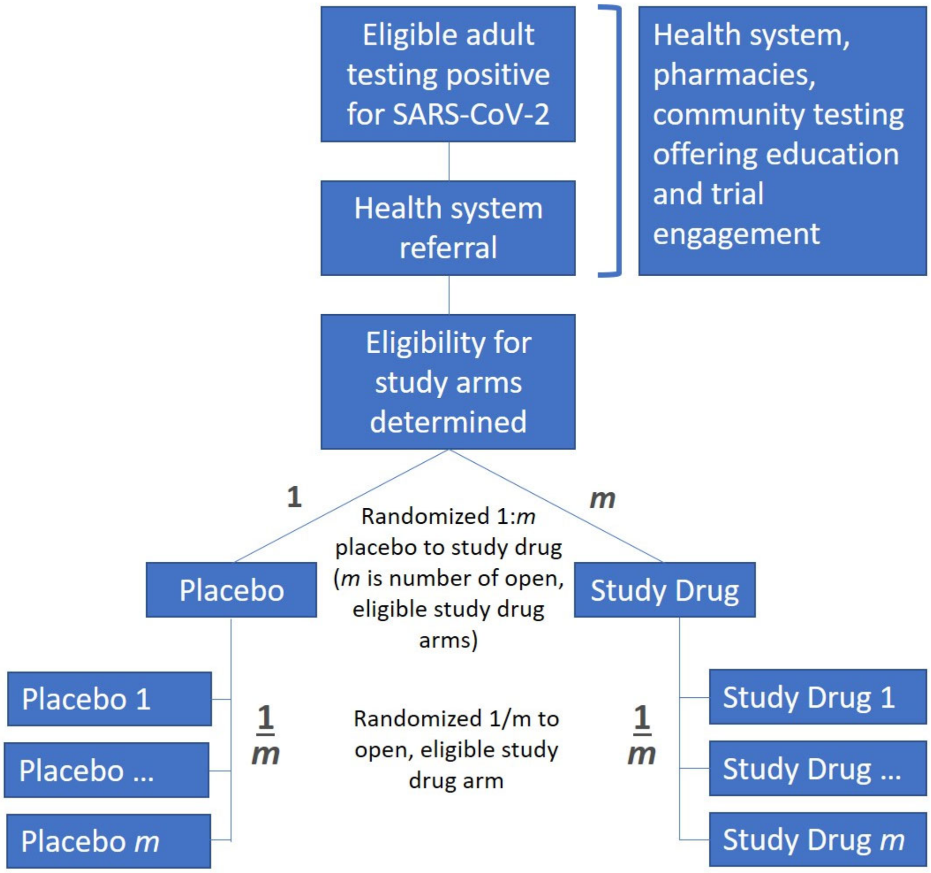

Figure 1: ACTIV-6 Study Schema

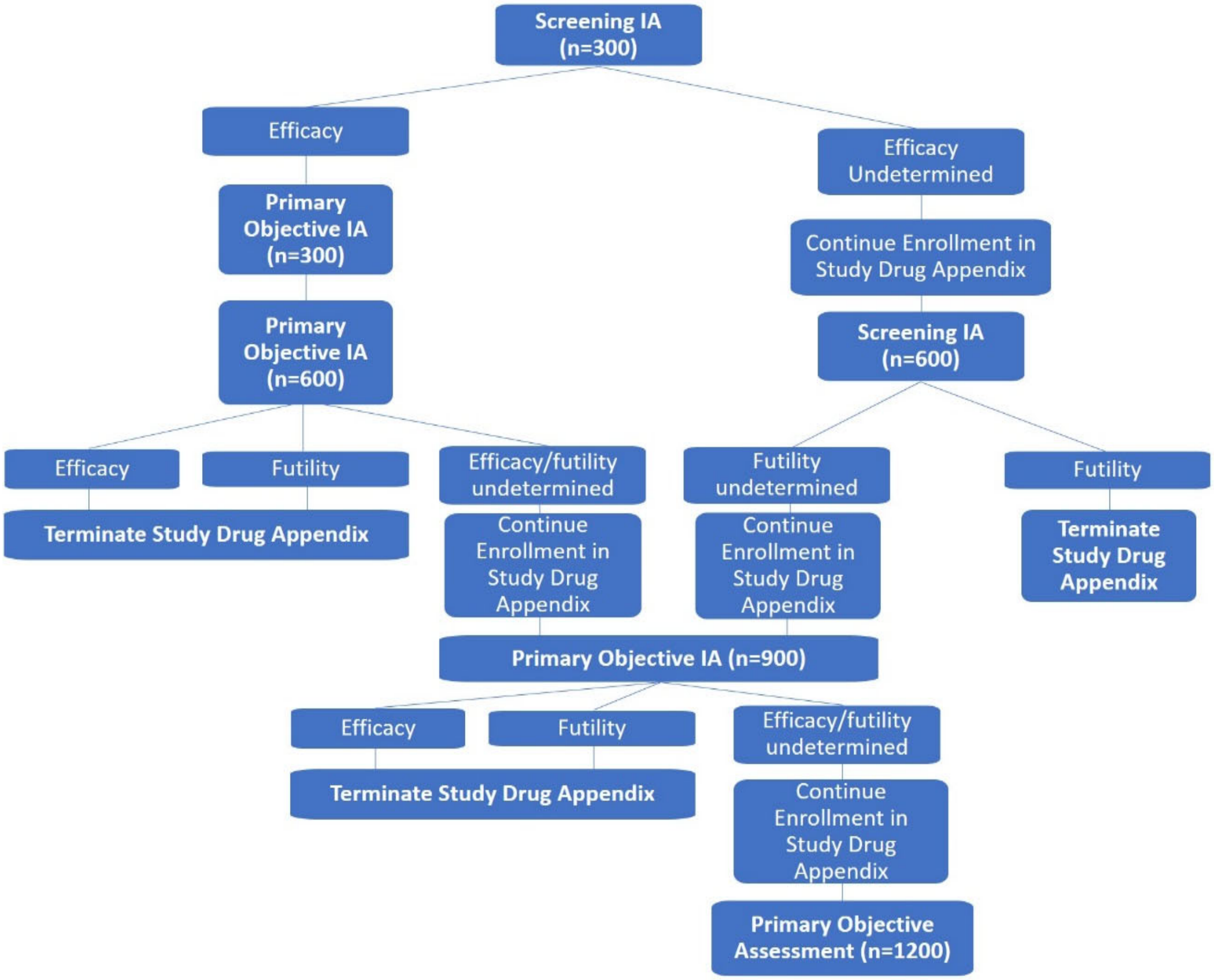

Figure 2. ACTIV-6 Interim Analysis Schema

2. Introduction

2.1. Study Rationale

Severe acute respiratory syndrome coronavirus 2 (SARS-CoV-2) is a novel betacoronavirus that first emerged in December 2019 and has since caused a global pandemic unseen in almost a century with respect to the number of cases and overall mortality.[1, 2] The clinical disease related to SARS-CoV-2 is referred to as Coronavirus Disease 2019 (COVID-19). Since 2020, advances have been made for treatment of COVID-19 and vaccinations for prevention of SARS-CoV-2 infection, through emergency use authorization or FDA approval.[3-8] However, the pandemic continues to evolve with new variants and surges of infections in different regions of the world, requiring an ongoing evidence-generating platform, in particular for the treatment of COVID-19 infection in the outpatient setting. This platform protocol operates in addition to usual care and can serve as an evidence generating system for prioritized drugs repurposed from other indications with an established safety record and preliminary evidence of clinical efficacy for the treatment of COVID-19. The ultimate goal is to evaluate if repurposed medications can make participants feel better faster and reduce death and hospitalization.

2.2. Background

In December 2019, numerous patients in Wuhan, China were diagnosed with pneumonia caused by an unknown virus. By January 7, 2020, Chinese scientists had isolated SARS-CoV-2. This is a novel betacoronavirus closely related to severe acute respiratory syndrome coronavirus 1 (SARS-CoV-1).[2] In the subsequent months the spread of the virus led to a global pandemic. As of February 7, 2022 there were approximately 395,540,912 confirmed COVID-19 cases resulting in 5,741,726 deaths worldwide.[1]

The virus continues to spread despite social distancing and masking measures, vaccination campaigns/requirements, and travel restrictions. COVID-19 vaccinations continue to be distributed and administered globally; however, new SARS-CoV-2 strains continue to emerge, with potential for reduced monoclonal antibody therapeutic and vaccine efficacy.[9] As new strains have emerged that confirm transmission, infection, and even severe disease after vaccination is possible, highlighting the need to establish treatment regimens despite vaccination availability. Furthermore, acceptance and uptake of booster shots has been lower than uptake of the initial vaccination series, further justifying the need for safe and effective therapies. Thus, there remains a need to identify safe and efficacious treatments that can be administered in the outpatient setting.

As of February 2022, multiple clinical trials have been reported, providing guidance to clinical providers on management of COVID-19, particularly in the hospital setting. Various drugs, including monoclonal antibodies and antivirals, have been authorized or approved by the FDA for use in the inpatient and outpatient setting for treatment of COVID-19. Multiple repurposed immunomodulatory agents are clinically used for the treatment of severe COVID-19 in the inpatient setting. Thus, multiple medications have been reported to improve clinical disease and in some cases, mortality.[10, 11] Few of these are FDA approved and few are therapies that can be administered at home.

2.3. Benefit/Risk Assessment

The risks for participation in this study include taking study drug (see Appendices) and loss of confidentiality. There may be some benefit to the participant if the therapy is effective against COVID-19.

2.3.1. Risk Assessment

Loss of confidentiality risks: There is a potential risk of loss of confidentiality. Every effort will be made to protect the participant's confidential medical information, but this cannot be guaranteed. Clinical information will not be released without written permission of the participant, except as necessary for monitoring by the IRB, FDA, National Institutes of Health (NIH), Office for Human Research Protections (OHRP), other local, US, and international regulatory authorities/entities as part of their duties.

Risk lowering measures: Study procedures to manage and minimize risks include careful selection of the participants and monitoring over time to check on participants' health. Additional guidance to manage any risks or any change to the risk to the participant based on emerging data will be provided to the study teams, as needed. In addition, an Independent Data Monitoring Committee (IDMC) will monitor safety of the participants throughout the study.

2.3.2. Benefit Assessment

Participants who randomize to a study drug arm may benefit from study drug administration. There is no direct benefit to participants randomized to the placebo arm apart from participating in generating evidence that may ultimately support treatment for SARS-CoV-2 infection. In addition, they will benefit from involvement with the team following their health status during the study. The knowledge gained will be a benefit to others in the future.

3. Objectives and Endpoints

Objectives	Outcome Measurements	Reported Endpoints
Primary		
To evaluate the effectiveness of repurposed medications [(study drug(s)] in nonhospitalized participants with mild to moderate COVID-19	• Hospitalization or death by Day 28<br>• Time to recovery over 28 days, wherein time to recovery is the time to achieving three consecutive days of self-reported symptom freedom	Relative risk for clinical events or difference in time to recovery will be used to estimate treatment effect.
Secondary		
To evaluate the clinical outcomes in participants in a study drug arm versus those in the placebo arm	• COVID Clinical Progression Scale on Day 7, Day 14, and Day 28 (see Section 8.2)<br>• Mortality through Day 28<br>• Hospitalization, urgent care visit, emergency room visit through Day 28	The following model-assisted endpoints will be reported for the COVID Clinical Progression Scale:<br>• The OR describing the overall difference in clinical progression<br>• The OR describing the difference in clinical progression at each measured time point<br>• The overall risk difference for hospitalization or death<br>The following endpoints for the composite of unscheduled medically assisted care will be directly assessed and reported:<br>• The overall risk difference for any of urgent care, emergency care, hospitalization or death

		• Time to first urgent care, emergency care, hospitalization or death<br>• Risk and time to event for each component of the composite
To describe symptom resolution in participants in a study drug arm versus those in the placebo arm	Symptom resolution, defined as three consecutive days without symptoms	Time to symptom resolution
To describe the quality of life (QOL) in participants in a study drug arm versus those in the placebo arm	Modified Patient-Reported Outcomes Measurement Information System (PROMIS)-29 at baseline, Day 7, Day 14, Day 28, Day 90, and Day 120[2] Follow-up	• Overall common odds ratio (cOR)<br>• Odds ratios (cORs) specific to days 7, 14, 28, 90, and 120[2]<br>• Mean difference in QOL scores at each time point
To compare illness severity trajectories in participants in a study drug arm versus those in the placebo arm	Ordinal outcome including symptom severity, hospitalization, and death measured daily for 14 days	• Difference in mean time unwell<br>• Mean days of benefit
Exploratory		
To describe long-term COVID-19-related symptoms in participants in a study drug arm versus those in the placebo arm	Symptom occurrence, type, and severity at Day 90, Day 120[2], or Day 180[2] Follow-up	Directly measured mean and median symptom count and QOL score at Day 90, Day 120[2], or Day 180[2] in study drug arm(s) versus placebo.

[2] Day 180 is applicable only for participants who were consented to the study after protocol v7.0 was implemented; Day 120 is applicable only for participants consented under protocol v6.0; Day 90 was the final follow-up day for Ivermectin 400, Ivermectin 600, Fluvoxamine Maleate, and Fluticasone Furoate.

4. Study Design

Refer to Section 1.2 for the Study Schema.

This study includes an innovative screening approach using sIAs to make decisions about dropping ineffective agents quickly, or to accelerate study of potentially effective agents. Each study drug appendix will go through sIAs to assess efficacy/futility prior to evaluation of the primary objective. This sIA provides an innovative approach to evaluate the potential for repurposed drugs to reduce symptom burden and prevent disease progression at various points throughout enrollment in a broad population.

4.1. Overall Design

This study is a platform protocol designed to be flexible so that it is suitable for a wide range of settings within healthcare systems and in community settings where it can be integrated into routine COVID-19 testing programs and subsequent treatment plans. The platform protocol will enroll participants with mild to moderate COVID-19 in an outpatient setting with a confirmed positive polymerase chain reaction (PCR) or antigen test for SARS-CoV-2 infection. Each appendix will describe a repurposed medication (study drug arm) that is sized to meet the master protocol objectives.

Participants will be randomized to one of the study drug appendices that are actively enrolling at the time of randomization. Study drug appendices may be added or removed according to adaptive design and/or emerging evidence. When there are multiple study drug appendices available, randomization will occur based on appropriateness of each drug for the participant as determined by the study protocol and investigator and participant equipoise. Each participant will be required to randomize to at least one study drug versus placebo. The probability of placebo to treatment will remain the same regardless of eligibility decisions.

Eligible participants will be randomized (1:1), in a blinded fashion, to either the study drug arm or placebo arm in addition to standard of care, when one active drug is on the platform or when a participant is only eligible for one of the active drugs on the platform. As additional study drug appendices are added, the randomization will be altered to leverage placebo data across arms. If a study drug is offered at two doses, each dose will be treated as separate study arm. If a participant is eligible for 1 study arm, they have a 1:1 chance of receiving an active study drug. If a participant is eligible for two study arms, they have a 2:1 chance of receiving an active study drug, for 3 arms it is 3:1, for 4 arms it is 4:1, and so on. This is because each participant assigned to a placebo group is shared among all appendices, and the goal is that within appendices the allocation probability to study drug versus placebo is 1:1. Participants will receive a complete supply of repurposed medication (study drug) or placebo with the quantity depending on the study drug/placebo to which they are randomized.

All study visits are designed to be remote. However, screening and enrollment may occur in-person at sites and unplanned study visits may occur in-person or remotely, as deemed appropriate by the site investigator for safety purposes. Participants will be asked to complete questionnaires and report safety events during the study, according to **Table 1**. Participants will be prompted by the online system to report safety events and these will be reviewed and confirmed via medical records and site staff, as necessary.

4.2. End of Study Definition

A participant is considered to have completed the study if he/she has completed the applicable Long-term Follow-up assessments, refer to Table 1.

The end of the study is defined as the date of the last follow-up of the last participant in the study. Data from interim analyses or recommendations by the IDMC may result in protocol modifications or early termination of the study.

5. Study Population

All Eligibility Criteria will be obtained per participant.

5.1. Inclusion Criteria

1. Completed Informed Consent
2. Age ≥ 30 years old
3. Confirmed SARS-CoV-2 infection (or reinfection) by any authorized or approved PCR or antigen test collected within 10 days of screening
4. Two or more current symptoms of acute infection for ≤7 days. Symptoms include the following: fatigue, dyspnea, fever, cough, nausea, vomiting, diarrhea, body aches, chills, headache, sore throat, nasal symptoms, new loss of sense of taste or smell

5.2. Exclusion Criteria

1. Current or recent (within 10 days of screening) hospitalization for COVID-19 infection
2. Current or planned participation in another interventional trial to treat COVID-19, at the discretion of the study principal investigator (PI)
3. Current or recent use (within the last 14 days) of study drug or study drug/device combination*
4. Known allergy/sensitivity or any hypersensitivity to components of the study drug or placebo*
5. Known contraindication(s) to study drug including prohibited concomitant medications (see Appendices)*

   **If only one study drug appendix is open at the time of enrollment. If multiple study drug appendices are open, a participant may opt-out of any study drug appendix or be excluded from any study drug appendix based on contraindications listed in the study drug appendix, current use of study drug, or known allergy/sensitivity/hypersensitivity and still remain eligible for the remaining study drug appendices.*

5.3. Recruitment and Engagement

5.3.1. Participant Recruitment

Participants who are eligible based on positive SARS-CoV-2 PCR or antigen test will be identified by participating sites or will self-identify to a central study hotline(s) and be referred to the closest site. Site investigators, or their designee, may contact eligible participants to introduce the study and discuss study participation.

5.3.2. Participant Engagement

Participants will be engaged in the study through multiple channels. This includes, but is not limited to, ongoing participation in other registries partnering with ACTIV or healthcare systems. Additionally, participant engagement will include:

- compensation for participants who complete the applicable Final Visit (Day 90, 120, or 180);
- creating a study-wide ACTIV-6 Advisory Group;
- developing participant-centered approaches that recognize the needs and preferences of COVID-19 survivors locally and nationally; and
- multifaceted approaches that combine engagement tools, leverage the online system, use of social media, and representative COVID-Participants.

5.3.3. Participant Randomization Process

This trial is a double-blind, placebo-controlled trial. Participants and investigators will be blinded. A participant who is eligible for m study drug arms/appendices will be randomized m:1 study drug to placebo (**Figure 1**). The participant will then be randomized with $1/m$ probability to each of the study drug appendices. A participant entering a study drug appendix carries their study drug or placebo designation with them and will get either the study drug or matching placebo. Participants who receive placebo will be pooled across study drug arms for those study drug arms/appendices that the participant is eligible. This reduces overall sample size by facilitating sharing of data from concurrent controls while maintaining a 1:1 allocation to study drug or placebo within an appendix. Randomization sequences will not be pre-generated. Given the adaptive nature of the trial and the unknown number of study drug appendices, arm assignment will be implemented at the time of confirming eligibility for randomization using a random number generator. The participant eligibility criteria will be checked for each study drug appendix, and the randomization probabilities will be set. The two step procedure will then occur, and the assignment to both study drug appendix and study drug versus placebo will be made. The participant and study teams will know which study drug appendix the participant is allocated to, but will be blinded to study drug versus placebo because they will be matching.

The participant, treating clinicians, and study personnel will remain blinded to study drug versus placebo assignment until after the database is locked and blinded analysis is completed. Only the biostatistical team who is preparing closed IDMC interim reports will be unblinded. Specifically, study drug/placebo will be dispensed with packaging and labelling that would blind treatment assignment. Unblinding will occur only if required for participant safety or treatment at the request of the treating clinician. Refer to the Manual of Procedures (MOP) for further details.

5.4. Screen Failures

Screen failures are defined as participants who consent to participate in the clinical study, who fulfill inclusion and exclusion criteria, but are not subsequently randomized. Screen failures also include participants who consent, then on review by the site, are found to be ineligible for the study. A minimal set of screen failure information is required to ensure transparent reporting of screen failure participants to meet the Consolidated Standards of Reporting Trials (CONSORT) publishing requirements and to respond to queries from regulatory authorities.

Individuals who are considered screen failures may not be re-screened.

5.5. Enrollment

Participants who are randomized and receive study drug/placebo will be considered enrolled. Participants who are randomized, but do not receive study drug/placebo for any reason (e.g., study drug lost in the mail, death prior to receipt of study drug, participant withdrawal prior to receipt of study drug), will not be considered enrolled on the study and will be identified as *randomized not enrolled*.

Receipt of study drug will be defined as evidence that the study drug was delivered to the address of record.

6. Study Drug(s)

6.1. Repurposed Medication Treatments

See Appendices

6.2. Placebo

See Appendices

6.3. Study Drug Accountability

Use of study drug will be tracked via the online system, call center, or sites. Participants will dispose of any unused study drug as they would normally when stopping a medication.

6.4. Concomitant Therapy

Select concomitant medications of interest that the participant is receiving at the time of enrollment or receives during the course of the study will be recorded along with dosing information. Select concomitant medications of interest include the following and will be verified at each remote visit by designated study personnel:

- Any therapeutics that is thought to have potential or purported COVID activity including hydroxychloroquine
- Antibiotics
- Antifungals
- Antiparasitic
- Antivirals including HIV protease inhibitors and ribavirin
- Immunosuppressants including steroids
- Angiotensin-converting-enzyme (ACE)/angiotensin II receptor blockers (ARB)/angiotensin receptor neprilysin inhibitor (ARNI)
- Statin
- Anticoagulants and antiplatelets
- COVID-19 vaccine (before, during, or after study intervention)

Refer to the MOP for more details on concomitant therapy. Refer to the appendices for contraindicated medications for each of the study drugs.

6.5. Intervention After the End of the Study

No additional study drug will be provided to the participant following completion of the study.

7. Participant Withdrawal/Termination and Study Termination

7.1. Participant Withdrawal/Termination

Participants will be followed until participant closeout, withdrawal of consent, or death.

A participant may withdraw from the study at any time at his/her own request, or may be withdrawn at any time at the discretion of the investigator for safety, behavioral, compliance, or administrative reasons. This is expected to be uncommon.

Those who request withdrawal from the study will be asked to continue on study follow-up with limited participation through the Final Visit (Section 8.1.4). Limited participation may include a call(s) to assess safety at study visits following withdrawal.

If the participant withdraws consent for disclosure of future information, the sponsor may retain and continue to use any data collected before such a withdrawal of consent.

7.2. Premature Termination or Suspension of the Study

The study may be temporarily suspended or prematurely terminated if there is sufficient reasonable cause. Written notification will be provided documenting reason for study suspension or termination to the investigators, funding agency, and regulatory authorities, as appropriate. Circumstances that may warrant termination or suspension include, but are not limited to:

- Determination of unexpected, significant, or unacceptable risk to participants
- Insufficient compliance to protocol requirements
- Data that are not sufficiently complete and/or evaluable
- Determination of futility after a sufficient time has passed for accrual of the primary and secondary outcomes
- Recommendation by the IDMC

7.3. Lost to Follow-up

Participants will be asked for proxy contacts to assess vital status and/or other clinical events, including safety, if a participant fails to provide the information. Provision of proxy information is not required for study participation. A participant will be considered lost to follow-up if he or she repeatedly fails to complete study assessments/procedures as outlined below and neither the participant nor the participant's proxy can be contacted by the study site.

The following actions must be taken if a participant fails to provide baseline information, if he or she fail to complete daily symptom reporting by midnight the day after receiving the first dose of study drug/placebo (Day 2), if he or she miss one daily symptom reporting during Days 3 to 14, if he or she miss either the Day 14 or Day 28 Remote Visits, and/or he or she fail to complete the applicable Final Visit assessments:

- The site or call center must attempt to contact the participant and counsel the participant on the importance of completing study assessments/procedures.
- The site or call center will contact the participant's proxy to assess vital status and/or other clinical or safety events.
- The site or call center will attempt to collect all missing survey responses.

- Before a participant is deemed lost to follow-up, the investigator or designee must make every effort to regain contact with the participant (where possible, three telephone calls and, if necessary, a certified letter to the participant's last known mailing address or local equivalent methods). These contact attempts should be documented in the participant's research record.
- Online obituary search.
- Should the participant continue to be unreachable, they will be considered lost to follow-up.

8. Study Assessments and Procedures

Screening and eligibility confirmation will be participant-reported. A positive SARS-CoV-2 test result must be verified prior to randomization (refer to the MOP for details). Sites will be responsible for notifying the coordinating center for participant withdrawals, lost to follow-up, permanent cessation of study drug, study drug dose modifications (if allowed, per Appendix), or change in vital status. Data will be collected directly from the participant and supported by medical records, as needed.

8.1. Schedule of Events

Table 1: Schedule of Events

	Screening	Intervention Period		Follow-up Period						Final Visit	Unplanned Study Visit
**Day**	**Within 2 days of Day 1**	**Day 1**	**Days 2 - 14**	**Day 15 - 20**	**Day 21 ± 2**	**Day 22 - 27**	**Day 28 + 5**	**Day 90[3] + 5**	**Day 120[3] + 5**	**Day 180[3] + 7**	
**ACTIV-6 Trial**											
Consent	X										
Demographic Information	X										
Eligibility criteria confirmed	X										
Randomization	X										
Receipt of study drug or placebo		X									
Continued use study drug		Continuous[4]									
**Clinical Assessments**											
Abbreviated medical history	X										
Self-reported Pregnancy	X[5]										
Concomitant Therapy	X	X[6]	X[6]								
Remote Visit			X[7]				X				
Drug Adherence		X	X								
COVID-19 Outcomes		X	X[9]		X		X	X	X	X	
Symptom Reporting	X[8]	X	X[8]	X[8]	X[8]	X[8]	X[8]	X	X	X	
PASC Symptom Questionnaire										X	
QOL Questionnaire	X		X[9]				X	X	X	X	
At-home pulse oximetry			X[10]								
Columbia-Suicide Severity Rating Scale (C-SSRS)			X[11]								
Safety Assessment[12]		Continuous via online system and medical record review									X

[3] Day 180 is applicable only for participants who were consented after protocol v7.0 was implemented; Day 120 is applicable only for participants consented on protocol v6.0; Day 90 was the final follow-up day for Ivermectin 400, Ivermectin 600, Fluvoxamine Maleate, and Fluticasone Furoate

[4] Refer to study drug appendix for length of study drug administration.

[5] Only for enrollment in Study Drug Appendices that have pregnancy listed as a contraindication for females of childbearing potential. Participants will self-report pregnancy using the Pregnancy Reasonably Excluded Guide.

[6] Review only during study drug/placebo administration if contraindicated medications provided for the study drug arm, per Appendix.

[7] Day 14 only.

[8] Daily symptom reporting; continued daily beyond day 14 through day 28 until symptoms resolve for ≥ 3 consecutive days. All participants will complete symptom reporting on Days 21 and 28, regardless of symptom resolution.

[9] Day 7 and 14 only.

[10] Day 3, 7, and 14 only.

[11] At Day 7 and 14 for participants enrolled in Appendix E – Fluvoxamine Maleate 100.

[12] Participant's medical record will be reviewed to confirm Serious Adverse Events (SAEs), Unanticipated Adverse Device Events (UADEs) [as applicable], and Events of Special Interest (ESIs).

8.1.1. Screening

The following events will occur at Screening:

- Consent: Participants will be consented either via an e-consent process or paper process. The consent process should be done in accordance with local and central IRB regulations. Phone consenting may be facilitated through the e-consent or paper process.
- Demographic information will be collected including, but not limited to, age, sex, race, ethnicity, and occupation
- Eligibility criteria confirmation by the participant via the online system or by site staff via a paper process
- Abbreviated medical history
- Self-reported pregnancy, for women of childbearing potential (**only for enrollment in Study Drug Appendices that include pregnancy as a contraindication**)
- Concomitant therapy
- Symptom reporting, daily during screening period
- QOL questionnaire
- Randomization (see Section 5.3.3)

8.1.2. Intervention Period

The following events will occur during the Intervention Period, starting with receipt of study drug/placebo:

Day 1:

- Receipt of study drug or placebo
- Study drug self-administration (see Appendices for specific study drug/placebo administration)
- Concomitant therapy
- Drug adherence questionnaire
- COVID-19 Outcomes
- Symptom reporting
- SAE, UADE (as applicable), and ESI collection

Days 2 – 14:

- Study drug self-administration (see Appendices for specific study drug/placebo administration)
- Concomitant therapy, including contraindicated medications provided for the study drug arm, per Appendix, during study drug/placebo administration.
- Remote visit (**Day 14 only**)
- Drug adherence questionnaire, daily
- COVID-19 Outcomes (**Day 7 and 14 only**)
- Symptom reporting, daily
- QOL questionnaire (**Day 7 and 14 only)**
- At-home pulse oximetry readings (**Day 3, 7, and 14 only**)

- C-SSRS (**Day 7 and 14 for Appendix E – Fluvoxamine Maleate 100 only**)
- SAE, UADE (as applicable), and ESI collection

8.1.3. Follow-up Period

Day 15 – 20:

- Symptom reporting, daily from Day 14 for participants who have **not** yet reported three consecutive days of no symptoms. Participants who experience three days of improvement before Day 14 but who then experience symptoms again will not be followed daily.
- SAE, UADE (as applicable), and ESI collection

Day 21 ± 2 days:

- COVID-19 Outcomes
- Symptom reporting
- SAE, UADE (as applicable), and ESI collection

Day 22 – 27:

- Symptom reporting, daily from Day 14 for participants who have **not** yet reported three consecutive days of no symptoms. Participants who experience three days of improvement before Day 14 but who then experience symptoms again will not be followed daily.
- SAE, UADE (as applicable), and ESI collection

Day 28 + 5 days:

- Remote visit
- COVID-19 Outcomes
- Symptom reporting
- QOL questionnaire
- SAE, UADE (as applicable), and ESI collection

Day 90 + 5 days:

- COVID-19 Outcomes
- Symptom reporting
- QOL questionnaire
- SAE, UADE (as applicable), and ESI collection

The Day 120 + 5 days Follow-up visit is only applicable for participants consented on protocol v6.0 or later and will include the following:

- COVID-19 Outcomes
- Symptom reporting
- QOL questionnaire
- SAE, UADE (as applicable), and ESI collection

8.1.4. Final Visit

Depending on when the participant was consented, the Final Visit may have occurred at Day 90 or Day 120. Following implementation of protocol v7.0, Day 180 + 7 days will serve as the Final Visit. The Final Visit will include the following:

- COVID-19 Outcomes
- Symptom reporting
- Post-acute Sequelae of SARS-CoV-2 Infection (PASC) Symptom Questionnaire (**Day 180 participants only**)
- QOL questionnaire
- SAE, UADE (as applicable), and ESI collection

8.2. Clinical Assessments

Abbreviated Medical History: smoking status, estimated body mass index (BMI)/obesity, pre-existing underlying lung disease (e.g., chronic obstructive pulmonary disease, asthma, idiopathic pulmonary fibrosis), underlying immunosuppression (transplant, malignancy, human immunodeficiency virus (HIV), autoimmune disease), medical conditions that may increase risk of COVID-19 infections or complications (e.g., diabetes, cardiovascular disease, hypertension, chronic kidney disease), venous thromboembolism, chronic liver disease, COVID-19 vaccination status

Concomitant Medications of Interest: Concomitant medications of interest, including study drug specific contraindicated medications, will be collected. Refer to Section 6.4 for concomitant medications of interest and to the study drug specific appendices for contraindicated medications.

Self-reported Pregnancy: Participants will be asked to self-report pregnancy, as needed, per Study Drug Appendix. The 3-item "Pregnancy Reasonably Excluded Guide" will be used to assess pregnancy at screening. The "Pregnancy Reasonably Excluded Guide" uses traditional and World Health Organization criteria to exclude pregnancy via participant self-report.[12] Refer to the MOP for details.

Remote Visit: Designated study personnel will contact the participant directly via a phone call or other form of direct contact (e.g., text or e-mail survey) in order to conduct study assessments, including, but not limited to, COVID-19 outcomes, drug adherence (at Day 14), and safety events. A missed remote visit will be considered a protocol deviation (non-major). If a participant misses a remote visit, site study staff should take immediate action to contact the participant, per Section 7.3 follow-up processes. Refer to the MOP for details.

Drug Adherence: Adherence to the study drug administration schedule will be collected via the online system and confirmed at the Day 14 remote visit.

COVID-19 Outcomes: The COVID-19 outcomes for this trial are based on the World Health Organization's Ordinal Scale for Clinical Improvement and will be collected via the online system and from the medical record.[13] The following outcomes will be assessed as part of the COVID Clinical Progression Scale:

0. No clinical or virological evidence of infection

1. No limitation of activities
2. Limitation of activities
3. Hospitalized, no oxygen therapy
4. Hospitalized, on oxygen by mask or nasal prongs
5. Hospitalized, on non-invasive ventilation or high-flow oxygen
6. Hospitalized, on intubation and mechanical ventilation
7. Hospitalized, on ventilation + additional organ support – pressors, RRT, ECMO
8. Death

Symptom Reporting: Symptoms and symptom-related responses will be reported by the participant via the online system. Additional symptom reporting may occur from the sites, as available. Each of pre-defined symptoms will be assessed on an ordinal severity scale of none, mild, moderate, and severe. The following symptoms will be collected:

- Overall symptom burden
- Fatigue
- Dyspnea - shortness of breath or difficulty breathing at rest or with activity
- Fever
- Cough
- Nausea
- Vomiting
- Diarrhea
- Body aches
- Sore throat
- Headache
- Chills
- Nasal symptoms
- New loss of sense of taste or smell
- Other COVID-related symptom

Post-acute Sequelae of SARS-CoV-2 Infection (PASC) Symptom Questionnaire: COVID-19 has affected many lives through lingering symptoms, often debilitating long after acute SARS-CoV-2 infection. The syndrome of PASC is a chronic condition present in up to 80% of infected, hospitalized patients and 40% to 70% of non-hospitalized patients. [14-18] The PASC Symptom Questionnaire includes symptoms that are associated with PASC and asks for severity (mild/moderate/severe) related to any symptoms identified by the participants.

At-home pulse oximetry measurements: Participants will provide pulse oximetry readings using a study-provided FDA-approved pulse oximeter. Two consecutive pulse oximetry readings must be reported at each required time point. Day 3, 7, and Day 14 are study-required time points for pulse oximetry readings. Participants can report pulse oximetry readings at other times throughout the study, at their own discretion.

8.3. Quality of Life Questionnaires

The following QOL questionnaire will be used in this study:

- Modified PROMIS-29: PROMIS measures were developed through a collaborative process funded by the NIH.[19] The PROMIS-29 consists of seven health domains with four 5-level items associated with each and a pain intensity assessment using a 0-10 numeric rank. The seven health domains include physical function, fatigue, pain interference, depressive symptoms, anxiety, ability to participate in social roles and activities, and sleep disturbance.[20] The PROMIS-29 measures will be modified for this study and will include select questions from each of the seven health domains, refer to the MOP for details.

9. Safety Assessments

9.1. Adverse Events and Serious Adverse Events

An AE is any untoward medical occurrence in humans, whether or not considered drug-related, which occurs during the conduct of a clinical trial. An AE can therefore be any change in clinical status, routine labs, x-rays, physical examinations, etc., that is considered clinically significant by the study investigator.

An SAE or serious suspected adverse reaction or serious adverse reaction as determined by the investigator or the sponsor is an AE that results in any of the following serious outcomes:

- Death
- Life-threatening AE ("life-threatening" means that the study participant was, in the opinion of the investigator or sponsor, at immediate risk of death from the reaction as it occurred and required immediate intervention)
- Persistent or significant incapacity or substantial disruption of the ability to conduct normal life functions
- Inpatient hospitalization or prolongation of existing hospitalization
- Congenital abnormality or birth defect
- Important medical event that may not result in one of the above outcomes, but may jeopardize the health of the study participant or require medical or surgical intervention to prevent one of the outcomes listed in the above definition of serious event

Hospitalization for elective treatment of a preexisting condition that did not worsen from baseline does not meet the definition of an SAE. Hospitalization is defined as a stay in the hospital exceeding 24 hours.

An unexpected AE is defined as any AE, the specificity or severity of which is not consistent with the package insert.

9.1.1. Adverse Device Effect (ADE) and Unanticipated Adverse Device Effect (UADE)

For those repurposed medications that are a part of a combination product, which includes a drug and a device, the following additional definitions will apply.

An ADE is an AE related to the use of an investigational medical device. This includes any AE resulting from insufficiencies or inadequacies in the instructions for use, the deployment, the implantation, the installation, the operation, or any malfunction of the repurposed medical device. This also includes any event that is a result of a use error or intentional misuse.

- Device malfunction – the failure of a device to perform in accordance with the instructions for use or clinical investigative plan.
- User error or intentional misuse – A device is used in a manner that is an act or omission of an act that results in a different medical device response than intended by the manufacturer or expected by the user.

A UADE is any SAE caused by, or associated with, a device, if that effect, problem, or death was not previously identified in nature, severity, or degree of incidence in the investigational plan or applications (including a supplemental plan or application), or any other unanticipated

serious problem associated with a device that relates to the rights, safety, or welfare of participants.

Unanticipated Adverse Device Effects (UADEs) will include events meeting either A or B as stated below:

A. Events meeting ALL of the following criteria:
   - Not included in the relevant appendices or Product Label
   - Related to the device per site PI and/or IND sponsor
   - Serious (meets any of the following criteria):
     - Is life-threatening illness or injury
     - Results in permanent impairment of a body function or a body structure
     - Necessitates medical or surgical intervention to prevent permanent impairment of a body function or a body structure
     - Results in hospitalization
     - Led to fetal distress, fetal death, or congenital abnormality or birth defect
     - Led to death

(*Permanent means irreversible impairment or damage to a body structure or function, excluding trivial impairment or damage*).

B. Any other unanticipated serious problem associated with the device that relates to the rights, safety, or welfare of participants.

9.1.2. Collection Period for AE and SAE Information

Study participants (and their designated emergency proxies) will be instructed to report ESIs per appendix and Section 9.1.5, SAEs, and UADEs through their access to the study's online system. Each day for 14 days, the participant will be asked to report on their symptoms and health state, including hospitalization and/or other change in health condition. The assessments include specific questions pertaining to ESIs, as well as symptoms and severity, health care visits, medications and a notification to the participant to contact the study team with any concerns or questions. If the participant is still reporting symptoms at Day 14, they will continue to be assessed until they have experienced three consecutive days without symptoms, or until Day 28, whichever is shorter. At Day 28, Day 90, Day 120, and Day 180, participants will complete assessments. Safety reporting will be available to the participant continuously throughout the study, but will only be required at the aforementioned collection points.

The daily and follow-up assessments, as described in the paragraph above, will be monitored and sites will be actively notified of events requiring review, including for reporting that meets criteria for ESIs, SAEs, or UADEs. Refer to the MOP for details. In addition, participants will be invited during assessments to request contact from the study team, or to report any unusual circumstances that might be relevant, if they so wish. Failure to complete daily assessments is also a trigger for review of a possible SAE. A missed assessment on the day after receiving the

first dose of study medication (Day 2) or any day of missed assessments up to Day 14 will prompt a notification to the site to contact the participant.

All participants will be instructed to self-report concerns either via an online event reporting system, by calling the site, or by calling a 24-hour hotline. Participants will have access to event reporting via the online system from the signing of the informed consent form (ICF) until the Final Visit (Day 90, Day 120, or Day 180 depending on the time of consent).

Events of special interest (ESIs) and SAEs will be extracted by site personnel from the participant's medical record if the participant seeks medical care or if hospitalization occurs, each of which notifies the site to conduct follow up.

Medical occurrences that begin before the start of study drug/placebo, but after obtaining informed consent, will not be considered an AE.

Non-serious AEs (or ADEs, as applicable) may be reported by the participant, but will not be further assessed by the site or study personnel unless the event meets the criteria of an ESI.

Events of Special Interest (ESIs), SAEs, and UADEs (as applicable) will be collected from the start of study drug/device combination until the Final Visit (Day 90, Day 120, or Day 180 depending on the time of consent) or until 30 days after the last dose/use of device if participant terminates the study early.

9.1.3. Assessing Causality of a Serious Adverse Event

If an SAE occurs, the site investigator or medical monitor will assess the relationship to study drug by using the following criteria:

- Related:
  - Study drug – there is a temporal relationship between study drug and event onset or the event abates when study drug is discontinued or known to occur with study drug.
  - Device – an event is due to the use of the device and cannot be reasonably explained by an alternative cause.
- Not related: The event has no temporal relationship to study drug (or study device, as applicable) or the AE (or ADE, as applicable) has a much more likely alternate etiology or is due to an underlying or concurrent illness or effect of another drug (or device, as applicable).

9.1.4. Reporting and Monitoring of SAEs

All of the study drugs used in this platform protocol are repurposed medications that are approved for marketing in the US for another medical condition. However, their investigational use for treatment of COVID-19 infection is not an approved indication and will be under an IND and subject to IND regulations in 21 CFR 312. The IND sponsor or designee will review SAEs weekly, and will perform aggregate reviews of SAEs every two weeks. The IND sponsor or her designee will be responsible for determining if the safety reporting criteria are met per 21 CFR 312.32(c)(1)(i)(C) and 21 CFR 312.32(c)(1)(iv) and will notify the Data Coordinating Center (DCC) to prepare an aggregate report for submission to the FDA. An aggregate safety report will

be submitted to FDA as soon as possible, but in no case later than 15 calendar days after the IND sponsor determination. If the IND sponsor determines that an unexpected fatal or life-threatening suspected adverse reaction occurs markedly more frequently in a study drug arm than in the placebo arm, an aggregate safety report will be submitted to the FDA as soon as possible, but in no case later than 7 calendar days after the IND sponsor determination. Information on individual SAEs will be available upon request from the Agency following the submission of any aggregate reports.

Any UADE(s) that the IND sponsor determines is/are reportable will be submitted to the FDA, manufacturer, all reviewing IRBs, and all participating investigators within 10 working days of when the sponsor makes that determination.

If the IND sponsor determines that a UADE presents an unreasonable risk to participants, all investigations or parts of investigations presenting that risk shall be terminated as soon as possible. Termination shall occur not later than 5 working days after the sponsor makes this determination and no later than 15 working days after the sponsor first received notice of the effect.

All hospitalization and death events will be adjudicated (see Section 10.9), any event that is determined to be COVID-19-related will **not** be reportable as an expedited SAE, with the exception of events that are related to study drug and unexpected, which will be reportable regardless of relatedness to COVID-19. All events that are **not** COVID-19-related per the adjudication process will be reviewed by the DCRI Safety Medical Monitor to determine if the event is a reportable SAE.

Individual SAEs and UADEs must be entered into the data system within 24 hours of site awareness. The DCRI Safety Surveillance team will notify pharmaceutical partners of SAEs within 1 business day of their receipt that occur involving the specific appendix of the supplied study drug/placebo, as required. Serious Adverse Events that are related and confirmed unlisted by the DCRI Safety Medical Monitor will be reported to the FDA as SUSARs; as 7-day reports for unexpected fatal or life-threatening adverse reactions and 15-day reports for serious and unexpected adverse reactions. If the IND sponsor, IDMC, or FDA note a clinically important increase in the rate of a SUSAR, the IND sponsor or her designee will notify investigators no later than 15 calendar days after determining that the information qualifies for reporting. The investigator will follow all reportable events until resolution, stabilization or the event is otherwise explained. The DCRI Safety Surveillance Team will follow all SAEs until resolution, stabilization, until otherwise explained.

Pregnancies that occur while on-study will be collected and will not be followed to outcome if outcome occurs beyond the participant’s Final Study Visit, however, any associated ESI or SAE should be reported if information can be collected and entered into the EDC. The DCRI Safety Surveillance team will notify pharmaceutical partners of a pregnancy within 1 business day of receipt that occur involving the specific appendix of the supplied study drug/placebo, as required.

9.1.5. Events of Special Interest

The following are also considered ESIs to the study and will be collected by study personnel via medical record review when concern for ESIs are observed for hospitalized participants:

- Hypoxia, defined as two consecutive pulse oximetry readings ≤ 93%

Each study drug may have a unique list of possible related ESIs. Refer to the relevant appendices.

9.2. Unanticipated Problem (UP) and Terminations

9.2.1. Definition of Unanticipated Problem

The OHRP considers UPs involving risks to participants or others to include, in general, any incident, experience, or outcome that meets all of the following criteria:

- Unexpected in terms of nature, severity, or frequency given (a) the research procedures that are described in the protocol-related documents, such as the IRB-approved research protocol and informed consent document; and (b) the characteristics of the participant population being studied.
- Related or possibly related to participation in the research ("possibly related" means there is a reasonable possibility that the incident, experience, or outcome may have been caused by the procedures involved in the research).
- Suggests that the research places participants or others at a greater risk of harm (including physical, psychological, economic, or social harm) than was previously known or recognized.

9.2.2. Reporting of an Unanticipated Problem

The site investigator will report UPs for their participants to the DCC. The site may also be required to inform their reviewing IRB about a UP occurring at the local institution. The UP report to the DCC will include the following:

- A detailed description of the event, incident, experience, or outcome
- An explanation of the basis for determining that the event, incident, experience, or outcome represents an UP
- A description of any changes to the protocol or other corrective actions that have been taken or are proposed in response to the UP
- The DCC will document and review all UPs. Details of the UP reporting process will be located in the MOP.

10. Statistical Considerations

10.1. Statistical Hypotheses

10.1.1. Primary Hypothesis

The primary hypothesis in this trial is that participants who receive study drug will have reduced disease progression to hospitalization or death and/or more rapid resolution of symptoms as compared to those who receive placebo.

10.2. Sample Size Determination

This study is designed to be analyzed using a Bayesian approach, accepting the possibility of adding and dropping of arms as the trial progresses. There is also the potential for extending accrual in a study drug appendix if there is the potential to demonstrate benefit. Detailed simulations will be used to demonstrate the operating characteristics common to each study drug appendix. Decision thresholds will be set to balance overall power with control of the Type I error rate in the context of the appendix-specific goal.

To aid planning for this trial, symptom count and clinical event data were estimated from participants in a clinical trial with similar inclusion criteria. Data were not collected daily in that study, but evaluations were completed on Day 10 after randomization, which is considered a clinically meaningful point in time. Based on the observed distribution, it is estimated that studies of about n=600 (300 study drug and 300 placebo) will be sufficiently sized to determine whether there is evidence of meaningful benefit with > 85% power (**Table 2**). Moreover, when a study drug demonstrates overall effectiveness, the planned adaptations to increase targeted accrual for the purpose of demonstrating benefit on clinical events is a reasonable extension within the context of this platform. The final decision thresholds and operating characteristics selected for each appendix, if deviating from the common approach described in the SAP, will be customized in an appendix-specific SAP. It is expected that this study will enroll up to 15,000 adults, depending on the number of study drug appendices that are added and adjustments to sample size depending on the data.[21]

Table 2: ACTIV-6 Sample Size Estimates and Power

		Power				Power		
**OR**	**Corresponding difference in mean symptom burden**	**80%**	**85%**	**90%**	**Corresponding Risk Difference in clinical events**	**80%**	**85%**	**90%**
0.4	2.10	75	86	101	0.025	343	392	459
0.5	1.98	132	150	176	0.021	455	520	608
0.6	1.86	242	277	324	0.017	675	772	903
0.7	1.74	496	567	664	0.012	1004	1148	1343
0.8	1.61	1267	1449	1696	0.009	1652	1889	2211

The sample sizes given are the sample size for the study drug arm only. Placebos will be borrowed across study drug appendices. The total size of the placebo arm will be equal to the size of the study drug arm. The total number of placebos in the trial will depend on eligibility of participants among the study drug appendices and the number of study drugs. The calculations are based on symptom burden, hospitalization, and death at Day 10.

10.3. Randomization

See Section 5.3.3 for additional details.

10.4. Blinding

The investigators, treating clinicians, and study participants will all remain blinded to study drug versus placebo assignment until after the database is locked and blinded analysis is completed. Only the Investigational Drug Service (IDS) and staff who are handling randomization codes and unblinded members of the biostatistical team who are preparing closed IDMC interim reports will be unblinded. The statistical staff responsible for preparing IDMC reports will not directly interact with the clinical team that delivers care to the study participants. Specifically, study medication will be dispensed with packaging and labelling that would blind treatment assignment. Unblinding will occur only if required for participant safety or treatment at the request of the treating clinician.

The web-based randomization system will include blind-breaking instructions. Participant safety must always be the first consideration in making an unblinding determination. If the investigator decides that unblinding is warranted, the investigator should complete an unblinding request, which will immediately notify the Medical Monitor (see MOP for details).

10.5. Populations for Analyses

Modified Intention to Treat (mITT) Population:

- All participants who receive study drug/placebo.
- Participants who do not receive study drug, for any reason, will be excluded; while this modifies the intention to treat principle, the failure of delivery of medications from site to participant is not under the control of either investigator nor participants and is expected to occur infrequently and randomly. Similarly, early death of a participant or withdrawal prior to the study drug being received is possible, but unlikely and expected to occur randomly between study drug appendices. All other participants will be included, and they will be analyzed according to which arm they were assigned. Thus, the mITT analysis set includes all participants who were randomized and received the study drug.

Safety Population:

- The safety population will include those persons in the mITT population who report taking at least one dose of study drug or matching placebo. In the unlikely case a participant receives the incorrect study drug, participants will be grouped according to the treatments that they received.

10.6. Statistical Analyses

The main trial SAP will be finalized prior to the primary analysis. It will include a description of the statistical analyses and detailed simulations used to inform the sample size estimates. Appendix-specific decisions, such as choice of covariates for the model, and context specific decisions such as deviations in decision making thresholds or in targeted accrual, will be made blinded to data and prior to analyses. Such decisions will be documented in the trial master file.

This section is a brief summary of the planned approach to statistical analyses of the most important endpoints including primary and secondary endpoints.

10.6.1. General Considerations

Baseline demographic and clinical variables will be summarized for each randomized arm of the study. Descriptive summaries of the distribution of continuous variables will be presented in terms of percentiles (e.g., median, 25th and 75th percentiles) along with means and standard deviations. Categorical variables will be summarized in terms of frequencies and percentages. Histograms and boxplots may be used to visualize the data.

If an efficacy signal is observed at n=300 or n=600 following sIA, the trial will enter assessment of the primary objective. At that time, the pre-determined primary clinical endpoint for the appendix will be evaluated as either clinical events (hospitalization or death) or time to recovery. The unselected endpoint will be reported as part of the secondary objective. Both clinical endpoints will be analyzed using a covariate-adjusted statistical model.

10.6.2. Statistical modeling

Estimation and inferences about the effect of each study drug versus matching placebo will be made using Bayesian regression methods. For each study drug, the matching placebo arm will consist of concurrently randomized participants that meet the inclusion and exclusion criteria for that study drug. The statistical models are described in detail in the SAP. Briefly, a longitudinal ordinal regression model will be used for the sIA, a logistic regression model will be used for clinical events, and proportional hazards models will be used for time to event analyses. All models will be adjusted for covariates, including baseline symptom severity and time from symptom onset. Covariates will be formally specified prior to an analysis, taking into account emerging data and changing context.

10.6.3. Assessing Effectiveness (Primary Objective)

The overall effect of each study drug versus matching placebo will be quantified using one of the following two primary endpoints, which will be defined and documented per study drug appendix prior to the initial IA: clinical events (hospitalization or death) or time to recovery.

The primary analysis will be implemented separately for each study drug appendix where the matching placebo arm will consist of concurrently randomized participants that meet the inclusion and exclusion criteria for that study drug. Decision thresholds, priors, and meaningful effect sizes may change during the course of the pandemic, as vaccination rates, case rates, and new therapies continue to evolve. Thresholds, effect sizes and priors, that vary during the trial from those described in the main SAP will be documented in the trial master file or other designated document. Prior to any interim or final analysis, all decision thresholds, priors and effect sizes will be confirmed and evaluated using extensive simulations to demonstrate the overall Type I error rate remains below 0.05. An mITT approach will be used for primary analyses. All available data will be used to compare each study drug versus placebo control, regardless of post-randomization adherence to study protocols.

10.6.4. Interim Analyses (IA), Early Stopping, and Type-I Error Control

Individual study drugs may require different sample sizes, and the sample sizes may be adjusted based on the results of IA. Therefore, fixed enrollment triggers will be used for IA. An IA will occur after enrollment and completion of 14 day follow-up of approximately every 300

participants in a study arm (150 in study drug arm and 150 in placebo arm). Study drug appendices may be stopped early for efficacy or futility (see **Figure 2**). Thresholds are described in the SAP to guide stopping of each appendix if there is clear evidence of benefit or if there is sufficient evidence to declare futility.

The following schedule and decision thresholds will be followed for IAs:

i) Screening IA (n=300):
   a. The study drug is found to have benefit (efficacy). Study drug appendix will proceed to primary objective IA at n=300. *Note: this is also a check for harm as all assessments are two-tailed.*
   b. The study drug is not found to have benefit, enrollment continues in the study drug appendix and sIA is repeated at n=600.

ii) Screening IA (n=600):
   a. It would be futile to attempt to show a benefit of the study drug based on the predicted probability of success (PPOS) and other factors. The study drug appendix will be terminated.
   b. Futility is not determined. Study drug appendix will proceed to primary objective IA at n=600.

iii) Primary Objective IA (n=300): if the criteria for proceeding to the primary objective are met when n=300, a primary objective IA will be conducted. The following decisions will be assessed:
   a. The study drug is found to have benefit (efficacy), the study drug appendix will be terminated as the primary endpoint has been met.
   b. It would be futile to attempt to show a benefit of the study drug within the PPOS and other factors. The study drug appendix will be terminated.
   c. Efficacy/futility is undeterminable, enrollment will continue in the study drug appendix and the primary objective IA will be assessed at n=600.

iv) Primary Objective IA (n=600, 900): if the criteria for proceeding to the primary objective IA are met when n=600 or n=900, a primary objective IA will be conducted. The following decisions will be assessed:
   a. The study drug is found to have benefit (efficacy), the study drug appendix will be terminated as the primary endpoint has been met.
   b. It would be futile to attempt to show a benefit of the study drug based on the PPOS and other factors. The study drug appendix will be terminated.
   c. Efficacy/futility is undeterminable, enrollment will continue in the study drug appendix and the primary objective will be assessed after another 300 participants have been enrolled, or until n=1200.

The analysis for the sIA will use a covariate adjusted statistical model. The outcome is an ordinal variable, which is the overall symptom burden measured on a none / mild / moderate / severe scale with hospitalization and death added as the 5th and 6th level of the ordinal scale. The outcome is measured daily for 14 days. The outcome is compared between participants receiving study drug and participants receiving placebo each of the 14 days using a longitudinal statistical model that takes into account the repeated measurements on each participant. The statistical

model can be used to estimate the days of benefit – the number of days for which being on an active study drug results in a better outcome than being on a comparator. Days benefit, restated in terms of concordance and discordance probabilities, is the difference between (a) the probability that the intervention is better and (b) the probability that the non-intervention is better, summed over all the days of follow-up. This is the main quantity, or estimand, that will be used to make early go/no-go decisions for each appendix.

The primary objective IA will follow processes described in Section 10.6.3 for assessment of efficacy. The primary endpoint used for the primary objective IA will be selected per appendix prior to the initial IA.

A posterior probability of meaningful benefit for a study drug in comparison to the placebo control of greater than the specified threshold will result in a declaration of overall superiority. A PPOS when n=1200 is less than the specified threshold will result in a declaration of futility.

Futility is a low probability of achieving any conclusions within a reasonable time frame or within the context of the trial. Prior to each IA, the target date for study completion will be specified, and accrual will be projected by that target date. A statistical model may be used to predict accrual. Futility assessment will use the lowest of either the planned accrual or predicted accrual at study closure.

The combination of decision thresholds and effect sizes have been selected to balance the ability to observe a meaningful effect on symptoms, to observe the potential for an effect on clinical outcomes, and to maximize power while controlling the Type 1 error rate. For each appendix, decision making thresholds will be set to achieve appendix-specific goals and simulations will be used to demonstrate that the operating characteristics are consistent with a Type I error control of at least 5%, as described in the SAP.

10.6.5. Sensitivity and Supplementary Analyses

The sensitivity analyses described in the SAP are designed to test robustness of the results to assumptions in the statistical models. In addition to checking assumptions about the modeling approach, association of adherence with outcomes will also be ascertained. In the main statistical model, the number of doses of study drug consumed will be added as a covariate.

10.6.6. Differential Treatment Effects and Subgroup Analyses

Differential treatment effect, also referred to as heterogeneity of treatment effect, refers to differences in treatment efficacy as a function of pre-existing participant characteristics such as baseline variables. This is often assessed by forming subgroups. However, these subgroups do not inherit the baseline covariate adjustment of the full participant outcome model, and are problematic because of improper subgrouping when a continuous variable is used. For example, dichotomizing age at 65 years is arbitrary and it is very unlikely that any study drug effect has a discontinuity in effect at 65 years old. Also, subgroup estimates and statistical assessments of them are unreliable and are often taken out of context when a more systematic analysis does not find evidence for an interaction between the covariate and study drug.

For these reasons, analysis of differential study drug effect will be prespecified and model based. For example, effectiveness variability can be estimated with continuous age by adding a smooth age by study drug interaction into the model and using this model and using this model to

estimate treatment contrast and their uncertainties across age = 10, 11, …, 100. Differential treatment effects by sex, body mass index, and age will be examined. Prior to the final analysis, additional important subgrouping variables will be defined and listed in the SAP. Knowledge about concomitant therapies, risk factors, and vaccinations are expected to continue to evolve and inform the final decision on their inclusion in these analyses.

Studies under this master protocol will be sized only for assessing overall study drug effects. Thus, there may be inadequate power to (1) examine interactions and to (2) estimate covariate-specific treatment effects (e.g., odds ratio at age 70 or for females).

10.6.7. Secondary Clinical Endpoint

The COVID Clinical Progression Scale score on Day 14 will be compared between participants in each study drug arm and the placebo arm using a covariate adjusted proportional odds model. A similar approach will be used for QOL outcomes. Covariates will be prespecified and will include at a minimum: age, baseline severity, and duration of illness. The proportional odds assumption will mainly be examined using graphical methods—e.g., the logit of the empirical cumulative distribution function of the ordinal scale should be parallel among categories of covariates. If proportionality is clearly violated, a partial proportional odds or non-proportional odds models will be considered. As before, a Bayesian approach to model interpretation will be used. For estimating time to symptom resolution based on the definition of at least three consecutive days without symptoms, a proportional hazards model will be used. Since death is a competing risk, cause-specific hazards will be estimated. Observations will be censored at 28 days.

10.6.8. Exploratory Analysis

Exploratory analyses involve the same outcome variables, measured at 90, 120, or 180 days. Exploratory analysis will focus on describing long term outcomes, particularly symptoms and severity, clinical status, and QOL. Statistical models will use a similar form as for the main analysis. As well as simple analysis that consider the effect of treatment on long term outcomes, the SAP will describe how participant state during the intervention period will be used to inform longer term outcomes.

10.6.9. Adherence and Retention Analysis

Withdrawals from study drug and consent withdrawals will be tracked via the online system. Participants will be asked about their use of study drug. Those reporting discontinuation or switching will be asked about the reasons for discontinuation/switching.

Measures of study retention to inform follow-up time will be based on several measures, including web-based check-ins for symptoms and COVID-19 outcome reporting.

10.7. Interim Reporting

In addition to routine evaluation of decision thresholds pursuant to the statistical design of this study, regular IDMC reviews will be conducted to ensure the safety of study participants. Regular IDMC meetings will monitor the following parameters at a minimum:

- Recruitment progress

- Enrollment overall and by subgroups
- Adherence, retention, and status of data collection
- Events of special interest (ESIs)
- Unanticipated problems
- Serious adverse events (SAEs)

Interim examination of clinical endpoints will be based on the accrual of primary endpoint data. It is expected that reviews of the data will occur approximately after each 300 participants are enrolled in each study drug appendix (150 in study drug arm and 150 in placebo arm).

For ethical reasons, interim examinations of key safety and process data will be performed at regular intervals during the course of the trial. The DCC will create reports to track participant enrollment, rates of adherence with the assigned treatment strategy, and frequency of protocol violations. Prior to each meeting, the DCC will conduct any requested statistical analyses and prepare a summary report along with the following information: participant enrollment reports, rates of adherence with the assigned treatment, and description of SAEs.

Safety reports will be prepared for the IDMC approximately weekly once enrollment begins. The prespecified stopping thresholds are intended to guide the interpretation of interim analyses and are not a strict rule for early termination. It is expected that both internal and external factors will influence the decisions of the IDMC. The SAP will describe the planned interim analyses and futility monitoring in detail.

10.8. Independent Data Monitoring Committee (IDMC)

The IDMC will monitor participant safety and study performance. An IDMC charter that outlines the operating guidelines for the committee and the procedures for the interim evaluations of study data will be developed and agreed upon by the IDMC. Reports will be prepared by the DCC in accordance with the plan outlined in the charter, or as requested by the IDMC chair, and will include interim analyses of primary and secondary endpoints, additional safety events, and other information as requested by the committee. After each scheduled closed meeting, the IDMC will send a recommendation to the IND sponsor to continue, modify, or terminate the study. After approval, the recommendations will be forwarded by the clinical coordinating center (CCC) to investigators for submission to their local, regional and national IRB/Ethic Committees, as applicable. Please refer to the IDMC Charter for further details.

10.9. Adjudication Committee

The medical records will be requested for all participants reporting a hospitalization and/or death at any point during the study. For each participant-reported hospitalization or death event, the DCRI Clinical Event Ascertainment (CEA) group will review the medical records and confirm the occurrence and root cause of the event as part of an adjudication process. The CEA group includes specialists relevant to the hospitalization or death events of interest, additional details about review procedures will be provided in an adjudication charter.

11. Ethical Standards

11.1. Institutional Review Board (IRB)

The protocol, ICF(s), recruitment materials, and all participant materials will be submitted to the IRB(s) of record for review and approval. This approval must be obtained before any participant is enrolled. Any amendment to the protocol will require review and approval by the IRB(s) before being implemented in the study. All changes to the consent form will also be IRB-approved and a determination will be made regarding whether previously consented participants need to be re-consented.

11.2. Informed Consent Process

All consenting will occur either via an electronic consent process or a paper process. Consent forms describing in detail the study drug/placebo, study procedures, and risks will be given to the participant and documentation of informed consent is required prior to starting study procedures. Informed consent is a process that is initiated prior to the individual's agreement to participate in the study and continues throughout the individual's study participation. A description of risks and possible benefits of participation will be provided to the participants. A description of the current available therapies as part of usual care outside of this trial will be provided to the participants and clarification that receipt of such therapies are not part of exclusion criteria will also occur. Consent forms will be IRB-approved and the participant will be asked to read and review the document. The participant will be provided a phone number and email in the event they have questions about study participation. This will allow them to communicate with the investigators (or their delegate), for further explanation of the research study and to answer any questions that may arise, as necessary. Participants will have the opportunity to carefully review the consent form and ask questions prior to signing.

The participants should have the opportunity to discuss the study and think about it prior to agreeing to participate. The participant will sign the informed consent document prior to any procedures being done specifically for the study. The participants may withdraw consent at any time throughout the course of the study. A copy of the informed consent document will be provided to the participants for their records. The rights and welfare of the participants will be protected by emphasizing to them that the quality of their medical care will not be adversely affected if they decline to participate in this study.

The study team will distinguish between the desire to discontinue study drug and the desire to withdraw consent for study follow-up. In the event that a participant withdraws consent, the investigator or his/her designee will clarify with the participant and document whether the withdrawal is temporary or permanent, and if a full or partial withdrawal.

11.3. Participant and Data Confidentiality

Participant confidentiality is strictly held in trust by the participating investigators, their staff, and the sponsor(s) and their agents. This confidentiality is extended to cover testing of biological samples in addition to the clinical and private information relating to participants. Therefore, the study protocol, documentation, data, and all other information generated will be held in strict confidence. No information concerning the study or the data will be released to any unauthorized

third party without prior written approval of the sponsor. The study participant's contact information will be securely stored in the clinical study database.

Study participant research data, which is for purposes of statistical analysis and scientific reporting, will be transmitted to and stored at the DCC. The study data entry and study management systems used by clinical sites and by research staff will be secured and password protected. At the end of the study, all study-related data storage systems will be archived according to local processes.

11.4. Site Management and Quality Assurance

The study team will work in tandem to ensure that the data collected in this study are as complete and correct as possible. A four-step, multi-functional approach to quality control will be implemented:

- Training: Prior to the start of enrollment, the clinician investigators and key study personnel at each site will be trained with the clinical protocol and data collection procedures, including how to use the Electronic Data Capture (EDC) system. Follow-up training and training for new study personnel or new versions of the protocol will be conducted as needed.
- Monitoring: The CCC, along with the DCC, will ensure that data collection is handled properly, will provide in-service training, and will address questions from site investigators and coordinators. Electronic review of data quality and completeness will occur on a regular and ongoing basis. Any issues will be addressed. At a minimum, source document verification will occur, as needed, for confirmation of COVID-19 diagnosis and hospitalization(s).
- Managing data: After the data have been transferred for statistical summarization, data description, and data analysis, further crosschecking of the data will be performed with discrepant observations being flagged and appropriately resolved through a data query system.
- Reviewing data: Data regarding events of interest will be reviewed to ensure appropriate documents are collected for IDMC review. The DCC will monitor standardized classification of symptoms and contact site study teams when events comprising the primary endpoint are not complete.

11.5. Site Monitoring

This study will employ a centralized risk-based approach to monitoring with routine and periodic review of participant-submitted data to validate the informed consent process, select eligibility criteria, hospitalization, identify and follow-up on missing data, inconsistent data, data outliers, etc. and ensure completion of administrative and regulatory processes. The study team will facilitate regular communication through training sessions, teleconferences, videoconferencing, email, etc. Using quality-by-design principles, steps will be taken at the study design stage to foresee and limit problems that might occur during the study conduct. Follow-up from the online system and call center is expected to keep participants engaged. Minimal levels of intervention and a focus on observing rather than influencing the study participants greatly increases the likelihood that Good Clinical Practices will be followed. Central statistical monitoring is

particularly useful for identifying unusual patterns in data. An integrated approach to quality surveillance will be deployed, which will be detailed in the appropriate study management plans.

12. Data Handling and Record Keeping

12.1. Data Collection and Management Responsibilities

Minimizing research activities and conducting the trial in a pragmatic manner will increase the ability to complete the trial in the face of strained clinical and research resources during the COVID-19 pandemic. Data will be collected by electronic methods, supplemented by telephone or videophone follow-up and from the electronic health record.

Data will be collected directly from participants using REDCap through text messaging or email with a survey link, or phone call as back up. The process for using text messaging is Health Insurance Portability and Accountability Act (HIPAA) compliant.

Site personnel or participants will enter study data into a secure online database. Data will be maintained in a secure online database until the time of study publication. At the time of publication, the DCC will generate a de-identified version of the database for archiving (see Section 12.4).

12.2. Study Records Retention

Study documents should be retained for a minimum of six years after the study has ended. However, if required by local regulations, these documents should be retained for a longer period. No records will be destroyed without the written consent of the sponsor, if applicable. It is the responsibility of the sponsor to inform the investigator when these documents no longer need to be retained.

12.3. Protocol Deviations

A protocol deviation is defined as non-compliance with the clinical study protocol, GCP, or MOP requirements. The non-compliance may be on the part of the participant, site investigator, or the site staff.

A major protocol deviation is a significant divergence from the protocol that may have significant effect on the participant's safety, rights, or welfare and/or on the integrity of the study data. Major protocol deviations must be sent to the study IRB and local IRB per their guidelines, recorded in source documents, and reported to the coordinating center. Major protocol deviations will be tracked. For this study, any missed or delayed survey completion will not be considered a major protocol deviation. Refer to the MOP for details.

12.4. Publication and Data Sharing Policy

This study will comply with the NIH Public Access Policy, which ensures that the public has access to the results of NIH-funded research. Methods of data sharing will include 1) archiving de-identified data in a data repository and 2) sharing of limited datasets under a Data Use Agreement (DUA) and IRB approval. Data will be made available to qualified investigators by archiving a fully de-identified dataset in a platform to be determined at the end of the trial. Both repositories allow users to search, view study information, and then submit an application to receive data. Prior to archiving study data, the DCC will produce a final dataset that will be stripped of all personal health information (PHI) in compliance with the HIPAA privacy rule.

The relative timing of an event will be retained in the dataset converting to study days instead of dates.

The study result will be returned, including some participant specific results, to enhance value from participation. Study results will be disseminated to the public and the medical community through presentations at scientific meetings and publishing manuscripts in high impact peer-reviewed journals. The International Committee of Medical Journal Editors (ICMJE) member journals have adopted a clinical studies registration policy as a condition for publication. The ICMJE defines a clinical study as any research project that prospectively assigns human participants to intervention or concurrent comparison or control groups to study the cause-and-effect relationship between a medical intervention and a health outcome. The ICMJE policy, and the Section 801 of the Food and Drug Administration Amendments Act of 2007, requires that all clinical studies be registered in a public registry such as ClinicalTrials.gov, which is sponsored by the National Library of Medicine. For interventional clinical trials performed under NIH IC grants and cooperative agreements, it is the grantee's responsibility to register the study in an acceptable registry, so the research results may be considered for publication in ICMJE member journals.

13. Study Leadership

The Steering Committee is a multi-stakeholder committee that oversees the study and includes representatives from clinical sites, the trial coordinating center, the NIH, PCORI, Operation Warp Speed, the FDA, National Center for Advancing Translational Sciences (NCATS), ACTIV representatives with no conflict of interest, and academic and industry advocates.

The CCC and DCC are each overseen by PI(s). The CCC is responsible for study coordination, site management, communication, and financial administration. The DCC is responsible for treatment allocations, receipt and processing of data, quality control programs, and statistical analysis and reporting.

An independent IDMC will oversee the safety and welfare of trial participants as well as provide recommendations for continuation, discontinuation or revision of the trial.

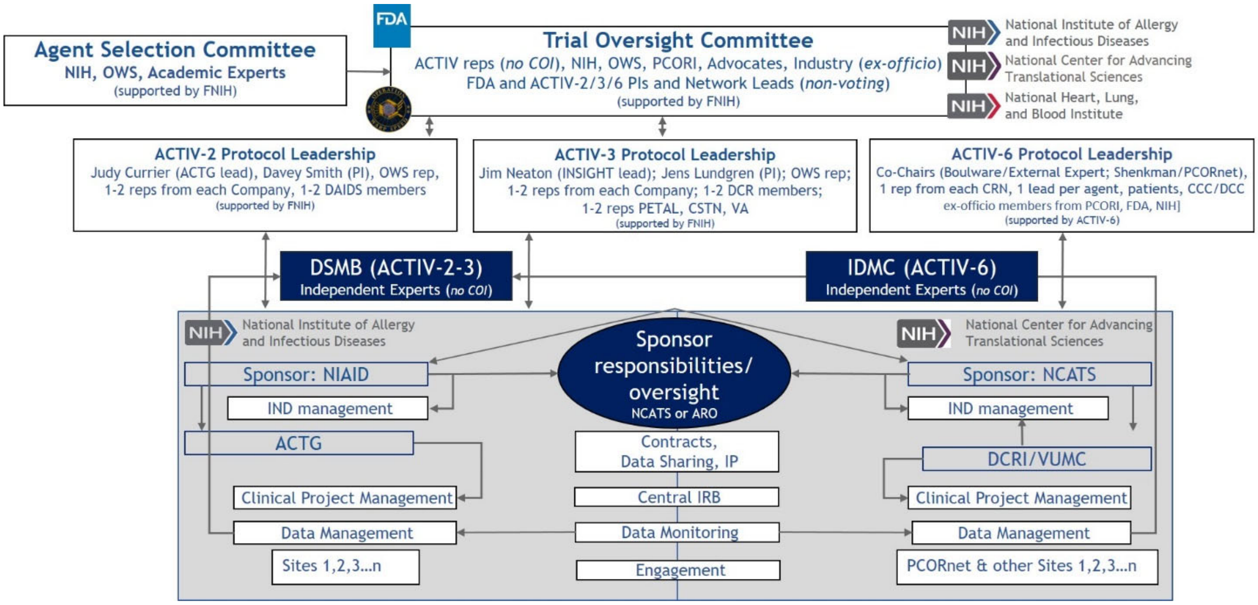

Figure 3: Operational Structure Diagram

14. Summary of Changes

Protocol Version (version #, date)	Summary of Changes
Version 1.0, 01APR2021	N/A, Version 1.0
Version 2.0, 25MAY2021	• Added current use of study drug or study drug/device combination as an exclusion criteria (Sections 1.1 and 5.2);<br>• Added a phone call follow-up the day after first study drug dose, clarified that follow-up will occur if two consecutive days of reporting are missed during Days 3-14, and added that sites/call center will collect missing survey information during follow-up calls (Section 7.3);<br>• Added at-home pulse oximetry readings and description of at-home pulse oximetry reading collection (**Table 1** and Section 8.2);<br>• Added symptom severity scale (Section 8.2);<br>• Added Adverse Device Effect and Unanticipated Adverse Device Effects definitions, collection/reporting period details, and causality assessment details (Sections 9.1.1, 9.1.2, 9.1.3, 9.1.4);<br>• Clarified that hypoxia ESI will only be collected from hospitalized participants (Section 9.1.5);<br>• Clarified that any missing or delayed survey completion will not be considered a major protocol deviation (Section 12.3);<br>• Updated Ivermectin and matched placebo information and packaging (Sections 16.3, 16.3.1, 16.3.3, 16.4.1);<br>• Added Appendix B – Fluvoxamine maleate (Section 17);<br>• Added Appendix C – Fluticasone Furoate (Section 18);

	• Changed symptom freedom to symptom resolution for consistency throughout;<br>• Other administrative changes throughout.
Version 3.0, 06JUL2021	• Clarified that the sample size increase will included 1:1 active study drug to placebo (Section 1.1);<br>• Added footnote 6 to COVID-19 Outcomes during Intervention Period to clarify that these will be assessed on Day 7 and 14 (**Table 1**);<br>• Added that events that are COVID-19 related AND study drug related and unexpected will be considered reportable (Section 9.1.4);<br>• Updates made to fluvoxamine appendix: excluded linezolid, use of fluoxetine within 45 days of consent, and bipolar disorder per FDA feedback (Section 17.2); added precautions of additional drugs including tramadol, buspirone, fentanyl, lithium, amphetamines, St. John's Wart, carbamazepine, quinidine, and tacrine per FDA feedback (Section 17.2.1);<br>• Updates made to fluticasone furoate appendix: brand name Arnuity Ellipta replaced with fluticasone furoate throughout (Section 18); changed liver failure exclusion criteria to "moderate to severe hepatic impairment, defined as Child-Pugh B or C" (Section 18.2); removed hepatic impairment precautions as it was added to exclusion criteria (Section 18.2.1);<br>• Other minor administrative changes throughout.
Version 4.0, 20DEC2021	• Removed protocol number as no protocol number will be assigned;

	• Changed primary objective from symptom reduction to effectiveness based on clinical outcome endpoints of hospitalization/death or time to recovery (Sections 1.1 and 3);<br>• Removed unnecessary "e.g., hospitalization and death" from first secondary objective (Sections 1.1 and 3);<br>• Added rationale for sIA (Sections 1.1 and 4);<br>• Inclusion criterion #3: added reinfection (Sections 1.1 and 5.1);<br>• Exclusion criteria updates: Removed "prior diagnosis of COVID-19 infection (>10 days from screening)" as it was causing confusion for sites and is already covered by Inclusion criterion #3; specified that current or recent hospitalization for COVID-19 infection is exclusionary, not all hospitalizations; added a time window for current or recent use of study drug or combination for within the last 14 days; added "current or planned participation in another interventional trial to treat COVID-19, at the discretion of the study PI" (Sections 1.1 and 5.2);<br>• Sample size considerations updated to include sIA (Sections 1.1 and 10.2);<br>• Statistical considerations for Primary Analysis updated to align with change in primary objective and IAs (Sections 1.1, 10.6.1, and 10.6.3);<br>• Interim Analysis updated to specify that IA will occur ~ every 300 participants instead of every 200, included sIA, and primary objective IA (Sections 1.1, 10.6.4, and 10.7);<br>• Added **Figure 2** to portray IA process (Section 1.2);

	• Updated study background and rationale based on new data (Sections 2.1 and 2.2);<br>• Updated secondary outcome measures and reported endpoints (Section 3);<br>• Added additional information regarding arm eligibility depending on the number of arms open to provide clarity for sites (Section 4.1);<br>• Added that screen failures also include participants who consent, then on review by the site, are found to be ineligible for the study (Section 5.4);<br>• Added additional reasons why participant may not receive study drug/placebo and specified that these participants would be identified as randomized not enrolled, instead of consented not enrolled (Section 5.5);<br>• Specified that participants will be contacted directly if the miss one daily symptom reporting during Days 3 to 14 (Sections 7.3 and 9.1.2);<br>• Clarified that all participants will be asked to complete symptom reporting on Days 21 and 28, regardless of symptom resolution (**Table 1**, Section 8.1.3);<br>• Added that "overall symptom burden" is collected as part of Symptom Reporting (Section 8.2);<br>• Updated primary hypothesis to align with updated primary objective (Section 10.1.1);<br>• Model priors section removed and Statistical modeling simplified to refer to detailed description in SAP as models will be adjusted per appendix (Section 10.6.2);<br>• Removed details of sensitivity analysis and referred to SAP (Section 10.6.5);

	• Clarified that additional subgrouping variables may be added to the SAP prior to final analysis (Section 10.6.6);<br>• Changed Appendix A – Ivermectin title to Ivermectin 400 (Section 16); removed CYP3A4 and P-gp precautions as not noted in the IB for the ivermectin study drug (Section 16.2.1); in Section 16.2, deleted "use of warfarin, CYP3A4, P-gp inhibitor drugs, or CYP3A4 substrates" appendix-level exclusion criteria and added "Current or planned use of the following drugs:<br>○ Antiarrhythmic/antihypertensive drug class: quinidine, amiodarone, diltiazem, spironolactone, verapamil<br>○ Antibiotic-macrolides drug class: clarithromycin, erythromycin<br>○ Antifungal drug class: itraconazole, ketoconazole<br>○ Immunosuppresant drug class: cyclosporine, tacrolimus<br>○ Anti-HIV drug class: indinavir, ritonavir";<br>• Added Appendix D – Ivermectin 600 (Section 19);<br>• Other administrative changes throughout.
Version 5.0, 17MAR2022	• Added illness severity objective (Sections 1.1 and 3);<br>• Removed reference to appendix-specific SAP, only significant deviations from the main SAP analysis will require appendix-specific SAPs. Choice of primary objective, covariates, etc. will be documented outside of the SAP (Sections 1.1, 10.2, 10.6, 10.6.3, 10.6.4);

	• Clarified that primary objective occurs at 28 days (Sections 1.1 and 3);<br>• Updated COVID-19 status, vaccination, and treatment options background (Sections 1.1 and 2.1);<br>• Defined "receipt of study drug" (Section 5.5);<br>• COVID-19 vaccination added to abbreviated medical history (Section 8.2);<br>• Updated instructions for unblinding (Section 10.4);<br>• Specified that covariates will take into account emerging data and changing context (Section 10.6.2);<br>• Added that this study operates in addition to usual care in additional protocol locations (Sections 11.2 and 2.1);<br>• Appendix A closed to enrollment on 04FEB2022;<br>• Appendix C closed to enrollment on 08FEB2022;<br>• Added Appendix E – Combination Fluvoxamine Maleate and Fluticasone Furoate;<br>• Added Appendix F – Montelukast (Section 21);<br>• Other administrative changes throughout.
Version 6.0, 17JUN2022	• Appendix B closed to enrollment on 27FEB2022;<br>• Removed appendix for combination fluvoxamine maleate and fluticasone furoate arm;<br>• Added Appendix E – Fluvoxamine Maleate 100;<br>• Added neuropsychiatric events of special interest in Montelukast appendix;<br>• Updated the final visit from 90 to 120 days, effective only for arms

	Fluvoxamine Maleate 100 and Montelukast (footnotes added throughout for Day 120);<br>• Other administrative changes throughout.
Version 7.0, 08DEC2022	• Added C-SSRS collection to the Fluvoxamine Maleate 100 Appendix on Day 7 and Day 14 and included in the Schedule of Events (Section 8.1.2, 20.5, and **Table 1**);<br>• Changed the Final Visit to Day 180 for participants consented to protocol v7.0 (Section 3, 5.3.2, 8.1.3, 8.1.4, 9.1.2, 10.6.8, and **Table 1**);<br>• Added PASC Symptom Questionnaire (Section 8.1.4, 8.2, and **Table 1**);<br>• Added C-SSRS and PASC as abbreviations;<br>• Other administrative changes throughout.

15. References

1. *COVID-19 Map FAQ*. 2020 [cited 2020; Available from: https://coronavirus.jhu.edu/map-faq.html.
2. McIntosh, K. *Coronavirus disease 2019*. 2020 [cited 2020 March 24].
3. Beigel, J.H., et al., *Remdesivir for the Treatment of Covid-19 — Final Report.* New England Journal of Medicine, 2020. **383**(19): p. 1813-1826.
4. *Dexamethasone in Hospitalized Patients with Covid-19 — Preliminary Report.* New England Journal of Medicine, 2020.
5. Gordon, A.C., et al., *Interleukin-6 Receptor Antagonists in Critically Ill Patients with Covid-19 – Preliminary report.* medRxiv, 2021: p. 2021.01.07.21249390.
6. Baden, L.R., et al., *Efficacy and Safety of the mRNA-1273 SARS-CoV-2 Vaccine.* New England Journal of Medicine, 2020. **384**(5): p. 403-416.
7. Polack, F.P., et al., *Safety and Efficacy of the BNT162b2 mRNA Covid-19 Vaccine.* New England Journal of Medicine, 2020. **383**(27): p. 2603-2615.
8. Voysey, M., et al., *Safety and efficacy of the ChAdOx1 nCoV-19 vaccine (AZD1222) against SARS-CoV-2: an interim analysis of four randomised controlled trials in Brazil, South Africa, and the UK.* The Lancet, 2021. **397**(10269): p. 99-111.
9. Wang, Z., et al., *mRNA vaccine-elicited antibodies to SARS-CoV-2 and circulating variants.* bioRxiv, 2021: p. 2021.01.15.426911.
10. Grein, J., et al., *Compassionate Use of Remdesivir for Patients with Severe Covid-19.* New England Journal of Medicine, 2020. **382**(24): p. 2327-2336.
11. Beigel, J.H., et al., *Remdesivir for the Treatment of Covid-19 — Preliminary Report.* New England Journal of Medicine, 2020.
12. Wyatt, M.A., et al., *Implementation of the "Pregnancy Reasonably Excluded Guide" for Pregnancy Assessment: A Quality Initiative in Outpatient Gynecologic Surgery.* Obstetrics & Gynecology, 2018. **132**(5): p. 1222-1228.
13. Print, W.H.O.R.D.B. *Novel Coronavirus COVID-19 Therapeutic Trial Synopsis*. 2020.
14. Cabrera Martimbianco, A.L., et al., *Frequency, signs and symptoms, and criteria adopted for long COVID-19: A systematic review.* Int J Clin Pract, 2021. **75**(10): p. e14357.
15. Huang, C., et al., *6-month consequences of COVID-19 in patients discharged from hospital: a cohort study.* The Lancet, 2021. **397**(10270): p. 220-232.
16. Hirschtick, J.L., et al., *Population-Based Estimates of Post-acute Sequelae of Severe Acute Respiratory Syndrome Coronavirus 2 (SARS-CoV-2) Infection (PASC) Prevalence and Characteristics.* Clin Infect Dis, 2021. **73**(11): p. 2055-2064.
17. Bell, M.L., et al., *Post-acute sequelae of COVID-19 in a non-hospitalized cohort: Results from the Arizona CoVHORT.* PLOS ONE, 2021. **16**(8): p. e0254347.
18. Davis, H.E., et al., *Characterizing long COVID in an international cohort: 7 months of symptoms and their impact.* eClinicalMedicine, 2021. **38**.

19. Cella, D., et al., *The Patient-Reported Outcomes Measurement Information System (PROMIS): progress of an NIH Roadmap cooperative group during its first two years.* Med Care, 2007. **45**(5 Suppl 1): p. S3-S11.

20. Hays, R.D., et al., *PROMIS(®)-29 v2.0 profile physical and mental health summary scores.* Quality of life research : an international journal of quality of life aspects of treatment, care and rehabilitation, 2018. **27**(7): p. 1885-1891.

21. Whitehead, J., *Sample size calculations for ordered categorical data.* Stat Med, 1993. **12**(24): p. 2257-71.

22. Boussinesq, M., et al., *Clinical picture, epidemiology and outcome of Loa-associated serious adverse events related to mass ivermectin treatment of onchocerciasis in Cameroon.* Filaria journal, 2003. **2 Suppl 1**(Suppl 1): p. S4-S4.

23. Makenga Bof, J.C., et al., *Onchocerciasis control in the Democratic Republic of Congo (DRC): challenges in a post-war environment.* Trop Med Int Health, 2015. **20**(1): p. 48-62.

24. Chandler, R.E., *Serious Neurological Adverse Events after Ivermectin-Do They Occur beyond the Indication of Onchocerciasis?* Am J Trop Med Hyg, 2018. **98**(2): p. 382-388.

25. Barkwell, R. and S. Shields, *Deaths associated with ivermectin treatment of scabies.* Lancet, 1997. **349**(9059): p. 1144-5.

26. MERCK & CO., I. *TABLETS STROMECTOL (R) (IVERMECTIN) Product Label*. 2009 [cited 2021 February 12]; Available from: file:///U:/Personal/My%20Documents/Medical%20Writing/COVID/ACTIV-6/Study%20Drugs/Ivermectin.pdf.

27. Nicolas, P., et al., *Safety of oral ivermectin during pregnancy: a systematic review and meta-analysis.* The Lancet Global Health, 2020. **8**(1): p. e92-e100.

28. Pacqué, M., et al., *Pregnancy outcome after inadvertent ivermectin treatment during community-based distribution.* The Lancet, 1990. **336**(8729): p. 1486-1489.

29. Addiss, D.G., et al., *Randomised placebo-controlled comparison of ivermectin and albendazole alone and in combination for Wuchereria bancrofti microfilaraemia in Haitian children.* The Lancet, 1997. **350**(9076): p. 480-484.

30. Dreyer, G., et al., *Treatment of bancroftian filariasis in Recife, Brazil: a two-year comparative study of the efficacy of single treatments with ivermectin or diethylcarbamazine.* Trans R Soc Trop Med Hyg, 1995. **89**(1): p. 98-102.

31. Heukelbach, J., S. Franck, and H. Feldmeier, *Therapy of tungiasis: a double-blinded randomized controlled trial with oral ivermectin.* Mem Inst Oswaldo Cruz, 2004. **99**(8): p. 873-6.

32. Martin-Prevel, Y., et al., *Tolerance and efficacy of single high-dose ivermectin for the treatment of loiasis.* Am J Trop Med Hyg, 1993. **48**(2): p. 186-92.

33. Smit, M.R., et al., *Safety and mosquitocidal efficacy of high-dose ivermectin when co-administered with dihydroartemisinin-piperaquine in Kenyan adults with uncomplicated*

malaria (IVERMAL): a randomised, double-blind, placebo-controlled trial. Lancet Infect Dis, 2018. **18**(6): p. 615-626.

34. LLC, E.P., *Ivermectin Tablets USP, 7mg, 14mg Investigator's Brochure*. 2021.
35. Yang, S.N.Y., et al., *The broad spectrum antiviral ivermectin targets the host nuclear transport importin α/β1 heterodimer.* Antiviral Res, 2020. **177**: p. 104760.
36. Caly, L., et al., *The FDA-approved drug ivermectin inhibits the replication of SARS-CoV-2 in vitro.* Antiviral Research, 2020. **178**: p. 104787.
37. Lehrer, S. and P.H. Rheinstein, *Ivermectin Docks to the SARS-CoV-2 Spike Receptor-binding Domain Attached to ACE2.* In Vivo, 2020. **34**(5): p. 3023-3026.
38. Guzzo, C.A., et al., *Safety, tolerability, and pharmacokinetics of escalating high doses of ivermectin in healthy adult subjects.* Journal of clinical pharmacology, 2002. **42**(10): p. 1122-1133.
39. Chaccour, C., et al., *Ivermectin and COVID-19: Keeping Rigor in Times of Urgency.* Am J Trop Med Hyg, 2020. **102**(6): p. 1156-1157.
40. Arshad, U., et al., *Prioritization of Anti-SARS-Cov-2 Drug Repurposing Opportunities Based on Plasma and Target Site Concentrations Derived from their Established Human Pharmacokinetics.* Clin Pharmacol Ther, 2020. **108**(4): p. 775-790.
41. Bray, M., et al., *Ivermectin and COVID-19: A report in Antiviral Research, widespread interest, an FDA warning, two letters to the editor and the authors' responses.* Antiviral research, 2020. **178**: p. 104805-104805.
42. Zhang, X., et al., *Ivermectin inhibits LPS-induced production of inflammatory cytokines and improves LPS-induced survival in mice.* Inflamm Res, 2008. **57**(11): p. 524-9.
43. Ci, X., et al., *Avermectin exerts anti-inflammatory effect by downregulating the nuclear transcription factor kappa-B and mitogen-activated protein kinase activation pathway.* Fundam Clin Pharmacol, 2009. **23**(4): p. 449-55.
44. DiNicolantonio, J.J., J. Barroso-Arranda, and M. McCarty, *Ivermectin may be a clinically useful anti-inflammatory agent for late-stage COVID-19.* Open Heart, 2020. **7**(2): p. e001350.
45. Ahmed, S., et al., *A five-day course of ivermectin for the treatment of COVID-19 may reduce the duration of illness.* Int J Infect Dis, 2021. **103**: p. 214-216.
46. Chachar, A.Z.K., et al., *Effectiveness of Ivermectin in SARS-CoV-2/COVID-19 Patients.* International Journal of Sciences, 2021.
47. Abu Taiub Mohammed Mohiuddin, C., et al., *A Randomized Trial of Ivermectin-Doxycycline and Hydroxychloroquine-Azithromycin therapy on COVID19 patients.* Research Square, 2021.
48. Soto-Becerra, P., et al., *Real-World Effectiveness of Hydroxychloroquine, Azithromycin, and Ivermectin Among Hospitalized COVID-19 Patients: Results of a Target Trial Emulation Using Observational Data from a Nationwide Healthcare System in Peru.* SSRN Electronic Journal, 2020.

49. Hashim, H.A., et al., *Controlled randomized clinical trial on using Ivermectin with Doxycycline for treating COVID-19 patients in Baghdad, Iraq.* medRxiv, 2020: p. 2020.10.26.20219345.

50. Khan, M.S.I., et al., *Ivermectin Treatment May Improve the Prognosis of Patients With COVID-19.* Arch Bronconeumol, 2020. **56**(12): p. 828-830.

51. Morgenstern, J., et al., *The use of compassionate Ivermectin in the management of symptomatic outpatients and hospitalized patients with clinical diagnosis of COVID-19 at the Medical Center Bournigal and the Medical Center Punta Cana, Rescue Group, Dominican Republic, from may 1 to august 10, 2020.* medRxiv, 2020: p. 2020.10.29.20222505.

52. Krolewiecki, A., et al., *Antiviral Effect of High-Dose Ivermectin in Adults with COVID-19: A Pilot Randomised, Controlled, Open Label, Multicentre Trial*. 2020, SSRN.

53. Podder, C.S., et al., *Outcome of ivermectin treated mild to moderate COVID-19 cases: a single-centre, open-label, randomised controlled study.* IMC Journal of Medical Science, 2020. **14**(2).

54. Ravikirti, et al., *Ivermectin as a potential treatment for mild to moderate COVID-19 – A double blind randomized placebo-controlled trial.* medRxiv, 2021: p. 2021.01.05.21249310.

55. Morteza Shakhsi, N., et al., Research Square, 2021.

56. Espitia-Hernandez, G., et al., *Effects of Ivermectin-azithromycin-cholecalciferol combined therapy on COVID-19 infected patients: A proof of concept study.* Biomedical Research, 2020.

57. Babalola, O., et al., *Ivermectin shows clinical benefits in mild to moderate COVID19: A randomised controlled double blind dose response study in Lagos.* medRxiv, 2021: p. 2021.01.05.21249131.

58. NIH. *COVID-19 Treatment Guidelines Fluvoxamine*. 2021 [cited 2021 April 23]; Available from: https://www.covid19treatmentguidelines.nih.gov/immunomodulators/fluvoxamine/.

59. Apotex, *Higlights of Fluvoxamine Maleate Tablets Prescribing Information*.

60. Lenze, E.J., et al., *Fluvoxamine vs Placebo and Clinical Deterioration in Outpatients With Symptomatic COVID-19: A Randomized Clinical Trial.* JAMA, 2020. **324**(22): p. 2292-2300.

61. Rosen, D.A., et al., *Modulation of the sigma-1 receptor–IRE1 pathway is beneficial in preclinical models of inflammation and sepsis.* Science Translational Medicine, 2019. **11**(478): p. eaau5266.

62. Rafiee, L., V. Hajhashemi, and S.H. Javanmard, *Fluvoxamine inhibits some inflammatory genes expression in LPS/stimulated human endothelial cells, U937 macrophages, and carrageenan-induced paw edema in rat.* Iran J Basic Med Sci, 2016. **19**(9): p. 977-984.

63. Seftel, D. and D.R. Boulware, *Prospective Cohort of Fluvoxamine for Early Treatment of Coronavirus Disease 19.* Open Forum Infectious Diseases, 2021. **8**(2).

64. GlaxoSmithKline, *Highlights of Prescribing Information for Arnuity Ellipta*. 2018: Online.

65. Yamaya, M., et al., *Inhibitory effects of glycopyrronium, formoterol, and budesonide on coronavirus HCoV-229E replication and cytokine production by primary cultures of human nasal and tracheal epithelial cells.* Respir Investig, 2020. **58**(3): p. 155-168.

66. Peters, M.C., et al., *COVID-19-related Genes in Sputum Cells in Asthma. Relationship to Demographic Features and Corticosteroids.* Am J Respir Crit Care Med, 2020. **202**(1): p. 83-90.

67. Finney, L.J., et al., *Inhaled corticosteroids downregulate the SARS-CoV-2 receptor ACE2 in COPD through suppression of type I interferon.* J Allergy Clin Immunol, 2021. **147**(2): p. 510-519.e5.

68. Kern, C., et al., *Modeling of SARS-CoV-2 Treatment Effects for Informed Drug Repurposing.* Front Pharmacol, 2021. **12**: p. 625678.

69. Krolewiecki, A., et al., *Antiviral effect of high-dose ivermectin in adults with COVID-19: A proof-of-concept randomized trial.* EClinicalMedicine, 2021. **37**.

70. Navarro, M., et al., *Safety of high-dose ivermectin: a systematic review and meta-analysis.* J Antimicrob Chemother, 2020. **75**(4): p. 827-834.

71. Smit, M.R., et al., *Safety and mosquitocidal efficacy of high-dose ivermectin when co-administered with dihydroartemisinin-piperaquine in Kenyan adults with uncomplicated malaria (IVERMAL): a randomised, double-blind, placebo-controlled trial.* The Lancet Infectious Diseases, 2018. **18**(6): p. 615-626.

72. Reis, G., et al., *Effect of early treatment with fluvoxamine on risk of emergency care and hospitalisation among patients with COVID-19: the TOGETHER randomised, platform clinical trial.* The Lancet Global Health, 2022. **10**(1): p. e42-e51.

73. Barré, J., J.-M. Sabatier, and C. Annweiler, *Montelukast Drug May Improve COVID-19 Prognosis: A Review of Evidence.* Frontiers in Pharmacology, 2020. **11**(1344).

74. Copertino, D.C., et al., *Montelukast drug activity and potential against severe acute respiratory syndrome coronavirus 2 (SARS-CoV-2).* J Med Virol, 2021. **93**(1): p. 187-189.

75. Sanghai, N. and G.K. Tranmer, *Taming the cytokine storm: repurposing montelukast for the attenuation and prophylaxis of severe COVID-19 symptoms.* Drug Discov Today, 2020. **25**(12): p. 2076-2079.

76. Al-Kuraishy, H.M., et al., *Role of leukotriene pathway and montelukast in pulmonary and extrapulmonary manifestations of Covid-19: The enigmatic entity.* Eur J Pharmacol, 2021. **904**: p. 174196.

77. Mao, L., et al., *Neurologic Manifestations of Hospitalized Patients With Coronavirus Disease 2019 in Wuhan, China.* JAMA Neurology, 2020. **77**(6): p. 683-690.

78. Marschallinger, J., et al., *Structural and functional rejuvenation of the aged brain by an approved anti-asthmatic drug.* Nat Commun, 2015. **6**: p. 8466.

79. Khan, A.R., et al., *Montelukast in hospitalized patients diagnosed with COVID-19.* The Journal of asthma : official journal of the Association for the Care of Asthma, 2021: p. 1-7.

80. Kerget, B., et al., *Effect of montelukast therapy on clinical course, pulmonary function, and mortality in patients with COVID-19.* J Med Virol, 2022. **94**(5): p. 1950-1958.

16. Appendix A (Enrollment Closed 04FEB2022) – Ivermectin 400

16.1. Risk Assessment

The most commonly reported adverse events for ivermectin are pruritus (25.3%), headache (13.9%), and dizziness (7.5%) (ICH working group, 2003). Since the 1980s, the use of ivermectin in humans for the treatment of filariasis, especially onchocerciasis, has been associated with SAEs, including coma, seizure and death only when administered in regions where *onchocerciasis* and *loiasis* are co-endemic.[22]

The issue of SAEs due to ivermectin is a threat to adherence to mass drug administration and the control and elimination of disease in communities co-endemic with *O. volvulus* and *Loa*. Mass drug administration were suspended from 2004 to 2006 in some health zones in the Equateur, Bas Congo, and Oriental provinces in the Democratic Republic of the Congo because of ivermectin-associated SAEs in the Bas Congo and Oriental provinces in 2003.[23] It has been thought that co-infection with *L. loa* is a risk factor for the development of these reactions.[24] Additionally, a study investigating escalating high doses of ivermectin in healthy adults was performed to explore the safety of its use in the treatment of head lice. The authors documented no evidence of Central Nervous System (CNS) toxicity in doses up to 10 times the highest FDA-approved dose of 200 µg/kg.[25]

Table 3 shows safety events that occurred in greater than 5% of the clinical trial population who received ≥ 300 µg/kg of ivermectin.[26] For a detailed list of the adverse reactions that occurred during clinical trials with ivermectin, refer to the product label and the Investigator's Brochure.

Ivermectin is pregnancy category C and is not contraindicated per FDA. Analyses of outcomes of pregnancies in women inadvertently exposed to ivermectin through mass administration campaigns show no evidence of increased birth defects, neonatal deaths, maternal morbidity, preterm births, or low birthweight over baseline rates.[27, 28]

Table 3: Ivermectin Adverse Event Table for Doses ≥ 300 µg/kg

Indication (dose)	*Wuchererua bancrofti* (200-400 µg/kg) [29]	*Wuchererua bancrofti* (200 µg/kg, then 400 µg/kg on day 4) [30]	Stage II or III *tungiasis* lesions (300 µg/kg, days 1 & 2) [31]	*Loa loa* infection (300 or 400 µg/kg) [32]	Symptomatic *Plasmodium falciparum* malaria (600 µg/kg) [33]
Adverse Reactions					
Abdominal Pain			7%		
Cough	42%				
Diarrhea				6.5%	
Eye disorders					9%

Fever	69%	46%		8%	
Gastrointestinal		18%			
Headache	75%		11%	16%	
Local Inflammation		9%			
Local Pain		9%			
Lumber myalgia				6.5%	
Malaise		27%			
Myalgia	37%				
Neurological		15%			
Pneumonia					9%
Pruritus				60%	
Renal		33%			
Respiratory		33%			

16.2. Additional Appendix-Level Exclusion Criteria

1. End-stage renal disease on renal replacement therapy
2. Liver failure or decompensated cirrhosis
3. Current or planned use of the following drugs during the study, listed by drug class:
    a. Antiarrhythmic/antihypertensive drug class: quinidine, amiodarone, diltiazem, spironolactone, verapamil
    b. Antibiotic-macrolides drug class: clarithromycin, erythromycin
    c. Antifungal drug class: itraconazole, ketoconazole
    d. Immunosuppresant drug class: cyclosporine, tacrolimus
    e. Anti-HIV drug class: indinavir, ritonavir
4. Nursing mothers
5. Pregnancy*

**Participants must agree to use an effective method of contraception during study drug administration and for at least 3 days after their final dose of study drug. Effective methods include any of the following: abstinence, partner vasectomy, bilateral tubal ligation, intrauterine device, progestin implants, or barrier (condom, diaphragm, cervical cap) plus spermicide.*

16.2.1. Precautions

While rare, post-marketing reports indicate an increased International Normalized Ratio (INR) when ivermectin was co-administered with warfarin. With a 3-day dosing period this is unlikely

to be a significant issue, but INR monitoring can be recommended to the care provider if felt warranted by site investigator.

16.3. Ivermectin Information

Ivermectin is a semisynthetic oral agent used primarily as an anti-parasitic agent. It is derived from highly active, broad-spectrum, anti-parasitic agents isolated from *Streptomyces avermitilis* fermentation products. It binds selectively, and with high affinity, to glutamate-gated chloride ion channels, therefore increasing cell membrane permeability to chloride ions resulting in death of the parasite.[34] It is currently FDA-approved for the following indications: strongyloidiasis of the intestinal tract due to the nematode parasite *Strongyloides stercoralis* and onchocerciasis due to the nematode parasite *Onchocerca volvulus*.[26] Per the FDA-approved labelling of ivermectin, the recommended dose is 200 µg/kg/day to treat strongyloidiases and 150 µg/kg/day to treat onchocerciasis in the form of a 3 mg tablet.

16.3.1. Formulation, Appearance, Packaging, and Labeling

Ivermectin is a white to yellowish-white, nonhygroscopic, crystalline powder. For this study, ivermectin will be supplied as fifteen 7-mg tablets in a bottle with a single panel label. The tablets are white, round, biconvex tablets with "123" over the scoring on one side. All packaging will be labeled to indicate that the product is for investigational use.

16.3.2. Drug Dispensing, Storage, and Stability

Ivermectin will be supplied as 7-mg tablets and must be stored at temperatures below 30°C.

16.3.3. Dosing and Administration

Ivermectin should be taken on an empty stomach with water (30 minutes before a meal or 2 hours after a meal). Each participant will receive a bottle of fifteen 7-mg tablets and will be instructed to take a pre-specified number of tablets for 3 consecutive days based on their weight (see **Table 4**) for a daily dose of approximately 300-400 µg/kg.

Table 4: Ivermectin 400 Dosing Schedule

Weight (kg)	Day 1 (# of 7-mg tablets)	Day 2 (# of 7-mg tablets)	Day 3 (# of 7-mg tablets)	Daily Dose (µg/kg)
35-52	2	2	2	269-400
53-69	3	3	3	304-396
70-89	4	4	4	315-400
> 90	5	5	5	≤ 389

16.3.4. Rationale for Selection of Dose

Pre-clinical studies:

Reports from *in vitro* studies suggest that ivermectin acts against ribonucleic acid (RNA) viruses such as SARS-CoV-2 by inhibiting the host importin α/β-mediated nuclear transport that prevent

viral proteins from entering the nucleus to alter host cell function.[35] A single dose addition of ivermectin to Vero-hSLAM cells 2 hours post infection with SARS-CoV-2 was able to effect a 5000-fold reduction in viral RNA at 48 hours and may interfere with the attachment of SARS-CoV-2 spike protein to the human cell membrane.[36, 37]

Ivermectin has been shown to inhibit the replication of SARS-CoV-2 in cell culture. However, pharmacokinetic and pharmacodynamic studies suggest that ivermectin doses up to 100-fold higher than those approved for use in humans would be required to achieve the plasma concentrations necessary to duplicate the drug's antiviral efficacy *in vitro*.[38, 39] Even though ivermectin appears to accumulate in lung tissue, with the doses used in most clinical trials, predicted systemic plasma and lung tissue concentrations are much lower than 2 μM, the half-maximal inhibitory concentration (IC_{50}) against SARS-CoV-2 *in vitro*.[40, 41]

Ivermectin demonstrates potential anti-inflammatory properties in some *in vitro* studies, properties which have been postulated to be beneficial in the treatment of COVID-19.[42-44] The dose range for an anti-inflammatory effect may be lower than for the anti-viral effects.[44]

Clinical studies:

A number of retrospective cohort studies and the results of several randomized trials of ivermectin use in patients with COVID-19 have been published in peer-reviewed journals or made available as preliminary, non-peer-reviewed reports. Some clinical studies showed no benefits after ivermectin use, whereas others reported shorter time to resolution of disease manifestations attributed to COVID-19, greater reduction in inflammatory markers, shorter time to viral clearance, or lower mortality rates in patients who received ivermectin than in patients who received comparator drugs or placebo.[45-50]

Most of the studies reported to date are limited by their small sample size, varying dosing schedules and the adjunctive use of various concomitant medications (e.g., doxycycline, hydroxychloroquine, azithromycin, zinc, corticosteroids), confounding assessment of the true efficacy or safety of ivermectin. Clinical studies in outpatients with COVID-19 did not always describe the severity of COVID-19 and the study outcome measures were not always defined. Nonetheless, while some have shown no difference, most clinical trials of outpatients with mild/moderate COVID-19 have shown clinical improvement with even a single dose or a short two to five-day course of ivermectin given orally shortly after symptom onset.[39, 49, 51, 52]

There is a dose-dependent relationship between ivermectin dose and clinical efficacy. Trials using lower doses of ivermectin tend to show no or minimal clinical benefit in COVID-19 patients treated in the outpatient or hospital setting.[53, 54] Higher doses, at least 0.4 mg/kg, particularly when administered in multiple doses, have been shown to significantly reduce time to recovery and mortality as compared with lower doses or placebo/standard care.[51, 55-57] There is a concentration-dependent virologic response seen using higher-than-usual doses of ivermectin (600 μg/kg vs 200 μg/kg once daily for 5 days) that have been shown to significantly reduce the time to PCR viral positivity over standard doses with minimal associated toxicities.[52]

The safety, tolerability, and pharmacokinetics of escalating high doses of ivermectin was tested in healthy adult subjects.[38] Doses from 30 mg/day **(347-571 μg/kg)** to 120 mg/day **(1404-2000**

µg/kg) given for 3 days were all well tolerated.[38] These data coupled with evidence of superior clinical efficacy of doses in the lower of this range provide the rationale for the 300-400 µg/kg dose given for 3 days in the proposed trial.

16.4. Placebo Information

16.4.1. Formulation, Appearance, Packaging, and Labeling

Placebo will match the appearance of the 7-mg ivermectin tablets: white, round, biconvex tablets with "123" over the scoring on one side. A total of fifteen tablets will be provided in a bottle with a single panel label. The placebo formulation includes the following ingredients in a 210 mg tablet: microcrystalline cellulose, NF (MC-102; pregelatinized starch, NF (Starch 1500); croscarmellose sodium, NF (Vivasol, GF Grade); colloidal silicon dioxide, NF (Aerosil 200) and magnesium stearate, NF (2257). All packaging will be labeled to indicate that the product is for investigational use.

16.4.2. Drug Dispensing, Storage, and Stability

Placebo must be stored at temperatures below 30°C.

16.4.3. Dosing and Administration

Dosing and administration will occur according to Section 16.3.3 in order to maintain blinding.

16.5. Events of Special Interest

None

17. Appendix B (Enrollment Closed 27May2022) – Fluvoxamine Maleate

17.1. Risk Assessment

The most common adverse effects of fluvoxamine described in the setting of treatment of psychiatric conditions include gastrointestinal effects, neurological effects, dermatological reactions, and in rare cases suicidal ideation.[58] In two 10-week controlled trials in Obsessive Compulsive Disorder (OCD) and depression at doses ranging from 100-300 mg/day, the most commonly observed adverse reactions associated with the use of fluvoxamine maleate tablets (incidence of 5% or greater and at least twice that for placebo) were nausea, somnolence, insomnia, asthenia, nervousness, dyspepsia, abnormal ejaculation, sweating, anorexia, tremor, and vomiting (see **Table 5**).[59]

Table 6 lists the complete set of adverse events identified in a randomized clinical trial of fluvoxamine (300 mg/day for 15 days) versus placebo in 152 COVID-19 participants.[60] Adverse events and serious adverse events were more common in the placebo arm and the single SAE in the fluvoxamine arm was dehydration and the study medication was not interrupted.[60]

Table 5. Fluvoxamine Adverse events occurring in 10-week studies of adult OCD or depression

*Table shows events that occurred in ≥ 5% of adult OCD and depression study participants receiving 100-300 mg/day for 10 weeks of fluvoxamine maleate versus placebo.***[59]**

Adverse Reaction	Fluvoxamine, n=892 (%)	Placebo, n=778 (%)
Headache	22	20
Asthenia	14	6
Nausea	40	14
Diarrhea	11	7
Constipation	10	8
Dyspepsia	10	5
Anorexia	6	2
Vomiting	5	2
Somnolence	22	8
Insomnia	21	10
Dry Mouth	14	10
Nervousness	12	5
Dizziness	11	6
Tremor	5	1

Anxiety	5	3

Table 6. Fluvoxamine Adverse events that occurred in COVID-19 study participants receiving 300 mg/day for 15 days.

Adverse Reaction **[60]**	Fluvoxamine, n=80 (%)	Placebo, n=72 (%)
Pneumonia	3.8	8.3
Shortness of breath	2.5	5.6
Headache or head pain	2.5	1.4
Gastroenteritis, nausea, or vomiting	1.3	6.9
Muscle aches	1.3	0
Bacterial infection	1.3	0
Vasovagal syncope	1.3	0
Teeth chattering	1.3	0
Dehydration	1.3	0
Low oxygen saturation or hypoxia	0	8.3
Chest pain or tightness	0	2.8
Fever	0	2.8
Acute respiratory failure	0	1.4
Serious adverse events	1.3	6.9
Other adverse events	13.8	8.3

17.2. Additional Appendix-Level Exclusion Criteria

1. Use of selective serotonin (or norepinephrine) reuptake inhibitors (SSRIs/SNRIs), including fluvoxamine, or monoamine oxidase inhibitors (MAOIs) within 2 weeks of consent including triptans and tryptophan. Use of fluoxetine within 45 days of consent.
2. Co-administration of tizanidine, thioridazine, alosetron, pimozide, diazepam, ramelteon, linezolid
3. Bipolar Disorder
4. Nursing mothers
5. Pregnancy*

**Participants must agree to use an effective method of contraception during study drug administration and for at least 3 days after their final dose of study drug. Effective methods include any of the following: abstinence, partner vasectomy, bilateral tubal ligation, intrauterine device, progestin implants, or barrier (condom, diaphragm, cervical cap) plus spermicide.*

17.2.1. Precautions

Fluvoxamine is a potent inhibitor of CYP1A2 and 2C19 and a moderate inhibitor of CYP2C9, 2D6, and 3A4, as such, it may enhance anticoagulant effects of antiplatelets and anticoagulants as well as other medications. It is recommended that concomitant medications listed below be discussed with participants and potential effects of increased drug exposure reviewed and monitored by the participant and/or their prescribing clinician.

- Tricyclic antidepressants: monitor for side effects with amitriptyline, clomipramine, imipramine
- Antipsychotic drugs: neuroleptic malignant syndrome or similar; particularly clozapine (hypotension, seizure)
- Benzodiazepines: particularly alprazolam; recommend dose reduction
- Tramadol, buspirone, fentanyl, lithium, amphetamines, St. John's Wart, carbamazepine, quinidine, and tacrine
- Methadone: opioid intoxication
- Mexiletine: monitor for side effects
- Theophylline: recommend dose reduction and monitor for side effects
- Warfarin: monitor INR
- NSAIDS or aspirin: monitor for signs of bleeding
- Diltiazem, propranolol, metoprolol: monitor for bradycardia

Participants should be warned about fluvoxamine inhibition of caffeine metabolism. A description is 1 cup of coffee has the effects of 4 cups while taking fluvoxamine.

Caution should be used in participants with hepatic dysfunction due to approximately 30% increase in exposures.

17.3. Fluvoxamine Information

Fluvoxamine is an FDA-approved SSRI for the treatment of OCD. Clinically it is also used for other conditions such as depression.[58] The active ingredient in fluvoxamine is fluvoxamine maleate.

17.3.1. Formulation, Appearance, Packaging, and Labeling

Fluvoxamine is a round golden 50 mg tablet that is scored on both sides - one side has "APO" and the other side has "F50" with a partial bisect. All packaging will be labeled to indicate that the product is for investigational use.

17.3.2. Drug Dispensing, Storage, and Stability

Drug will be supplied by Apotex and distributed by Belmar Pharmacy. Study drug should be stored in controlled room temperature (20°C to 25°C); excursions are permitted to 15°C to 30°C.

17.3.3. Dosing and Administration

Fluvoxamine will be self-administered orally by each participant at a dose of 50 mg BID for 10 days.

17.3.4. Rationale for Selection of Dose

The recommended starting dose of fluvoxamine for OCD in adults is 50 mg daily dose to be titrated up to a maximum of 300 mg/day divided into BID doses. Clinical data from the placebo-controlled, randomized trial in nonhospitalized adults with mild COVID-19 demonstrated that a 50 mg BID fluvoxamine dose was well-tolerated and effective (see **Figure 5**).[60] In the same study, doses of 100 mg BID in COVID-19 participants resulted in additional side effects over a 14-day period. The 300 mg daily dose is for serotonin receptor activity, whereas the postulated dose for sigma-1 receptor activity as an anti-inflammatory is lower. Therefore, the proposed dosing regimen of 50 mg BID for 10 days will use the anticipated minimal effective dose to maximize efficacy and minimize toxicity.

Pre-Clinical Studies:

Pre-clinical studies have indicated that the anti-inflammatory effects of fluvoxamine may support its use for treating COVID-19. Systemic inflammation as a result of infection can damage vasculature which may lead to tissue hypoperfusion and multiple organ failure.[61] Reducing systemic inflammation thereby may avoid or mitigate the aforementioned serious clinical outcomes. In murine studies, administration of fluvoxamine significantly increased survival of S1R wildtype (WT) mice and S1R knockout (KO) mice challenged with ligand lipopolysaccharide (LPS) as compared to mice that received saline (**Figure 4**).[61] Fluvoxamine also reduced production of inflammatory cytokines in *ex vivo* and *in vitro* murine and human cells (HEK293mTLR4/MD2/CD14, primary lung fibroblasts, and mouse bone marrow-derived macrophages) and inflammatory genes in human endothelial cells.[61, 62]

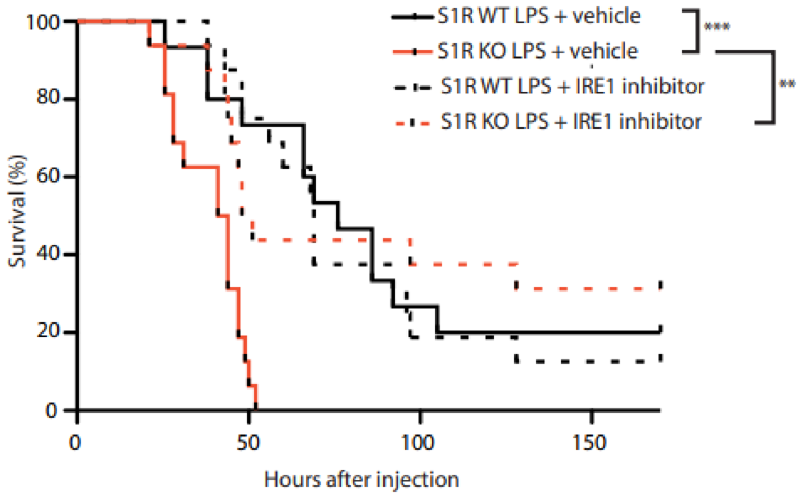

Figure 4: Survival curve of WT and S1R KO mice
Figure note: Mice were treated with vehicle (33% Kolliphor in saline) or STF (30 mg/kg) after administration of LPS (2 mg/kg) as indicated in (B) (n = 15 to 16 mice per group; **P < 0.01, ***P < 0.001, log-rank test).***[61]***

Clinical Studies:

A placebo-controlled, randomized trial in nonhospitalized adults with mild COVID-19 tested the efficacy and safety of fluvoxamine (50 mg oral one-time dose, followed by 100 mg orally twice daily for 2 days, followed by 100 mg orally three times daily through day 15) versus placebo ***[60]***. Results of the study reported that 8.3% (6/72) of participants who received placebo experienced clinical deterioration within 15 days of randomization, as opposed to 0% (0/80) in the fluvoxamine arm (absolute difference 8.7%; 95% CI, 1.8% to 16.5%; *P* = 0.009).[60] Clinical deterioration was defined as shortness of breath/pneumonia with hypoxia (**Figure 5**). Concomitant SSRI/SNRI dosing was not allowed per the trial protocol thus the participant's SSRI/SNRI was held for the study dosing period if reported at baseline.

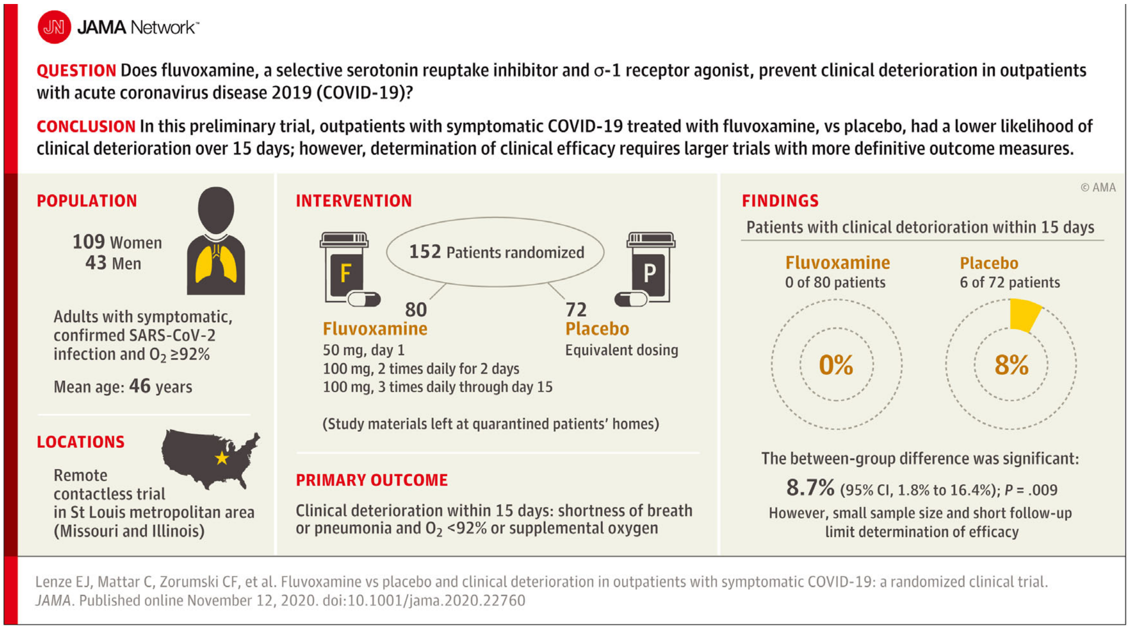

Figure 5: Summary of JAMA Randomization Clinical Trial of Fluvoxamine for Early COVID-19.

A prospective, nonrandomized observational cohort study that evaluated fluvoxamine showed that on Day 14, 0/65 participants had persistent symptoms as opposed to 19/48 participants who did not receive fluvoxamine (P < 0.001) ***[63]***. Additionally, by Day 14, none of the participants who received fluvoxamine were hospitalized as compared to 6 participants who were hospitalized who did not receive the drug (see **Figure 6**). In this study, all participants were offered fluvoxamine and decided whether or not to take the study drug. Participants who chose to receive the study drug received 50 mg of fluvoxamine two times a day following an upfront 100 mg loading dose.[63]

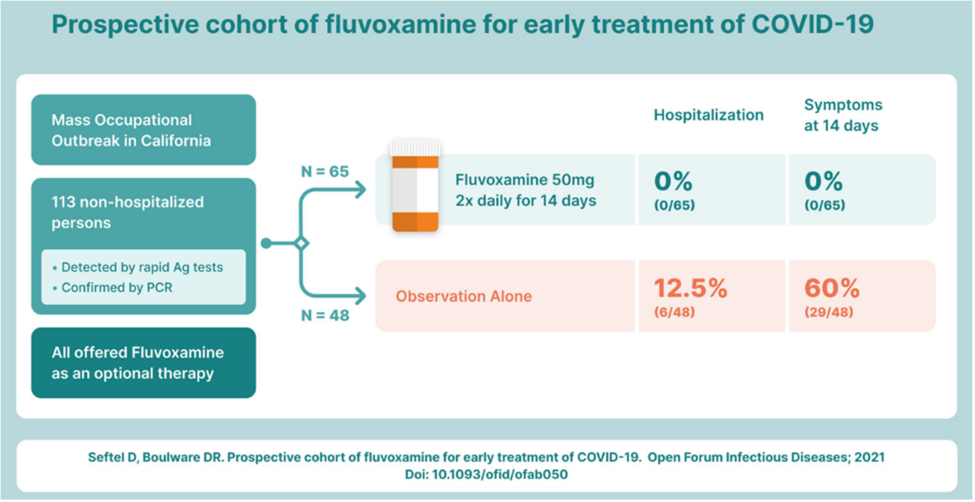

Figure 6: Summary of study results for the prospective, nonrandomized observational cohort study with fluvoxamine in participants diagnosed with COVID-19.

17.4. Placebo Information

The placebo will be a fluvoxamine-matched placebo containing mannitol, magnesium stearate, hydroxyethyl cellulose, polyethylene glycol, titanium dioxide, yellow ferric oxide, and purified water. The appearance and packaging will match that of the study drug (as described in Section 17.3.1). All packaging will be labeled to indicate that the product is for investigational use.

17.4.1. Formulation, Appearance, Packaging, and Labeling

Placebo will match the appearance of the fluvoxamine tablets: round golden 50 mg tablet that is scored on both sides - one side has “APO” and the other side has “F50” with a partial bisect. All packaging will be labeled to indicate that the product is for investigational use.

17.4.2. Drug Dispensing, Storage, and Stability

Placebo will be supplied by Apotex and distributed by Belmar Pharmacy. Placebo should be stored in controlled room temperature (20°C to 25°C).

17.4.3. Dosing and Administration

Participants will self-administer one placebo tablet orally, twice daily for 10 days.

17.5. Events of Special Interest

None

18. Appendix C (Enrollment Closed 08FEB2022) – Fluticasone Furoate

18.1. Risk Assessment

The most common adverse reactions reported in ≥ 5% of adults and adolescents with lung disease include nasopharyngitis, bronchitis, upper respiratory tract infection, and headache. Long-term use of systemic and local corticosteroids may also result in the following side effects: *Candida albicans* infection, immunosuppression, hypercorticism and adrenal suppression, reduction in bone mineral density (BMD), or glaucoma and cataracts.[64]

There are insufficient data on the use of fluticasone furoate in pregnant women. There are clinical considerations with use of fluticasone furoate in pregnant women to inform drug-associated risk and benefit. In animal reproduction studies, fluticasone furoate administered by inhalation to rats and rabbits during the period of organogenesis produced no fetal structural abnormalities. The highest fluticasone furoate doses in the rat and rabbit studies were 4 times and 1 times the maximum recommended human daily inhalation dose, respectively. The estimated risk of major birth defects and miscarriage for the indicated populations is unknown. In the US general population, the estimated risk of major birth defects and miscarriage in clinically recognized pregnancies is 2% to 4% and 15% to 20%, respectively.[64]

Disease-Associated Maternal and/or Embryofetal Risk: In women with poorly or moderately controlled asthma, there is an increased risk of several perinatal outcomes such as pre-eclampsia in the mother and prematurity, low birth weight, and small for gestational age in the neonate.[64]

Table 7. Fluticasone Adverse reactions that occurred in ≥3% of adults and adolescents with asthma in a 24-week trial.

Adverse Reaction ***[64]***	Fluticasone furoate, fluticasone 200 µg, n=119 (%)	Fluticasone furoate, fluticasone 100 µg, n=119 (%)
Nasopharyngitis	13	12
Headache	13	10
Bronchitis	7	12
Influenza	7	4
Upper Respiratory tract infection	6	2
Sinusitis	4	7
Oropharyngeal pain	4	3
Pharyngitis	3	6
Back pain	3	3

Dysphonia	3	2
Oral candidiasis	3	<1
Procedural pain	3	<1
Rhinitis	3	<1
Throat irritation	3	<1
Abdominal pain	3	0
Cough	3	0

18.2. Additional Appendix-Level Exclusion Criteria

1. Severe hypersensitivity to milk proteins
2. Currently prescribed or use within 30 days of inhaled or systemic steroids
3. Moderate to severe hepatic impairment, defined as Child-Pugh B or C
4. Nursing mothers
5. Pregnancy*

**Participants must agree to use an effective method of contraception during study drug administration and for at least 3 days after their final dose of study drug. Effective methods include any of the following: abstinence, partner vasectomy, bilateral tubal ligation, intrauterine device, progestin implants, or barrier (condom, diaphragm, cervical cap) plus spermicide.*

18.2.1. Precautions

While not contraindicated, the following should be considered while taking fluticasone furoate:

- Strong cytochrome P450 3A4 inhibitors (e.g., ketoconazole, ritonavir, clarithromycin, conivaptan, indinavir, itraconazole, lopinavir, nefazodone, nelfinavir, saquinavir, telithromycin, troleandomycin, voriconazole) should be used with caution as increased systemic corticosteroid adverse effects may occur in combination with fluticasone furoate. Generally, due to the limited study drug administration length, clinically significant interactions are unlikely.
- Paradoxical Bronchospasm may occur with an immediate increase in wheezing after dosing. Fluticasone furoate should be discontinued immediately if this occurs.

18.3. Fluticasone Furoate Information

Inhaled fluticasone furoate will be provided by GlaxoSmithKline. It is a synthetic trifluorinated inhaled corticosteroid (ICS) with anti-inflammatory activity. *In vitro* and *in vivo* models have shown that fluticasone furoate demonstrates anti-inflammatory actions by activating the

glucocorticoid response element, inhibiting pre-inflammatory transcription factors such as NFkB, and inhibiting antigen-induced lung eosinophilia, which may contribute to its efficacy in the approved indications. It is FDA-approved for once-daily maintenance treatment of asthma as prophylactic therapy in patients 5 years and older.[64]

18.3.1. Formulation, Appearance, Packaging, and Labeling

Fluticasone furoate is an inhaled powder drug product. It is a synthetic trifluorinated corticosteroid that is insoluble in water. Fluticasone furoate is a white powder. Fluticasone furoate will be provided in a two tone grey inhaler with a mouthpiece cover and separate foil blister strips. The blister strips used for this study will contain a white powder mix with a mixture of 200 µg micronized fluticasone furoate and lactose monohydrate. The inhaler will be packaged in a moisture-protective foil tray with a desiccant and a peelable lid.[64] All packaging will be labeled to indicate that the product is for investigational use.

18.3.2. Drug Dispensing, Storage, and Stability

The study drug should be stored at room temperature (20°C to 25°C) with excursions permitted from 15°C to 30°C. It should be stored in a dry environment away from direct heat or sunlight. Unopened fluticasone furoate should be stored inside the unopened moisture-protective foil tray and only removed immediately before use. Any unused study product should be discarded 6 weeks after opening the foil tray or when the counter reads "0" (after all blisters have been used), whichever comes first. The inhaler is not reusable.[64] The study product has a shelf life of 24 months when stored at ≤ 25°C. After removal of the secondary package and the desiccant packet from the inhaler, the product may be stored up to 6 weeks at ≤ 25°C.

18.3.3. Dosing and Administration

Fluticasone furoate is a self-administered inhaled drug. Participants will self-administer 200 µg (1 blister) of fluticasone furoate once daily for 14 days. After inhaler activation, the powder within the blister is exposed and the participant inhales the study drug through the mouthpiece.

18.3.4. Rationale for Selection of Dose

Inhaled corticosteroids (ICS) have been shown to be effective in improving asthma and as a combination therapy in chronic obstructive pulmonary disease (COPD) and thus are commonly prescribed medications world-wide. Corticosteroids have been shown to have a broad range of pharmacologic activity on multiple cell types including mast cells, eosinophils, neutrophils, macrophages and lymphocytes, as well as other mediators of inflammation such as histamine, eicosanoids, and leukotrienes. In addition to the anti-inflammatory role of ICS, there is also potential for regulation of gene transcription in epithelial cells.[65-67] This regulation of gene transcription may result in inhibition of SARS-CoV-2 replication. Furthermore, in asthmatic patients, ICS may lower gene expression of ACE2 and TMPRSS2. The ACE2 receptor is expressed on epithelial cells and binds SARS-CoV-2 for entry and the serine protease TMPRSS2 primes the SARS-CoV-2 spike protein for binding. Lowering gene expression of ACE2 and TMPRSS2 may reduce binding and entry into cells therefore reducing or preventing infection.

Clinical Studies:

The PRINCIPLE (Platform Randomized trial of Interventions against COVID-19 in Older People) trial is a multicenter, open-label, multi-arm, adaptive randomized, platform trial. The trial is ongoing and released an interim analysis after the Trial Steering Committee advised the Trial Management Group that the pre-specified superiority criterion was met on the time to recovery in the overall study population and the subgroup of participants with confirmed positive SARS-CoV-2 testing. Participants were eligible if aged ≥65 years, or ≥50 years with comorbidities and had ongoing symptoms from PCR confirmed or suspected COVID-19 with symptoms starting within the past 14 days. Participants were randomized to any open active intervention arm including inhaled budesonide and usual care. Participants were followed through an online, daily symptom diary for 28 days. Participants received usual care plus inhaled budesonide 800 µg daily for 14 days or usual care alone. The primary outcome of the trial at the start was hospitalization or death within 28 days; however, the rate of hospitalization was lower than initially expected thus the Trial Management Group and the Trial Steering Committee recommended amending the primary outcome to include a measure of illness duration. The trial was completed with two co-primary endpoints measured within 28 days of randomization: 1) time to first reported recovery defined as the first day that a participant reported feeling recovered; and 2) hospitalization or death related to COVID-19.

The interim analysis included eligible SARS-CoV-2 positive participants who were randomized to inhaled budesonide (N=751) or usual care alone (N=1028). The mean age was 62.8 years and 83% of participants had co-morbidities. The median days from symptom onset to enrollment was 6 days. There was evidence of a benefit in time-to-first-recovery in the budesonide arm with an estimated median benefit of 3 days. The point estimate of the proportion of COVID-19 related hospitalizations or deaths was lower in the budesonide group (8.5%) versus usual care (10.3%), but this did not meet statistical significance (95% BCI -0.7 – 4.8%). There were two SAEs for hospitalization unrelated to COVID-19, both in the budesonide group.

A smaller trial of 146 nonhospitalized adults with mild COVID-19 reported that inhaled budesonide at the same dose reduced COVID-19 related emergency assessments and hospitalization.

The dose of ICS studied is PRINCIPLE is consistent with a high dose of inhaled steroid. The equivalent high dose of fluticasone furoate is 200 µg/day. Inhaled fluticasone furoate has a greater anti-inflammatory potency per microgram than budesonide, thus fluticasone furoate is administered at a lower daily dose and used only once daily to achieve a similar high dose.

18.4. Placebo Information

18.4.1. Formulation, Appearance, Packaging, and Labeling

The placebo will be a fluticasone furoate-matched placebo containing lactose for inhalation in the same two tone grey inhaler that is used for the study drug. The appearance and packaging will match that of the study drug (as described in Section 18.3.1). All packaging will be labeled to indicate that the product is for investigational use.

18.4.2. Drug Dispensing, Storage, and Stability

Placebo will be stored in the same conditions as study drug, room temperature (20°C to 25°C) with excursions permitted from 15°C to 30°C in a dry environment away from direct heat or sunlight. The placebo has a shelf life of up to 36 months when stored at ≤ 30°C.

18.4.3. Dosing and Administration

Participants will self-administer one blister of placebo via inhalation from the inhaler once daily for 14 days.

18.5. Events of Special Interest

None

18.6. Safety Reporting for Fluticasone Furoate

Sponsor will promptly notify GlaxoSmithKline of all SAEs, UADEs, and pregnancies (if applicable), and medical device deficiencies that have occurred for participants enrolled in this Study Drug Appendix, in accordance with the timelines and procedures specified in the Protocol/appendix. In addition, the sponsor will reasonably obtain and provide follow-up information as available, to GSK upon request.

19. Appendix D – Ivermectin 600

19.1. Risk Assessment

Refer to Section 16.1.

19.2. Additional Appendix-Level Exclusion Criteria

Refer to Section 16.2.

19.2.1. Precautions

Refer to Section 16.2.1.

19.3. Ivermectin Information

Refer to Section 16.3.

19.3.1. Formulation, Appearance, Packaging, and Labeling

Refer to Section 16.3.1.

19.3.2. Drug Dispensing, Storage, and Stability

Refer to Section 16.3.2.

19.3.3. Dosing and Administration

Ivermectin should be taken on an empty stomach with water (30 minutes before a meal or 2 hours after a meal). Each participant will receive a bottle containing the number of 7-mg tablets they require based on their weight and will be instructed to take a pre-specified number of tablets for 6 consecutive days based on their weight (see **Table 8**) for a daily dose of approximately 400-600 µg/kg.

Table 8. Ivermectin 600 Dosing Schedule

Weight (kg)	Day 1 (# of 7-mg tablets)	Day 2 (# of 7-mg tablets)	Day 3 (# of 7-mg tablets)	Day 4 (# of 7-mg tablets)	Day 5 (# of 7-mg tablets)	Day 6 (# of 7-mg tablets)	Daily Dose (µg/kg)
35-52	3	3	3	3	3	3	403-600
53-69	4	4	4	4	4	4	406-528
70-89	6	6	6	6	6	6	470-600
90-109	7	7	7	7	7	7	450-540
110-129	8	8	8	8	8	8	434-509
> 129	10	10	10	10	10	10	< 540

19.3.4. Rationale for Selection of Dose

Refer to Section 16.3.4.

Additional relevant pre-clinical studies:

Modeling studies have supported high dose and longer duration of ivermectin. One modeling study reported 600 µg/kg daily for 3 days may have clinical effect while 300 µg/kg for 3 days was unlikely to have efficacy.[68] Following this modeling study, a recently published proof-of-concept randomized controlled clinical trial in hospitalized patients showed that 600 µg/kg ivermectin for 5 days, a dose still well within the range for safety, was associated with IC_{50} for anti-viral activity *in vitro* while lower doses (300 µg/kg) were not.[69] The anti-viral activity was identified in the subgroup of patients on ivermectin with higher mean plasma concentrations. When the ivermectin group was further divided into subgroups with 160 ng/mL as the plasma concentration cutoff, the median Cmax achieved was 202 ng/mL in those with plasma concentration >160 ng/mL and this group had greater reduction in viral load at day 5. These data argue for the use of higher dosing regimens in clinical trials.

The safety of higher doses of ivermectin is well understood. At least six published trials have administered ivermectin at doses above 400 µg/kg.[70] These include over 2,500 independent administrations of cumulative 800 µg/kg within one week and over 2,000 single dose administrations of 800 µg/kg. The primary side effect of higher doses, usually due to peak concentrations, is transient and mild visual disturbances not found to be structural and found to self-resolve. One such study randomized 47 patients with malaria to receive ivermectin 600 µg/kg daily for 3 days versus 300 µg/kg daily for 3 days versus placebo.[71] The 600 µg/kg group had more adverse events than the other groups. Treatment-related adverse events exhibited a dose-response relationship, with predominantly transient minor visual disturbances that were deemed non-severe.

19.4. Placebo Information

Refer to Section 16.4.

19.4.1. Formulation, Appearance, Packaging, and Labeling

Refer to Section 16.4.1.

19.4.2. Drug Dispensing, Storage, and Stability

Refer to Section 16.4.2.

19.4.3. Dosing and Administration

Dosing and administration will occur according to Section 19.3.3 in order to maintain blinding.

19.5. Events of Special Interest

- Photophobia
- Blurred vision
- Visual impairment

20. Appendix E – Fluvoxamine Maleate 100

20.1. Risk Assessment

Refer to Section 17.1.

20.2. Additional Appendix-Level Exclusion Criteria

Refer to Section 17.2.

20.2.1. Precautions

Refer to Section 17.2.1.

20.3. Fluvoxamine Information

Refer to Section 17.3.

20.3.1. Formulation, Appearance, Packaging, and Labeling

Refer to Section 17.3.1. Fluvoxamine tablets remain 50 mg.

20.3.2. Drug Dispensing, Storage, and Stability

Refer to Section 17.3.2.

20.3.3. Dosing and Administration

Fluvoxamine will be self-administered orally by each participant at a dose of 50 mg BID for one day (first two doses at 50 mg), followed by 100 mg (two 50 mg tablets) BID for 12 days (remaining 24 doses at 100 mg), for a total of 13 days (26 doses, 50 tablets) of fluvoxamine.

20.3.4. Rationale for Selection of Dose

Refer to Section 17.3.4.

Additional relevant rationale:

The recommended starting dose of fluvoxamine for OCD in adults is 50 mg daily to be titrated up to a maximum of 300 mg/day divided into BID doses. The 300 mg daily dose is for serotonin receptor activity, whereas the postulated dose for sigma-1 receptor activity as an anti-inflammatory is lower. The TOGETHER trial is a randomized, placebo-controlled, adaptive platform trial that demonstrated a 100 mg BID fluvoxamine dose for 10 days was well-tolerated and effective (see **Figure 7**) in nonhospitalized adults with mild COVID-19.[72] This is a multicenter study conducted in Brazil. Participants were > 18 years old and with symptomatic confirmed COVID-19 within 7 days of the screening date or a positive rapid antigen test done at the time of screening. Participants were required to have at least one risk factor for severe disease. Participants were randomized to active drug versus placebo, and due to the concurrent enrollment into multiple active arms, pooled placebo was used for each active arm. The primary outcome was a composite endpoint of medical admission to the hospital due to COVID-19, which was defined as more than 6 hours in an emergency care setting (acute care areas set up to respond to capacity) or referral for hospitalization, within 28 days of randomization. Key

secondary outcomes included viral clearance, time to clinical improvement, and all-cause mortality. The planned accrual, based on an assumption of 15% event rate in the placebo group, was 681 participants per arm.

The study randomized 741 participants to fluvoxamine and 756 to placebo. In the fluvoxamine group 79 (11%) participants had a primary outcome event versus 119 (16%) in the pooled placebo arm (relative risk 0.68 [95% Bayesian credible interval, 0.52-.88]). Secondary outcomes including viral clearance at day 7, hospitalization, and mortality were not different between arms. There was no significant difference in the number of treatment-emergent adverse events in the two arms. Thus, this large randomized, double-blind, controlled trial is suggestive of safety and efficacy of fluvoxamine dosed 100 mg twice daily in people with mild-to-moderate COVID-19.

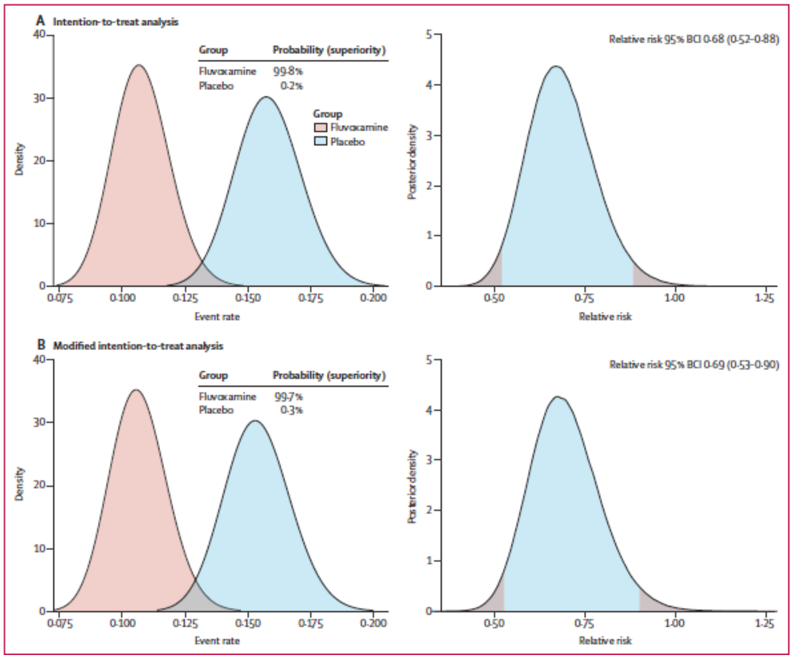

Figure 7. Probability of efficacy for the primary outcome in the ITT and mITT populations of TOGETHER

20.4. Placebo Information

Refer to Section 17.4.

20.4.1. Formulation, Appearance, Packaging, and Labeling

Refer to Section 17.4.1.

20.4.2. Drug Dispensing, Storage, and Stability

Refer to Section 17.4.2.

20.4.3. Dosing and Administration

Participants will self-administer one placebo tablet orally BID for one day, followed by two placebo tablets orally BID (four total per day) for 12 days, for a total of 13 days of placebo tablets.

20.5. Events of Special Interest

Suicidal ideation and behavior will be assessed at Day 7 and Day 14 via the Columbia-Suicide Severity Rating Scale (C-SSRS) Screener Since Last Asked Self-Report. The C-SSRS asks a total of up to six questions and a minimum of three questions. Sites will follow local policies in response to high risk screen cases. In addition, high risk screen cases will be advised to dial 988 to access the 988 Suicide & Crisis Lifeline.

21. Appendix F – Montelukast

21.1. Risk Assessment

The most common adverse reactions that occurred at a greater frequency in montelukast than in placebo and at an incidence rate ≥5% include upper respiratory infection, fever, headache, pharyngitis, cough, abdominal pain, diarrhea, otitis media, influenza, rhinorrhea, sinusitis, and otitis.

Post-market reports of neuropsychiatric events associated with montelukast have included agitation, hostility, anxiousness, depression, sleepwalking, suicidal thinking/behavior, and tremors.

Table 9. Montelukast sodium adverse reactions occurring at a higher incidence than placebo, in ≥1% of adults and adolescents ≥ 15 years of age.

Adverse Reaction	Montelukast sodium 10 mg/day, n=1955 (%)	Placebo, n=1180 (%)
Pain, abdominal	2.9	2.5
Asthenia/fatigue	1.8	1.2
Fever	1.5	0.9
Trauma	1.0	0.8
Dyspepsia	2.1	1.1
Pain, dental	1.7	1.0
Gastroenteritis, infectious	1.5	0.5
Headache	18.4	18.1
Dizziness	1.9	1.4
Influenza	4.2	3.9
Cough	2.7	2.4
Congestion, nasal	1.6	1.3
Rash	1.6	1.2
ALT Increase	2.1	2.0
AST Increase	1.6	1.2

Pyuria	1.0	0.9

21.2. Additional Appendix-Level Exclusion Criteria

None

21.2.1. Precautions

Participants with known aspirin sensitivity should continue to avoid aspirin and other non-steroidal anti-inflammatory agents while taking montelukast. Care should be taken with the reduction of oral corticosteroid therapy while on montelukast as systemic eosinophilia, sometimes presenting with clinical features of vasculitis consistent with Churg-Strauss syndrome, has been reported.

21.3. Study Drug Information

The primary active ingredient in montelukast tablets is montelukast sodium, which is an orally active leukotriene receptor antagonist that inhibits the cysteinyl leukotriene CysLT1 receptor. It is indicated for prophylaxis and chronic treatment of asthma, acute prevention of exercise-induced bronchoconstriction, relief of seasonal allergic rhinitis, and relief of perennial allergic rhinitis in patients ≥ 15 years of age.

21.3.1. Formulation, Appearance, Packaging, and Labeling

Montelukast sodium is a white to off-white powder. The 10 mg montelukast tablets are beige, rounded square-shaped, biconvex, film-coated tablets, debossed “M10” on one side and plain on the other side. Each tablet contains 10.4 mg of montelukast sodium, which is equivalent to 10 mg montelukast, and additional inactive ingredients including microcelac 100, croscarmellose sodium, low substituted hydroxypropyl cellulose, and magnesium stearate. The film coat includes hypromellose, hydroxypropyl cellulose, titanium dioxide, polyethylene glycol 6000, iron oxide red, and iron oxide yellow. All packaging will be labeled to indicate that the product is for investigational use.

21.3.2. Drug Dispensing, Storage, and Stability

A US commercial supply of montelukast tablets will be supplied by Accord Healthcare Inc. and distributed by Belmar Pharmacy. Montelukast tablets should be stored at 25°C with excursions permitted from 15°C to 30°C, protected from moisture and light.

21.3.3. Dosing and Administration

Montelukast will be self-administered orally by each participant at a dose of 10 mg (1 tablet) once daily for 14 days.

21.3.4. Rationale for Selection of Dose

Preclinical Studies:

Montelukast is a potent cysteinyl leukotriene receptor antagonist with anti-inflammatory effects and has been proven to significantly suppress oxidative stress and cytokine production.

Pre-clinical *in vitro* studies demonstrate montelukast's ability to act as an anti-viral agent against RNA viruses such as Zika, Influenza A, and Hepatitis C, among others.[73] A virtual screening tool, using an in silico molecular docking analysis, was used to simulate the binding of montelukast to catalytically active sites within the SARS-CoV-2 main protease and RNA dependent RNA polymerase. The results indicated that the Protein-Ligand ANT System chemplp docking score of montelukast against the main protease was −105.71, and the RNA dependent RNA polymerase was −104.75. These docking scores suggest that montelukast is likely to dock to both the main protease and the RNA dependent RNA polymerase of SARS-CoV-2, therefore demonstrating the potential to inhibit the enzymatic activity of the proteins and subsequently disrupting the substrate binding site.[74]

Montelukast inhibits the signaling of NF-κB, such as interleukin-6,8,10, TNF-alpha, MCP-1, and other proinflammatory mediators, which may result in a corresponding reduction of proinflammatory mediators, thereby attenuating cytokine production and the cytokine storm.[75] Pulmonary and extra-pulmonary manifestations in COVID-19 are attributed to a direct effect of SARS-CoV-2 on expressed ACE2 receptors or indirectly through NF-κB dependent induction of a cytokine storm. Montelukast may ameliorate extra-pulmonary manifestations of COVID-19 either directly through blocking of cysteinyl leukotriene receptors in different organs or indirectly through inhibition of the NF-κB signaling pathway.[76]

In addition, 36.4% of patients with COVID-19 experience CNS involvement, and although the majority of these events include headache, anosmia, and dysgeusia; more serious events such as stroke, delirium, and seizures have been reported.[77] In a 6-week treatment of young (4 months) and old (20 months) rats with montelukast, a reduction of neuroinflammation was seen; elevation of hippocampal neurogenesis and improvement in learning and memory in old animals was seen. By using gene knockdown and knockout approaches, the authors demonstrated that the effect was mediated through inhibition of the GPR17 receptor.[78]

Clinical studies:

One published retrospective analysis of hospitalized patients with confirmed COVID-19 treated with or without montelukast (10 mg orally, once daily, at the discretion of the treating provider) included ninety-nine patients not previously on montelukast in the analysis (30 montelukast/62 non-montelukast). Inclusion required hospitalization for at least 3 days. Montelukast was started on day 1 of hospitalization and all patients but 1 were on hydroxychloroquine. Steroids were used by 16-23% of patients during the first 3 days of hospitalization and 33-40% of patients during the entire hospitalization. A univariate logistic regression revealed a lower risk of clinical deterioration in patients who were receiving montelukast, defined as any increase in ordinal scale from day 1 to day 3 of hospitalization, (OR, 0.23; P =.029); however, in a multivariable logistic regression, the results in patients 60 years or older demonstrated that montelukast was not significantly associated with a reduced risk of clinical deterioration (OR, 0.28; P =.058).[79]

A recent prospective, randomized, controlled, single-blinded, single-center study investigated standard of care treatment versus two doses of montelukast (10 mg/day or 20 mg/day) in addition to standard of care treatment in hospitalized patients with confirmed COVID-19 at the Erzurum Regional Training and Research Hospital in Turkey.[80] To be considered for enrollment, patients were required to have a PaO2/FiO2 ratio above 200 at admission and to be starting on standard of care treatment (per Turkish Ministry of Health this was favipiravir for a total of 5

days). The primary outcome was a composite of progression to ARDS and/or macrophage activation syndrome (MAS). MAS was assessed at Day 5 of treatment. Clinical outcomes of disease progression were captured during hospitalization. Based on an assumption of a 20% or greater reduction in the primary outcome, the sample size was determined to be total accrual of 180 participants enrolled (60 in each group). The two montelukast arms were combined for the clinical outcome analysis. The standard of care arm had more MAS or ARDS events (8 vs 3, p = 0.001) and mortality (4 vs 0) than the pooled active treatment arms. There was no adverse event reporting in this manuscript, and there are numerous weaknesses to the study design.

Although the preclinical montelukast data available for SARS-CoV-2 is quite provoking, data from randomized clinical trials is lacking and the efficacy of montelukast for the treatment of COVID-19 warrants further studies. Ongoing clinical trials with montelukast propose a range of doses for testing (10 mg orally daily for 28-60 days; 60 mg orally daily for 14 days), see **Table 10**. One completed observational study (NCT047145515) has not posted study results. The proposed dose (Section 21.3.3) of 10 mg orally daily for 14 days fills a gap for a shorter course of therapy at the FDA-approved dose for which comprehensive safety data exists.

Table 10. Ongoing clinical trials with montelukast

Study	Study Design	Proposed Montelukast Dose	NCT Identifier
The COvid-19 Symptom MOntelukast Trial (COSMO)	Phase 3, randomized, blinded, placebo-controlled trial with 600 participants	10 mg daily for 60 days	NCT04389411
Efficacy of Montelukast in Mild-moderate Respiratory Symptoms in Patients With Long-COVID-19: (E-SPERANZA)	Phase 3, double-blind, randomized, placebo-controlled trial with 284 participants	20 mg daily for 28 days	NCT04695704
A National, Multi-Center, Open-Label, Three-Arm, Phase II Study to Investigate the Effect of Montelukast Between Emergency Room Visits and Hospitalizations in COVID-19 Pneumonia in Comparison With Standard Treatment	Phase 2, 3 arm (montelukast, montelukast plus favicovir, and favicovir), open-label, randomized trial with 380 participants	Montelukast arm dosing: 6x10 mg montelukast daily for 14 days	NCT04718285

21.4. Placebo Information

The placebo will be a montelukast-matched placebo. The appearance and packaging will match that of the study drug (as described in Section 21.3.1).

21.4.1. Formulation, Appearance, Packaging, and Labeling

Placebo will match the appearance of montelukast tablets: beige, rounded square-shaped, biconvex, film-coated tablets, debossed “M10” on one side and plain on the other side. All packaging will be labeled to indicate that the product is for investigational use.

21.4.2. Drug Dispensing, Storage, and Stability

Placebo will be supplied by Thermo Fisher Scientific and distributed by Belmar Pharmacy. Placebo should be stored at controlled room temperature 25°C, excursions permitted from 15°C to 30°C.

21.4.3. Dosing and Administration

Participants will self-administer one placebo tablet orally daily for 14 days. Dosing and administration will occur according to Section 21.3.3 in order to maintain blinding.

21.5. Events of Special Interest

Neuropsychiatric events including agitation, hostility, anxiousness, depression, sleepwalking, suicidal thinking/behavior, and tremors.

ACTIV-6
COVID-19 Outpatient Randomized Trial to Evaluate Efficacy of Repurposed Medications

STATISTICAL ANALYSIS PLAN (SAP)

Funding Agency National Center for Advancing Translational Sciences

Date 2022-04-01

Version 5.0

Principal Investigator Adrian Hernandez, MD, MHS
Duke Clinical Research Institute
200 Morris St.
Durham, NC 27701
Phone: 919-668-7515
Email: adrian.hernandez@duke.edu

Co-Principal Investigator/IND Sponsor: Susanna Naggie, MD, MHS
Duke Clinical Research Institute
200 Morris St.
Durham, NC 27701
Phone: 919-684-2584
Email: susanna.naggie@duke.edu

Data Coordinating Center Chris Lindsell, PhD
Vanderbilt University School of Medicine
2525 West End Avenue
Suite 1100, Rm 11129
Nashville, TN 37203
Phone: 615-343-9867
Email: chris.lindsell@vumc.org

Statisticians Thomas G. Stewart, PhD (DCC)
Frank Harrell, PhD (DCC)

Approval Signatures

By signing below, I indicate that I have reviewed this document in its entirety and approve its contents.

Statistician:

Thomas G. Stewart, PhD — 2022-04-01 — Approval Date

Principle Investigator:

Adrian Hernandez, MD, MHS — 4/8/2022 — Approval Date

Co-Principal Investigator/IND Sponsor:

Susanna Naggie, MD, MHS — April 15 2022 — Approval Date

Contents

1 Summary 5
2 Schedule of planned analyses 6
3 Analysis plan for the primary endpoint 6
3.1 Time to recovery (within 28 days) 6
3.1.1 Definition 6
3.1.2 Estimand 6
3.1.3 Model 6
3.1.4 Priors 7
3.2 All cause hospitalization or death (within 28 days) 7
3.2.1 Definition 7
3.2.2 Estimand 7
3.2.3 Model 7
3.3 Missing data 7
3.4 Heterogeneity of treatment effect 7
4 Analysis of screening endpoint 8
4.1 Concordance-, discordance-, equivalence- probabilities 8
4.2 Days Benefit 8
4.3 Model 9
4.3.1 Effect of previous state and time 10
4.3.2 Constraints and number of parameters 12
4.4 Method of inference 12
4.5 Likelihood 12
4.6 Priors 13
4.7 Decision thresholds 13
4.7.1 Guidance to proceed to evaluation of primary endpoint 13
4.7.2 Futility 13
4.8 Missing outcome data 14
4.9 Covariate adjustment 14
5 Model diagnostics and sensitivity analyses 14
6 Analysis plan for secondary endpoints 15
6.1 Schedule of planned analyses 15
6.2 Definitions and analysis details 15

6.2.1 Mean time unwell 15
6.2.2 Hospitalization or death (Day 14 and Day 28) 16
6.2.3 Mortality (Day 28) 16
6.2.4 Hospitalization, urgent care, emergency room visit, or death (Day 28) 16
6.2.5 Symptom Count (Day 14) 16
6.2.6 COVID Clinical Progression Scale (Day 7, Day 14, and Day 28) 16
6.2.7 Modified Patient-Reported Outcomes Measurement Information System (PROMIS)-29 (Day 7, Day 14, Day 28, and Day 90) 17
7 Trial characteristics 17
7.1 Simulation Study 17
7.1.1 Constructing a data generation model for time to event 17
7.1.2 Summary of design choices and decision thresholds 18
7.2 Type I error control and power 18
7.3 Supplementary details of simulation 19
7.3.1 The primary estimand 19
7.3.2 The estimation procedure 19
7.3.3 Posterior distribution of the treatment effect 19
7.3.4 The second approximation 20
7.3.5 How good is the approximation? 20
7.3.6 Calibration of type I error 24
7.3.7 Power 25

1 Summary

The key features of the trial are summarized in detail in the study protocol. The following table restates some of the key study features from the protocol.

**Feature**	**Description**
Study Design	Multicenter, blinded, placebo-controlled randomized clinical trial
Sample Size	A maximum of 1200
Primary Aim	To evaluate the effectiveness of repurposed medications [study drug(s)] in reducing symptoms of non-hospitalized participants with mild to moderate COVID-19
Primary Outcome	Appendix specific. Either (a) time to recovery or the composite endpoint of (b) hospitalization or death
Primary Estimand	Appendix specific.
Secondary Outcomes	• Mean time unwell<br>• Hospitalization or death (Day 14 and Day 28), if not primary<br>• Time to recovery (within 28 days), if not primary<br>• Mortality (Day 28)<br>• Hospitalization, urgent care, emergency room visit, or death (Day 28)<br>• COVID Clinical Progression Scale (Day 7, Day 14, and Day 28)<br>• Modified Patient-Reported Outcomes Measurement Information System (PROMIS)-29 (Day 7, Day 14, Day 28, and Day 90)
Inference approach	Bayesian posterior probabilities, point estimates, and credible intervals
Planned screening analyses	N = 300 and possibly 600
Screening endpoint guidance quantity	$P(screening\ endpoint\ at\ N = 1200 > 0)$
Planned interim analyses	If screening endpoint met, primary endpoint interim analysis at possibly N=300, 600, 900
Planned interim rules	Futility: posterior predictive $P(\text{primary endpoint efficacy at N} = 1200) < 0.05$<br>Efficacy: posterior P(primary endpoint efficacy) > 0.95
Type I error control	The combination of the screening endpoint, primary endpoint efficacy and futility rules, schedule of interim analyses, and choice of priors result in a simulated type I error rate of 0.05
Power	The minimal detectable effect at 80% power is a time to recovery hazard ratio of 1.2.

Study populations	• Modified Intention to Treat (mITT) defined as all participants who receive study drug/placebo.<br>• Safety population defined as all participants in the mITT population who report taking at least one dose of study drug or matching placebo

2 Schedule of planned analyses

Fixed enrollment triggers will be used for interim analyses. A screening analysis will occur after enrollment and completion of 14 day follow up of approximately 300 participants in a study arm (150 in study drug arm and 150 in placebo arm). If deemed appropriate based on the screening endpoint, a primary endpoint interim may occur immediately at N=300. Subsequent interim analyses may occur at N=600 or 900. If the study (appendix) has not terminated early for futility or efficacy at previous interim analyses, a final analysis will occur at N=1200. Individual study drugs may require different sample sizes, and the sample sizes may be adjusted based on the results of interim analyses.

3 Analysis plan for the primary endpoint

The primary endpoint is appendix specific. Possible endpoints include (a) time to recovery (within 28 days) or (b) hospitalization or death (within 28 days).

3.1 Time to recovery (within 28 days)

3.1.1 Definition

Time to recovery is defined in accordance with the sustained clinical recovery endpoint described in FDA guidance document for COVID-19 clinical trials (https://www.fda.gov/media/137926/download). Specifically, day of recovery is the first day on which the participant reports three consecutive days without COVID-19 symptoms.

3.1.2 Estimand

The Fine-Gray approach will be used to compare each intervention to placebo with respect to the cumulative incidence of recovery, accounting for the semi-competing risk of mortality (Fine and Gray 1999). The treatment effect parameter is estimated from a piece-wise proportional hazards regression model of the recovery endpoint in which subjects who die are retained in the risk set.

3.1.3 Model

The proportional hazards regression model will be constructed using a smooth, flexible parametric baseline hazard function. The model will be estimated within the Bayesian framework. The following covariates will be included in the regression component of the model:

1. Randomization assignment
2. Age as a restricted cubic spline with 3 knots
3. Gender
4. Duration of symptoms prior to treatment
5. Calendar time as restricted cubic spline with 4 knots
6. Vaccination status
7. Geographic region (Northeast, Midwest, South, West)
8. Call center indicator
9. Additional appendix-specific covariates relevant to baseline disease severity and patient risk.

3.1.4 Priors

The regression parameter for randomization assignment will be assigned a normal prior distribution with mean zero and standard deviation 0.1, selected so that type I error is bounded within 0.05. All other regression parameters will have a non-informative prior.

3.2 All cause hospitalization or death (within 28 days)

3.2.1 Definition

If a participant is hospitalized for any reason or dies from any cause within 28 days of receipt of study drug, then the participant will have experienced the all cause hospitalization or death endpoint.

3.2.2 Estimand

The difference in hospitalization or death rates will be summarized as an odds ratio estimated from a Bayesian logistic regression model.

3.2.3 Model

The Bayesian logistic regression model will include the following covariates:

1. Randomization assignment
2. Age as a restricted cubic spline with 3 knots
3. Gender
4. Duration of symptoms prior to treatment
5. Calendar time as restricted cubic spline with 4 knots
6. Vaccination status
7. Geographic region (Northeast, Midwest, South, West)
8. Call center indicator
9. Additional appendix-specific covariates relevant to baseline disease severity and patient risk.

3.3 Missing data

Despite efforts to accurately collect all baseline and outcome data, some missingness may occur. For time to recovery, participants who are lost to follow-up will by censored at the time of last follow-up.

Missing data in the covariates will be handled with imputation and posterior stacking. If the percentage of observations with missing covariates exceeds 5%, then multiple imputation (predictive mean matching) will be used. Otherwise, single imputation (conditional mean of the complete cases) will be used.

3.4 Heterogeneity of treatment effect

Heterogeneity of treatment effect will be evaluated for the following variables:

1. Vaccination status
2. Calendar time (pandemic epoch) as a restricted cubic spline with 3 knots
3. Duration of symptoms (time from symptom onset) as a restricted cubic spline with 3 knots
4. Age as a restricted cubic spline with 3 knots
5. BMI as a restricted cubic spline with 3 knots
6. Patient reported symptom severity (none, mild, moderate, severe)

Heterogeneity of treatment effect will be assessed by estimating model posterior probabilities for a full model including interaction term(s) with randomization assignment and a reduced model without the interaction term(s). The reduced model, M_r, is the primary endpoint regression model with the addition

of a variable in question (if not already in the model). For example, for vaccination status, two models are:

$$M_r: \quad \beta_1 \text{trt} + \beta_2 x_2 + \cdots + \beta_k x_k + \gamma_1 \text{vaccinated}$$

$$M_f: \quad \beta_1 \text{trt} + \cdots + \beta_k x_k + \gamma_1 \text{vaccinated} + \gamma_2 \text{trt} \times \text{vaccinated}$$

Model posterior probabilities, $P(M_f|y)$ and $P(M_r|y)$, will be estimated using non-skeptical, non-informative priors. Because the analyses for heterogeneity of treatment effect are exploratory, there are no decision thresholds for reportable findings.

4 Analysis of screening endpoint

4.1 Concordance-, discordance-, equivalence- probabilities

The OR and Wilcoxon test are translations of the treatment effect quantities known as concordance- and discordance- probabilities, which is why the Wilcoxon test can be performed by estimating a common OR fit with an ordinal regression model without covariates. In the setting of a treatment assignment variable, the concordance-, discordance-, and equivalence- probabilities are defined as a comparison of outcomes from a randomly selected subject in the intervention arm (denoted by A) and a similarly selected subject in the non-intervention arm (denoted by B):

$$\begin{aligned} \text{concordance} &= P(\text{intervention outcome better}) = P(Y_A < Y_B) \\ \text{discordance} &= P(\text{non-intervention outcome better}) = P(Y_A > Y_B) \\ \text{equivalence} &= P(\text{outcomes identical}) = P(Y_A = Y_B). \end{aligned}$$

4.2 Days Benefit

Days benefit is a summary measure of the treatment effect built on the concordance and discordance probabilities. As such, it is related to the common OR (and the Wilcoxon test), but it does not require the proportional odds assumption. It is a summary of treatment effect measured in days, which is an absolute and clinically relevant scale. The measure captures any degree of reduction in symptom burden or decreases in clinical event rates.

Days benefit is the cumulative difference of concordance and discordance probabilities over the course of follow-up. It is interpreted as a difference in days. Specifically, consider the outcome trajectory of study subjects assigned intervention to subjects assigned placebo, calculating (a) the expected number of days that a subject on treatment has a better outcome than placebo and (b) the expected number of days that the subject on placebo has a better outcome than intervention. The difference between the two counts is the days benefit.

To illustrate the definition, consider an hypothetical 14-day profile of outcome probabilities displayed in panels A and B in the figure below. For the sake of illustration, the outcome probabilities are different between the intervention and non-intervention arms. The colors in the profile plot denote distinct symptom and outcome scale values. At the bottom of the plot (in blue) is “no symptoms”. At the top (in red) is “death”. The intermediate scale values are ordered in between the extremes. The height of each box represents the probability of the corresponding outcome score. For example, on day 1, there is an approximate 0.18 probability that a patient in the intervention arm will report no symptoms.

The probabilities in panels A and B can be translated into concordance-, discordance-, and equivalence-probabilities, which are shown in panel C. Consider the comparison of a randomly selected patient in the intervention arm and a randomly selected patient in the non-intervention arm. Concordance occurs when

the outcome for the intervention arm patient is better than the outcome of the patient in the non-intervention. Discordance is the opposite event, and equivalence occurs when the outcome scores are equivalent between the two.

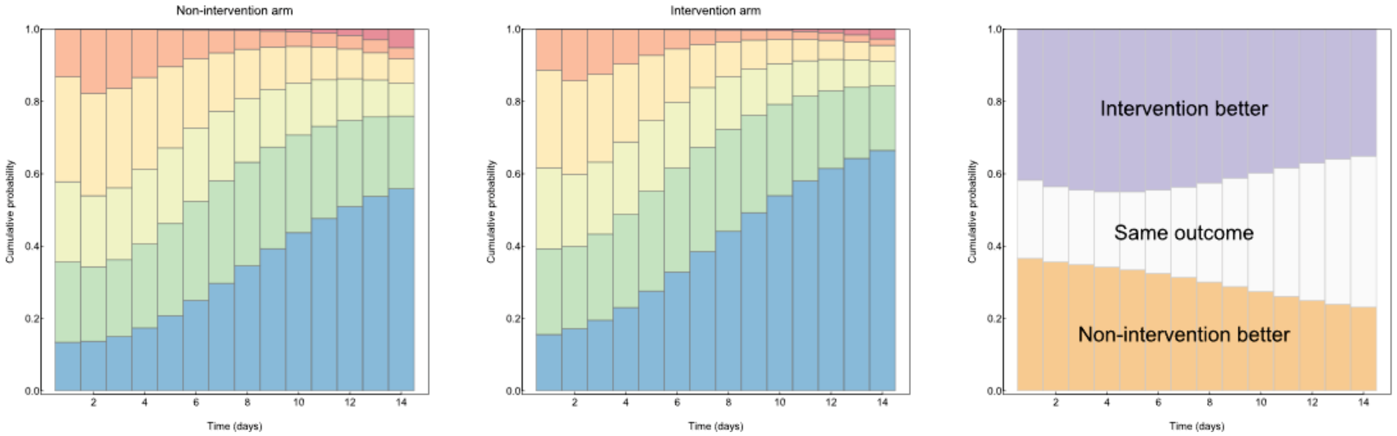

Figure 1. Illustration of Days Benefit

Days benefit, restated in terms of concordance and discordance probabilities, is the difference between (a) the probability that the intervention is better and (b) the probability that the non-intervention is better, summed over all the days of follow-up. In panel C, it is the area of the purple region minus the area of the orange region. Mathematically, it is expressed as

$$\text{days benefit} = \sum_{t=1}^{14} P(\text{intervention better}|t) - P(\text{non-intervention better}|t),$$

or equivalently,

$$\text{days benefit} = \text{follow-up time} \times [P(\text{intervention better}) - P(\text{non-intervention better})].$$

This quantity has the benefit of being measured in days, a scale that is readily understood and directly related to the practical impact of the treatment. An estimate of 3 days of benefit corresponds to a surplus of 3 days with better outcomes than the alternative treatment as captured by the primary endpoint.

The measure can also be modified to estimate how the treatment effect changes over time or over baseline symptom status. Estimates of expected benefit conditional on baseline covariate values can also be calculated, and the change in expected days benefit as a function of covariate values can be displayed in a partial effect plot.

4.3 Model

Consider a K category ordinal outcome assessed at T time points. Let Y denote the vector of random outcomes $Y_1, \ldots, Y_T$. The lower-case notation y and $y_1, \ldots, y_T$ denotes the observed values. Let trt indicate treatment assignment of intervention or placebo and let y_0 be the baseline symptom and outcome scale. The probability model for the outcome can be decomposed into conditional components:

$$\begin{aligned} P(Y = y_1, y_2, \ldots, y_T | y_0, \text{trt}) = {} & P(Y_T = y_T | y_{T-1}, y_{T-2}, \ldots, y_1, y_0, \text{trt}) \\ & \times P(Y_{T-1} = y_{T-1} | y_{T-2}, \ldots, y_1, y_0, \text{trt}) \cdots P(Y_3 = y_3 | y_2, y_1, y_0, \text{trt}) \\ & \times P(Y_2 = y_2 | y_1, y_0, \text{trt}) P(Y_1 = y_1 | y_0, \text{trt}) \end{aligned}$$

The model for analysis builds on three assumptions/modeling choices:

1. **Markov Property**. The Markov Property assumes that the conditional probability of today's outcome only depends on yesterday's outcome and not the entire trajectory of outcomes. Mathematically, this is expressed as $P(Y_t = y_t | y_{t-1}, y_{t-2}, \ldots, y_1, y_0, \text{trt}) = P(Y_t = y_t | y_{t-1}, \text{trt})$
2. **Smoothness in time and state**. The effect of treatment over time and the effect of the previous outcome (including the interaction of time and previous outcome) can be described adequately well with continuous functions like restricted cubic splines or polynomials in the cumulative log odds scale.
3. **Non-proportionality**. The model does not assume proportionality; however, it does contain non-proportionality parameters with skeptical priors.

We discuss each of these modeling choices in turn. First, the Markov assumption simplifies the expression of the probability model. The conditional probability components are only a function of the previous outcome and treatment assignment.

$$P(Y = y_1, y_2, \ldots, y_T | y_0, \text{trt}) = P(Y_T = y_T | y_{T-1}, \text{trt}) P(Y_{T-1} = y_{T-1} | y_{T-2}, \text{trt}) \cdots P(Y_1 = y_1 | y_0, \text{trt})$$

This is a common modeling choice when analyzing transition state outcomes, as it reduces the complexity of the model by reducing the number of model parameters.

Like the Markov assumption, the choice to impose smoothness on the effects of time and previous outcome state reduces the number of parameters to estimate. It allows the effect estimates to borrow information from adjacent time points and previous outcomes. This choice also has implications for missing outcomes, which is likely to be important in this trial as a substantial proportion of patients are unlikely to report an outcome for every day of follow-up.

The conditional probability components are modeled on the cumulative log odds scale, specifically

$$\log \frac{P(Y_t \geq k | y_{t-1}, \text{trt})}{1 - P(Y_t \geq k | y_{t-1}, \text{trt})} = \text{regression function}$$

Ignoring the longitudinal nature of the data for a moment, the simplest form of the model is

$$\text{for k} = 2, \ldots, \text{K}: \quad \log \frac{P(Y_t \geq k | \text{trt})}{1 - P(Y_t \geq k | \text{trt})} = \begin{cases} \alpha_k \text{ if trt } = 0 \\ \beta_k \text{ if trt } = 1 \end{cases}$$

$$\text{where } \alpha_2 \geq \alpha_3 \geq \cdots \geq \alpha_K$$
$$\text{and } \beta_2 \geq \beta_3 \geq \cdots \geq \beta_K$$

The α parameters relate to the estimated probabilities of the outcome states among subjects in the placebo arm (trt=0), and the β parameters capture the same for subjects in the intervention arm (trt =1). It is common to assume that $\alpha_2 - \beta_2 = \alpha_3 - \beta_3 = \cdots \alpha_K - \beta_K$ as a way to estimate a single common treatment effect. This assumption is known as the proportional odds assumption.

4.3.1 Effect of previous state and time

The above model, as currently written, does not include a term for either the previous state or time on treatment. The most flexible approach would be to specify a set of model parameters for each previous state and time point.

$$\text{for } k = 2, \ldots, K: \quad \log\frac{P(Y_t \geq k|\text{trt})}{1 - P(Y_t \geq k|\text{trt})} = \begin{cases} \alpha_{t,y_{t-1},k} \text{ if trt } = 0 \\ \beta_{t,y_{t-1},k} \text{ if trt } = 1 \end{cases}$$

$$\text{where } \alpha_{t,y_{t-1},2} \geq \alpha_{t,y_{t-1},3} \geq \cdots \geq \alpha_{t,y_{t-1},K}$$

$$\beta_{t,y_{t-1},2} \geq \beta_{t,y_{t-1},3} \geq \cdots \geq \beta_{t,y_{t-1},K}$$

If the number of possible outcome states is small, such a model may be appropriate. However, each additional outcome state increases the number of model parameters. When the number of states is moderate to large, modeling the log odds as a flexible function of the previous state, y_{t-1}, will cap the number of parameters and constrain the log odds to vary smoothly over the range of previous state values.

To capture the effect of the previous state in the model, smooth functions like polynomials or restricted cubic splines replace the constant terms. For the sake of notational ease, we used the notation $s(t, y_{t-1}, d)$ to denote a smooth function of d parameters capturing the effect of time and previous state. Note that because outcome state K is death, the transition probabilities are known when $y_{t-1} = K$.

$$\text{for } k = 2, \ldots, K \text{ and } y_{t-1} < K:$$

$$\log\frac{P(Y_t \geq k|y_{t-1},\text{trt})}{1 - P(Y_t \geq k|y_{t-1},\text{trt})} = \begin{cases} s_{\alpha_k}(t, y_{t-1}, d) \; if \; trt = 0 \\ s_{\beta_k}(t, y_{t-1}, d) \; if \; trt = 1 \end{cases}$$

$$\text{where } s_{\alpha_2}(t, y_{t-1}, d) \geq s_{\alpha_3}(t, y_{t-1}, d) \geq \cdots \geq s_{\alpha_K}(t, y_{t-1}, d)$$
$$\text{and } s_{\beta_2}(t, y_{t-1}, d) \geq s_{\beta_3}(t, y_{t-1}, d) \geq \cdots \geq s_{\beta_K}(t, y_{t-1}, d)$$

Were it not for the constraints imposed by the ordinal model, regression-type surfaces capturing the effects of time and previous state could be used directly for s_{α_k}or s_{β_k}. In was follows, we describe functions of time and previous state which include regression-like components which also conform to the constraints. Specifically, the regression components are constructed from the cross product of smooth basis functions. The quantity d represents the total number of parameters that define the surface. Note that $d = d_y \times d_t$, where d_y is the number or basis functions along the previous state dimension and d_t is the number along the time dimension. The quantities d_y and d_t need not be the same, as one dimension may warrant additional basis functions to capture more complex, nonlinear effects. If the individual basis functions are of the form

$$s(x) = \theta_1 + \theta_2 s_2(x) + \theta_3 s_3(x) + \cdots + \theta_d s_d(x),$$

then the cross product of the basis functions, say s^{t} for time and s^{p} for previous state, is

$$\sum_{i=1}^{d_y} \sum_{j=1}^{d_t} \phi_{i,j} \, s_i^p(y_{t-1}) s_j^t(t).$$

Each surface is defined sequentially, starting with s_{α_2}(or s_{β_2}).

$$s_{\alpha_2}(t, y_{t-1}, d) = \sum \sum \phi_{i,j}^{\alpha_2} s_i^p(y_{t-1}) s_j^t(t)$$

$$s_{\alpha_3}(t, y_{t-1}, d) = s_{\alpha_2}(t, y_{t-1}, d) - exp \sum \sum \phi_{i,j}^{\alpha_3} s_i^p(y_{t-1}) s_j^t(t)$$

$$s_{\alpha_4}(t, y_{t-1}, d) = s_{\alpha_3}(t, y_{t-1}, d) - \exp \sum \sum \phi_{i,j}^{\alpha_4} s_i^p(y_{t-1}) s_j^t(t)$$

and, in general for s_{α_K}, k>2,

$$s_{\alpha_k}(t, y_{t-1}, d) = s_{\alpha_{k-1}}(t, y_{t-1}, d) - \exp \sum \sum \phi_{i,j}^{\alpha_k} s_i^p(y_{t-1}) s_j^t(t).$$

Defining the surfaces in this way ensure that $s_{\alpha_{k-1}}(t, y_{t-1}, d) \geq s_{\alpha_k}(t, y_{t-1}, d)$. The same construction holds for s_{β_k}.

4.3.2 Constraints and number of parameters

Without the smooth functional relationship over time and previous state, there would be $2T(K-1)^2$ parameters.

$$\underbrace{2}_{\text{trt groups}} \times \underbrace{T}_{\text{time points}} \times \underbrace{K-1}_{\text{previous states}} \times \underbrace{K-1}_{\text{intercepts}} = 2T(K-1)^2$$

With the smooth functional relationships described above, there are $2d(K-1)$ parameters.

$$\underbrace{2}_{\text{trt groups}} \times \underbrace{d}_{\substack{\text{time and} \\ \text{previous state surface}}} \times \underbrace{K-1}_{\text{intercepts}} = 2d(K-1)$$

The smallest possible model is with $d = 3$, though to allow for more flexible effect surfaces, d will be larger. Note that the parameters are highly constrained, and the degrees of freedom in the parameter space will be noticeably less.

4.4 Method of inference

Model parameters and subsequent summaries of treatment effect will be estimated with the Bayesian posterior distribution. The posterior distribution is the normalized product of the likelihood model implied by the model described above and a set of prior distributions (which will be specified in the next section). Point estimates will be generated as the mean of the marginal posterior distribution and interval estimates will be calculated from 95% highest posterior density limits.

4.5 Likelihood

The vector of model parameters $\phi = (\phi_\alpha, \phi_\beta)$ is a combination of parameters that define the smooth surfaces of the cumulative log odds intercepts for the non-intervention and intervention arms. Denoting $\gamma_{t,y_{t-1},y_t,\text{trt}} = \alpha_{t,y_{t-1},y_t}$ or β_{t,y_{t-1},y_t} as the generic intercept term, then the likelihood can be expressed as

$$P(Y = y_1, y_2, \ldots, y_T | y_0, \text{trt}) = \prod_{t=1}^{T} P(Y_t = y_t | y_{t-1}, t, \text{trt})$$

$$= \prod_{t=1}^{T} \begin{cases} \frac{1}{1+e^{-\gamma_{t,y_{t-1},K,trt}}} & if\ y_t = K \\ 1 - \frac{1}{1+e^{-\gamma_{t,y_{t-1},2,trt}}} & if\ y_t = 1 \\ \frac{1}{1+e^{-\gamma_{t,y_{t-1},y_t,trt}}} - \frac{1}{1+e^{-\gamma_{t+1,y_{t-1},y_t+1,trt}}} & otherwise \end{cases}$$

The overall log likelihood is the log of the product, resulting in a summation of the logged likelihood contribution of all subjects.

4.6 Priors

The quantities of inferential interest are not the raw parameters that characterize the smooth surfaces that capture the effects of time and previous state on the cumulative log odds intercepts. Rather, the quantities of interest are parameters derived from the raw parameters, such as the transition probabilities and the degree of non-proportionality in the treatment effect for any specific combination of time and previous state. As such, informative priors will be primarily placed on derived quantities to reflect prior beliefs about non-proportionality.

Specifically, the informative model priors are:

$$\text{non proportionality} \sim Exp(2) \text{ for each time and previous state}$$

The non-proportionality quantities are defined for each combination of time and previous state.

$$\text{Let } \Delta_{t,y_{t-1},k} = \alpha_{t,y_{t-1},k} - \beta_{t,y_{t-1},k},$$

$$\text{then non proportionality, } np_{t,y_{t-1}} = var\left(\Delta_{t,y_{t-1},2}, \Delta_{t,y_{t-1},3}, \cdots, \Delta_{t,y_{t-1},K}\right).$$

The non-informative priors:

$$\phi_{i,j}^{\gamma_k} \sim N(0,10), \text{ the regression parameters}$$

$$\text{days benefit} \sim N(0,4)$$

4.7 Decision thresholds

Decisions regarding screening are based on the posterior predictive distribution of expected days benefit. There is no formal decision threshold for inefficacy or harm because those findings are superseded by the futility rule.

4.7.1 Guidance to proceed to evaluation of primary endpoint

The $posterior\ predictive\ P(\text{days benefit at N=1200} > 0|\text{interim data})$ will be calculated as a guidance quantity. The trial has been calibrated so that the trial maintains the 0.05 bound on type I error regardless of how decision-makers choose to interpret the screening endpoint analysis.

4.7.2 Futility

A futility probability will be calculated at each interim analysis from the posterior predictive distribution. If the $posterior\ predictive$ P(concluding efficacy at N=1200|interim data) is sufficiently small, then decision-makers may terminate the trial early because it is unlikely that the study will demonstrate a positive benefit of treatment. Even if the futility information is disregarded, the type I error is still bounded above by 0.05.

4.8 Missing outcome data

All available symptom scale values within the 14 day follow-up period will be used in the analysis. Each subject will contribute to the likelihood calculation for each observed outcome. For example, if outcome values are observed for days 1, 3, and 12, then the likelihood contribution will be

$$P(Y_1 = y_1, Y_3 = y_3, Y_{12} = y_{12} | y_0, \text{trt}) = P(Y_{12} = y_{12} | y_3, \text{trt}) P(Y_3 = y_3 | y_1, \text{trt}) P(Y_1 = y_1 | y_0, \text{trt})$$

where the probabilities conditional on outcomes from more than a day prior are calculated using the Chapman-Kolmogorov equation for n-step transition probabilities:

$$P(Y_t = y_t | y_{t-r}) = \sum_{i_1=1}^{K} \sum_{i_2=1}^{K} \cdots \sum_{i_{r-1}=1}^{K} P(Y_t = y_t | y_{t-1} = i_1) P(Y_{t-1} = i_1 | y_{t-2} = i_2) \cdots P\left(Y_{t-(r-1)} = i_{r-1} | y_{t-r} = y_{t-r}\right)$$

4.9 Covariate adjustment

All the decision-making quantities are marginalized estimates from the covariate adjusted model. Specifically, for general covariates x_1 to x_p, the model is

$$\text{for k} = 2, \ldots, \text{K:} \quad \log \frac{P(Y_t \geq k | \text{trt})}{1 - P(Y_t \geq k | \text{trt})} = \theta_1 x_1 + \cdots + \theta_2 x_p + \begin{cases} \alpha_{t, y_{t-1}, k} \text{ if trt } = 0 \\ \beta_{t, y_{t-1}, k} \text{ if trt } = 1 \end{cases}$$

$$\text{where } \alpha_{t, y_{t-1}, 2} \geq \alpha_{t, y_{t-1}, 3} \geq \cdots \geq \alpha_{t, y_{t-1}, K}$$

$$\beta_{t, y_{t-1}, 2} \geq \beta_{t, y_{t-1}, 3} \geq \cdots \geq \beta_{t, y_{t-1}, K}$$

For example, the covariates will include:

1. Age
2. Gender
3. Duration of symptoms prior to treatment
4. Additional appendix specific covariates relevant to baseline disease severity and patient risk.

Note that the covariates are included in the model relying on a a proportional odds assumption.

Summary measures, particularly of the screening endpoint treatment effect, will be calculated by marginalizing over the observed distribution of covariate values. For example, expected days benefit would be calculated by averaging the conditional quantity over observed covariate combinations.

$$\text{Days benefit} = \frac{1}{N} \sum_{i=1}^{N} \text{Days benefit} \mid x_{i,1}, \cdots, x_{i,p}$$

5 Model diagnostics and sensitivity analyses

The standard suite of model diagnostics for Bayesian models will be implemented, including graphical and analytical checks of the adequacy of the posterior samples, the model specification, and its predictions.

Imputation, as an alternative to the complete data likelihood approach to missing data described in an earlier section, will be performed. Further, a tipping point analysis will be provided which will estimate the degree to which differential missingness could change the conclusions of the analysis.

6 Analysis plan for secondary endpoints

The following table provides a summary of the planned secondary endpoints, the associated estimands, and an analysis approach.

**Endpoint**	**Estimand**	**Analysis method**
Mean time unwell	Difference in means	Screening endpoint model
Hospitalization or death (Day 14 and Day 28)	Model based estimates of the treatment effect odds ratio<br><br>If the number of events is less than 30, a descriptive analysis will be performed.	Logistic regression
Mortality (Day 28)	Model based estimates of the treatment effect odds ratio<br><br>If the number of events is less than 30, a descriptive analysis will be performed.	Logistic regression
Mortality (time to event)	Model based estimates of the treatment effect hazard ratio<br><br>If the number of events is less than 30, a descriptive analysis will be performed.	Cox regression
Hospitalization, urgent care, emergency room visit, or death (time to event within 28 days)	Model based estimates of the treatment effect hazard ratio<br><br>If the number of events is less than 30, a descriptive analysis will be performed.	Cox regression
COVID Clinical Progression Scale (Day 7, Day 14, and Day 28)	Treatment effect odds ratio	Cumulative probability ordinal regression with logit link
Modified Patient-Reported Outcomes Measurement Information System (PROMIS)-29 (Day 7, Day 14, Day 28, and Day 90)	Treatment effect odds ratio	Cumulative probability ordinal regression with logit link

6.1 Schedule of planned analyses

Secondary endpoints will be analyzed as part of the final analysis.

6.2 Definitions and analysis details

6.2.1 Mean time unwell

Mean time unwell is the expected number of days during 14 day follow-up that a patient is unwell, as defined by a dichotomization of the primary endpoint. The summary measure will be reported for all

possible dichotomizations. Mean time unwell for each individual treatment arm is estimated from the primary endpoint model, as is the difference in mean time unwell. The resulting point estimate and 95% credible interval of the difference in means will be generated from the marginalized (over covariates) posterior distribution. The posterior probability that the difference in means exceeds zero will also be reported.

6.2.2 Hospitalization or death (Day 14 and Day 28)

All cause hospitalization or death is hospitalization or death for any reason within the specified time window.

A Bayesian logistic regression model with flat priors will model the time to hospitalization or death. The regression model will include treatment assignment, age as a restricted cubic spline with 3 knots, and other covariates included in the primary endpoint model. Model-based estimates of the odds of the outcome on or before day 14 and day 28 will be generated. The resulting point estimate of the odds ratio comparing non-intervention and intervention arms, 95% credible interval, and the probability that the odds ratio is less than 1 will be generated from the posterior distribution marginalized over observed covariates.

6.2.3 Mortality (Day 28)

Like the hospitalization or death endpoint, time to death or loss-to-follow-up will be recorded for each subject. A Cox proportional hazards regression with flat priors will model the time to death endpoint from which model-based estimates of the odds of death within 28 days will be calculated. The baseline hazard will be degree 5 M-spline function. The resulting point estimate of the odds ratio comparing cases to controls, 95% credible interval, and the posterior probability that the odds ratio is less than 1 will be reported.

6.2.4 Hospitalization, urgent care, emergency room visit, or death (Day 28)

Time to hospitalization, urgent care, or emergency room visit is the date of the first qualifying event. Subjects that do not experience the event are censored on the day of last follow-up. This endpoint is analyzed like the mortality endpoint.

6.2.5 Symptom Count (Day 14)

Day 14 symptom count is the number of symptoms reported on day 14. If symptom resolution occurred prior to day 14 and a symptom count is not available for day 14, then the value of this endpoint is 0. If symptom count on day 14 is not available, then the nearest reported symptom count within 3 days may be used.

6.2.6 COVID Clinical Progression Scale (Day 7, Day 14, and Day 28)

The COVID clinical progression scale is the most severe of the following outcomes during the respective study day.

1. No limitation of activities
2. Limitation of activities
3. Hospitalized, no oxygen therapy
4. Hospitalized, on oxygen by mask or nasal prongs
5. Hospitalized, on non-invasive ventilation or high-flow oxygen
6. Hospitalized, on intubation and mechanical ventilation
7. Hospitalized, on ventilation + additional organ support – pressors, RRT, ECMO
8. Death

This endpoint will be analyzed with a cumulative probability ordinal regression with logit link (also called an ordered logistic model). Flat priors will be used. The regression model will include treatment assignment, age as a restricted cubic spline with 3 knots, and other relevant covariates. The resulting point estimate of the odds ratio comparing cases to controls, 95% credible interval, and the probability that the odds ratio is less than 1 will be generated from the posterior distribution marginalized over observed covariates.

Unless positively identified as dead or hospitalized, subjects are assumed to be 2 or lower on the scale. Subjects that are missing the endpoint are included in the analysis as a partially observed endpoint. (The contribution to the likelihood is the sum of both probabilities.)

6.2.7 Modified Patient-Reported Outcomes Measurement Information System (PROMIS)-29 (Day 7, Day 14, Day 28, and Day 90)

The PROMIS-29 is set of domain-specific subscales. The overall score and individual subscale scores will be analyzed with a cumulative probability ordinal regression with logit link (also called an ordered logistic model). Flat priors will be used. The regression model will include treatment assignment, age as a restricted cubic spline with 3 knots, and other relevant covariates. The resulting point estimate of the odds ratio comparing cases to controls, 95% credible interval, and the probability that the odds ratio is less than 1 will be generated from the posterior distribution marginalized over observed covariates. Subjects with missing responses will be excluded from the analysis.

7 Trial characteristics

7.1 Simulation Study

Rather than mimic the analysis of the screening endpoint followed by the primary endpoint, the following simulation study considers the time to recovery endpoint as if it were evaluated at each possible interim analysis, N=300, 600, 900, and 1200. This scenario represents the analysis sequence with largest type I error. By bounding this scenario to a type I error rate of 0.05, the global type I error rate of the study will also be bounded by 0.05.

In addition to the conservativeness introduced by bounding the worst-case scenario, this approach is also conservative because it does not account for the screening phase or futility rules, both of which put downward pressure on the type I error rate.

7.1.1 Constructing a data generation model for time to event

A model of time to event was constructed using an approach similar to method of moments with the Weibull distribution. Specifically, the parameters of the Weibull distribution were selected so that the resulting mean recovery time was 15 days from symptom onset and the 75th percentile of recovery was 22 days. The resulting distribution matches commonly reported metrics for recovery and the recovery data already collected within ACTIV-6. The Weibull distribution with shape 1.8 and scale 18.4 serves as the data generation model (the baseline model) for the simulations that follow. The recovery outcomes for patients in the intervention arm were generated from the baseline model modified with an additional treatment effect parameter (log relative hazard) which altered the outcome profile relative to the non-intervention arm. By increasing or decreasing the treatment effect parameter (β), the outcome profile of subjects in the intervention arm was improved or degraded. The outcome profiles of the intervention and non-intervention arms were identical when the treatment effect parameter was set to zero. For the sake of

simulation, the treatment effect assumes a proportional hazards treatment effect, and the treatment effect hazard ratio is $HR = e^{\beta}$.

7.1.2 Summary of design choices and decision thresholds

There are a number of design choices and decision thresholds which can be adjusted to reduce the risk of type I error. The following table summarizes the choices made for this trial.

**Design Choice**	
Schedule of analyses	At N=300, 600, 900, 1200
Treatment effect summary	Hazard ratio for the treatment assignment variable
Efficacy definition	Hazard ratio exceeds zero
Efficacy threshold	Posterior probability of efficacy exceeds 0.95
Futility threshold	(not incorporated into simulation) Posterior predictive probability of efficacy at N=1200 is not more than 0.05.
Treatment effect prior distribution	Normal distribution with mean 0 and standard deviation 0.1.

7.2 Type I error control and power

Suppose the treatment effect HR is 1.5. In order to calculate the power in this setting, 5000 datasets were generated from the data generation model described above. Each was analyzed with the Bayesian model described in section 3.1.3, starting with the first 300 subjects, then 600, and so on until 1200 subjects. The first triggering event (if there was one) plus the corresponding sample size was recorded for each dataset. If no triggering event occurred by the final analysis, the dataset was said to have triggered the max sample size constraint. Thus, each dataset generated one of two possible outcomes: efficacy or max sample size. The value of the standard deviation of the prior for the treatment effect was selected so that type I error was capped at 0.05. Power was calculated as the proportion of datasets that generated an efficacy outcome. The following figure shows the power curve over a range of treatment effect sizes.

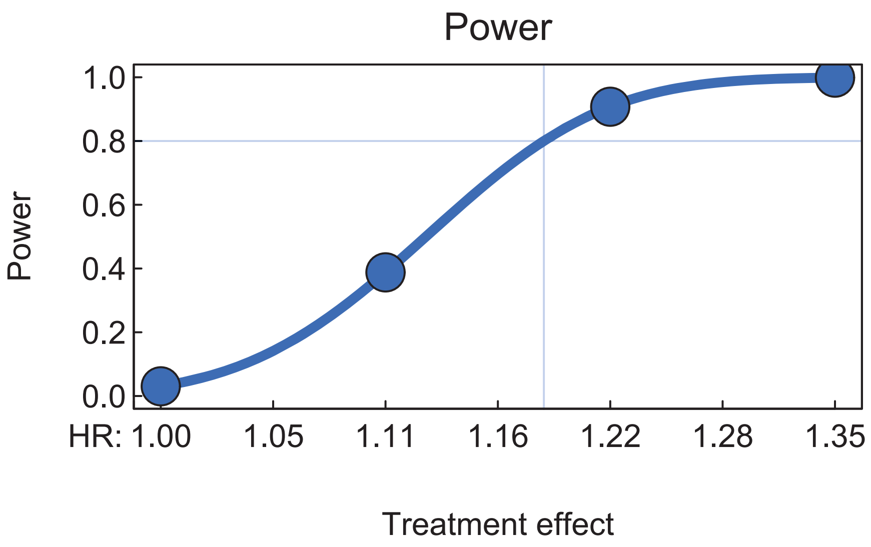

7.3 Supplementary details of simulation

7.3.1 The primary estimand

To summarize the differences between groups, we use the proportional hazards regression model. The transformed regression parameter $\exp(\beta_1)$ is the hazard ratio and is the primary quantity for inference.

$$\log \text{relative hazard} = \beta_1 \text{trt}$$

7.3.2 The estimation procedure

As has been described here, here, and here, the Cox model can be estimated from a Poisson generalized linear model. The dataset can be restructured so that the Poisson model generates identical estimates of the treatment effect as those achieved with direct estimation using the Cox model. That is, the restructured data combined with the following regression (where α_t denotes an estimate for each inter-event time interval) will generate identical estimates (and standard errors) of β_1.

$$\log(\lambda) = \alpha_t + \beta_1 x$$

Because β_1 is the quantity of primary interest, the estimation of several intercepts (α_t) is a hassle, especially when our end goal is a Bayesian posterior distribution. However, additional manipulation of the dataset will admit a much simpler Poisson GLM which results in approximate but highly accurate estimates of β_1. The intercept estimates are replaced with a single intercept (β_0) and an offset term, ($\log(z)$).

$$\log(\lambda) = \beta_0 + \beta_1 x + log(z)$$

For details about the connection between the Poisson GLM and the Cox model, see the documents linked above. For demonstration purposes, we show the estimates of β_1 from the Cox, Poisson GLM, and Poisson GLM with offset.

7.3.3 Posterior distribution of the treatment effect

The purpose of this document is to explain the derivation of the posterior distribution for β_1 in a Bayesian analysis. The jumping-off point is the Poisson GLM model with offset.

$$Y_i \sim \text{Poisson}(\lambda_i)$$

$$\log(\lambda_i) = \beta_0 + \beta_1 x_i + log(z_i)$$

The prior for the treatment effect is potentially informative, with variance θ. The prior for the intercept is an improper flat prior.

$$\beta_0 \sim \text{flat prior}$$

$$\beta_1 \sim N(0, \theta)$$

The log posterior is of the form

$$\beta_0 \sum_i y_i + \beta_1 \sum_i y_i x_i - \sum_i e^{\beta_0 + \beta_1 x_i + \log(z_i)} - \frac{1}{2\theta}\beta_1^2 + \text{constant.}$$

To integrate out β_0, note that the posterior can be expressed as

$$e^{A\beta_0} e^{-Be^{\beta_0}} C$$

With

$$A = \sum_i y_i$$
$$B = \sum_i e^{\beta_1 x_i + \log(z_i)}$$
$$\log C = \beta_1 \sum_i y_i x_i - \frac{1}{2\theta}\beta_1^2 + \text{constant}$$

and

$$\int_{-\infty}^{\infty} e^{A\beta_0}\, e^{-Be^{\beta_0}} C\, d\beta_0 = B^{-A}\Gamma(A)C$$

The log posterior of β_1 (denoted $\log f\,(\beta_1|x, y)$) is

$$-\left[\sum_i y_i\right] \log\left[\sum_i e^{\beta_1 x_i + \log(z_i)}\right] + \beta_1 \sum_i y_i x_i - \frac{1}{2\theta}\beta_1^2 + \text{new constant}$$

7.3.4 The second approximation

To approximate the posterior with a normal distribution, we use a Taylor series. Specifically,

$$\hat{\mu} = \text{root}\ \frac{d}{d\beta_1} \log f\,(\beta_1|x, y)$$

$$\hat{\theta} = -\left[\frac{d^2}{d\beta_1^2} \log f\,(\beta_1|x, y)\bigg|_{\hat{\mu}}\right]^{-1}.$$

Because the first and second derivatives of the log posterior are readily available, and the root is extremely easy to calculate, the computation time for estimating the posterior distribution is dramatically reduced compared to sampling approaches.

7.3.5 How good is the approximation?

First, we analyze a single dataset of increasing size. On the left of each plot, we show the Kaplan-Meier curves for the survival data; on the right we show the posterior function overlaid by the normal approximation. Because our expression for the posterior is only proportional to a distribution (that is, it isn't normalized to have unit area), we *normalize* the expression to have unit mode. Likewise, the same type of normalization was implemented for the normal approximation. In this example, a skeptical prior with variance 0.1 is used.

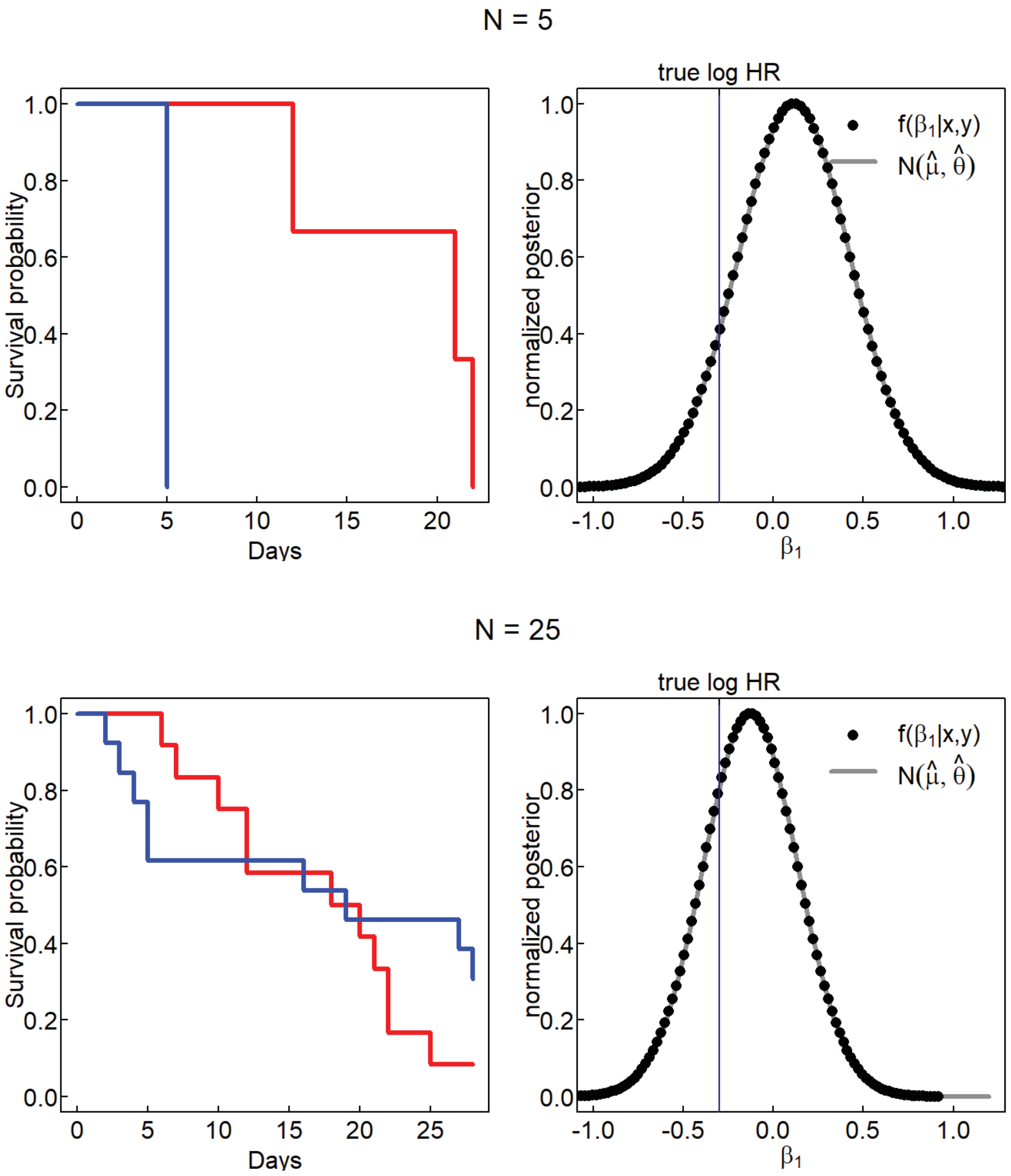
N = 5
true log HR
Survival probability
Days
normalized posterior
β_1
$f(\beta_1|x,y)$
$N(\hat{\mu}, \hat{\theta})$
N = 25
true log HR
Survival probability
Days
normalized posterior
β_1
$f(\beta_1|x,y)$
$N(\hat{\mu}, \hat{\theta})$

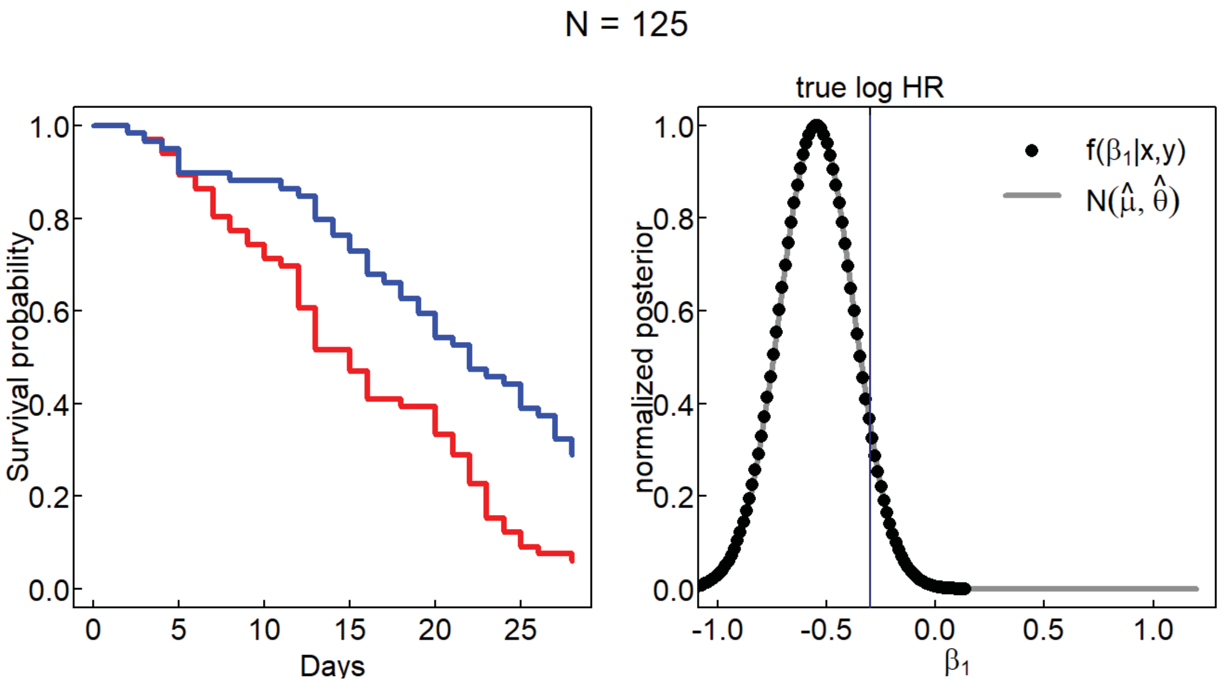
N = 125
Survival probability
1.0
0.8
0.6
0.4
0.2
0.0
0
5
10
15
20
25
Days
true log HR
normalized posterior
-1.0
-0.5
0.0
0.5
1.0
β_1
$f(\beta_1|x,y)$
$N(\hat{\mu}, \hat{\theta})$

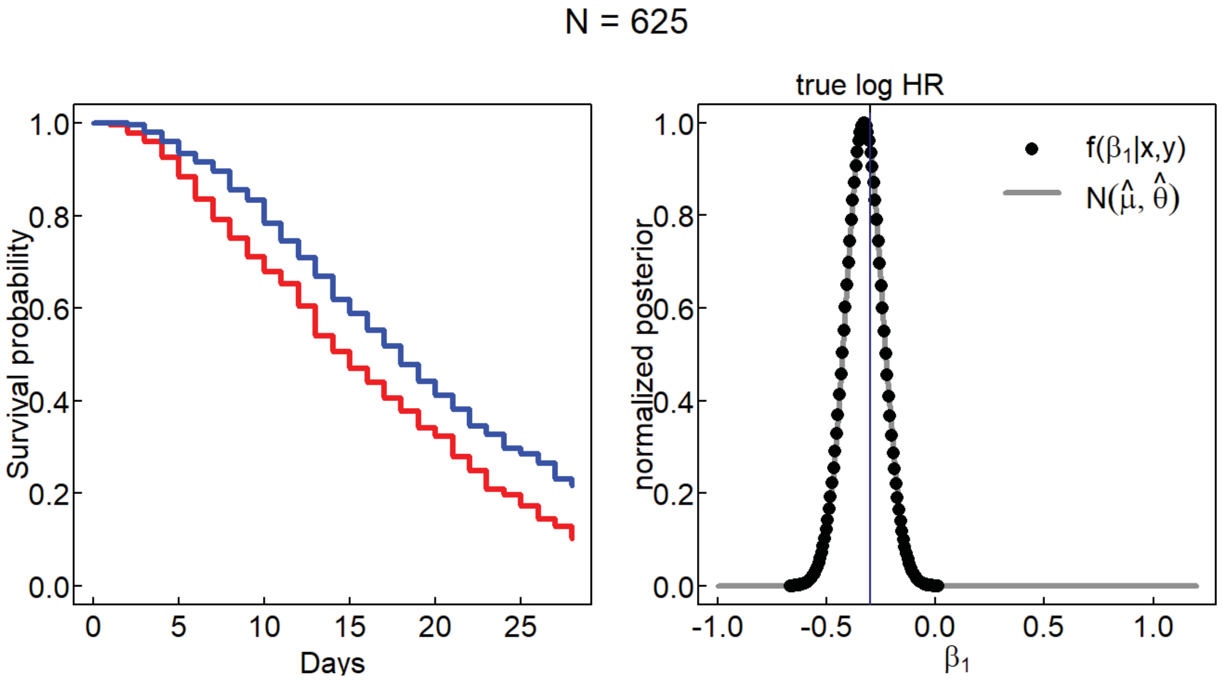
N = 625
Survival probability
1.0
0.8
0.6
0.4
0.2
0.0
0
5
10
15
20
25
Days
true log HR
normalized posterior
-1.0
-0.5
0.0
0.5
1.0
β_1
$f(\beta_1|x,y)$
$N(\hat{\mu}, \hat{\theta})$

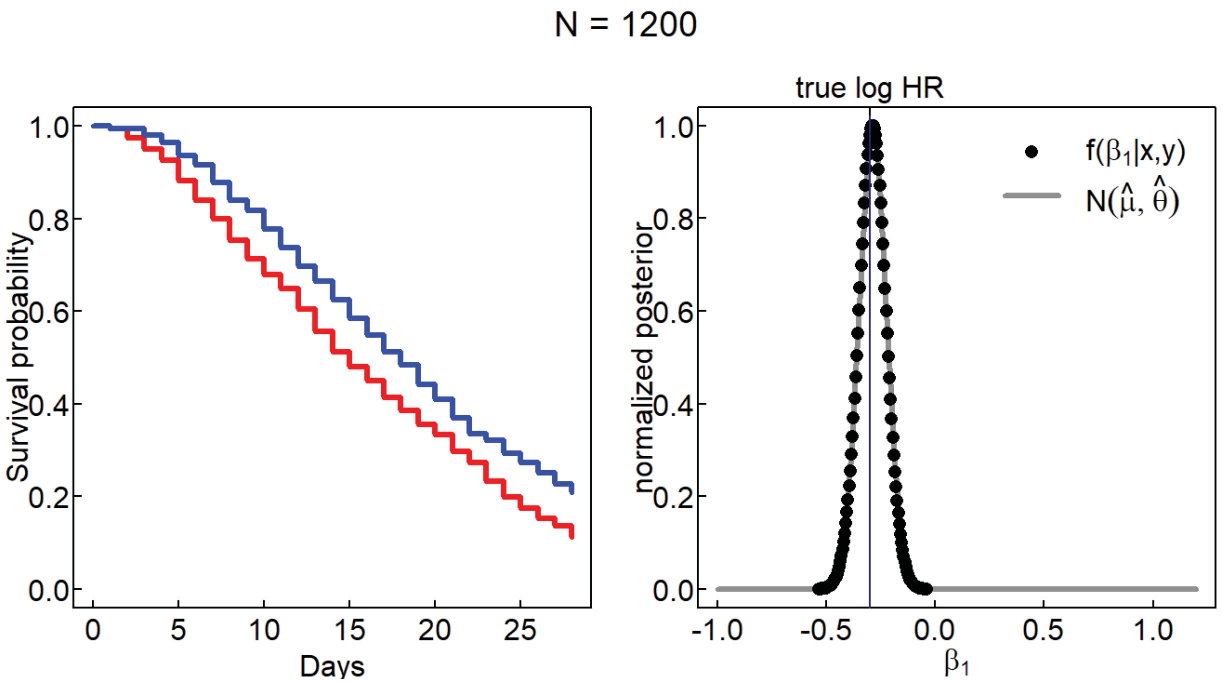

Second, we compare the approximate posterior solution to the `brms (R-package)` sampling solution. In the plot below, we have a QQ-type plot. The quantiles from the normal approximation are paired with the empirical quantiles of the 4000 posterior draws. The plot is generated for both N=300 and N=1200.

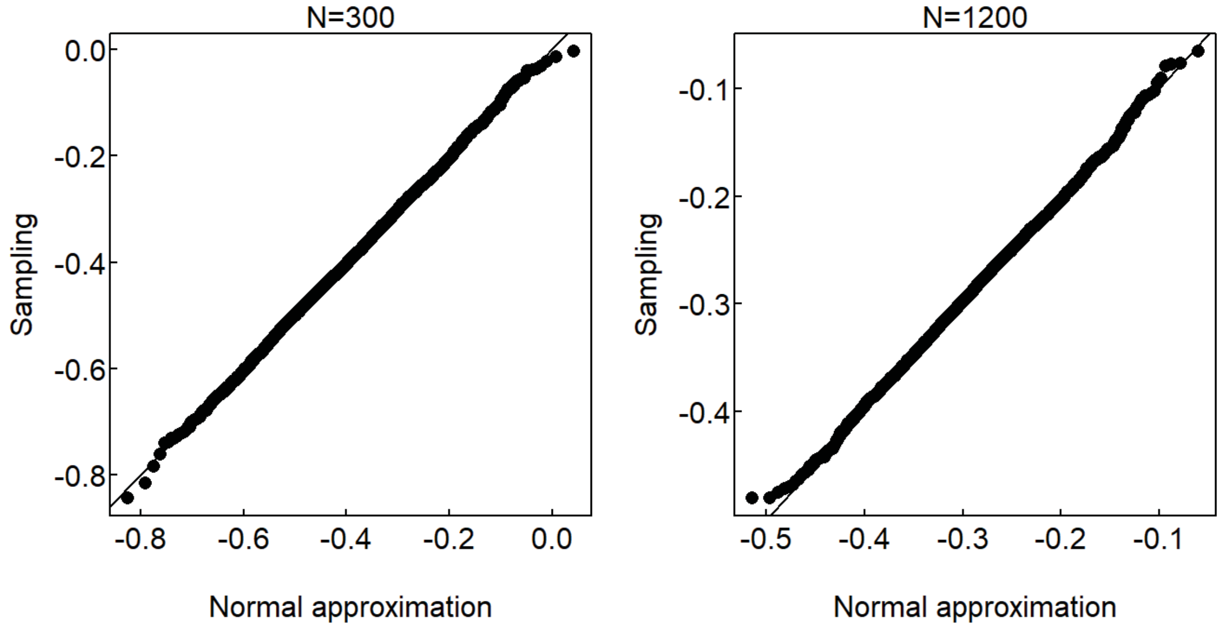

The approximate normal posterior distribution matches extremely well with the posterior distribution generated from sampling.

Third, we compare the posterior probabilities from the approximate solution to that of the sampling method in 25 datasets. Specifically, we calculate $P(\beta_1 > 0)$.

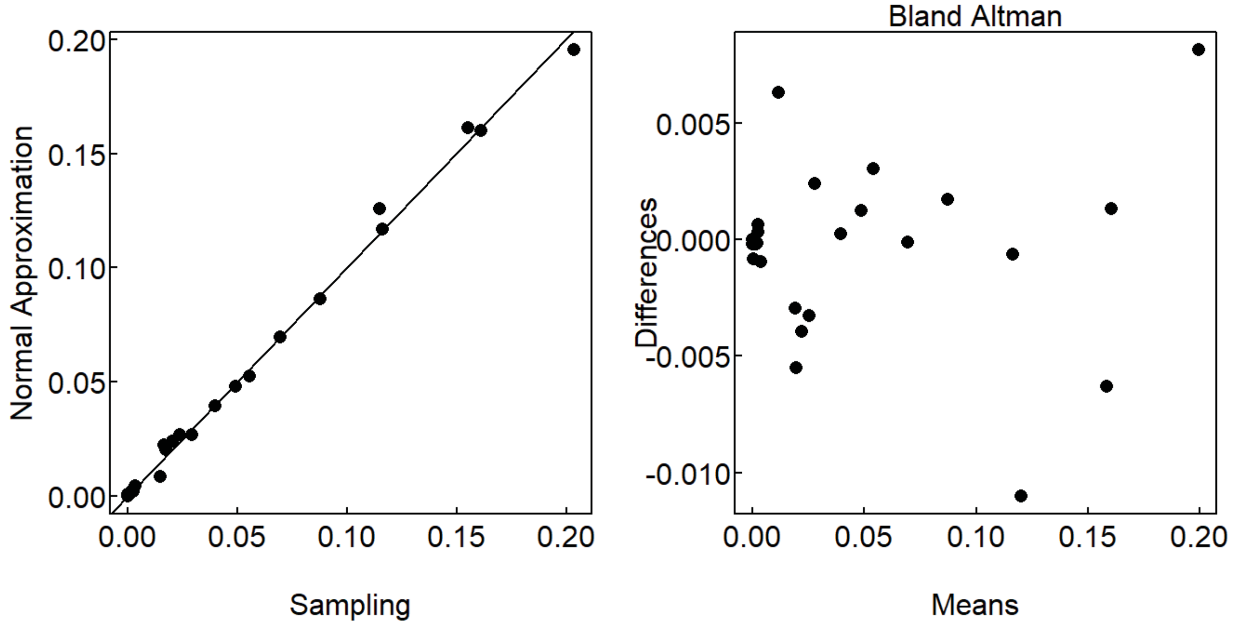

7.3.6 Calibration of type I error

In a trial with multiple looks, the variance of the treatment effect prior can be selected to achieve a desired type I error rate.

Simulated outcome data were generated from a Weibull distribution. Censoring occurred after 28 days. The resulting survival curve for the reference (placebo) arm is depicted in blue in the figure below. In red, we show the survival curves when the hazard ratio is 3/2 or 2/3. The reference survival curve was selected so that approximately 88% of subject experienced an event within 28 days.

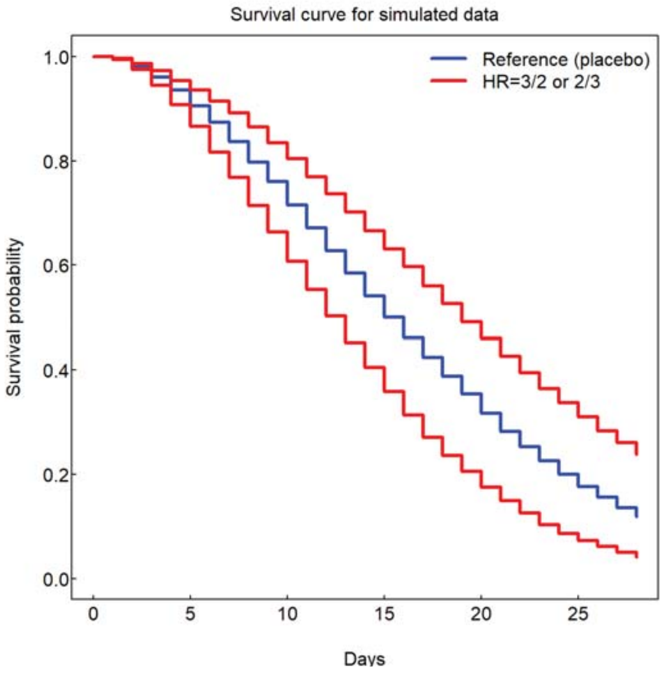

In the figure below, we show the results of a simulation study which indicate that a prior variance of 0.017 will achieve an error rate of 0.05. Note that this simulation study does not reflect a futility rule, which if implemented, may reduce the type I error rate and allow for a less skeptical prior.

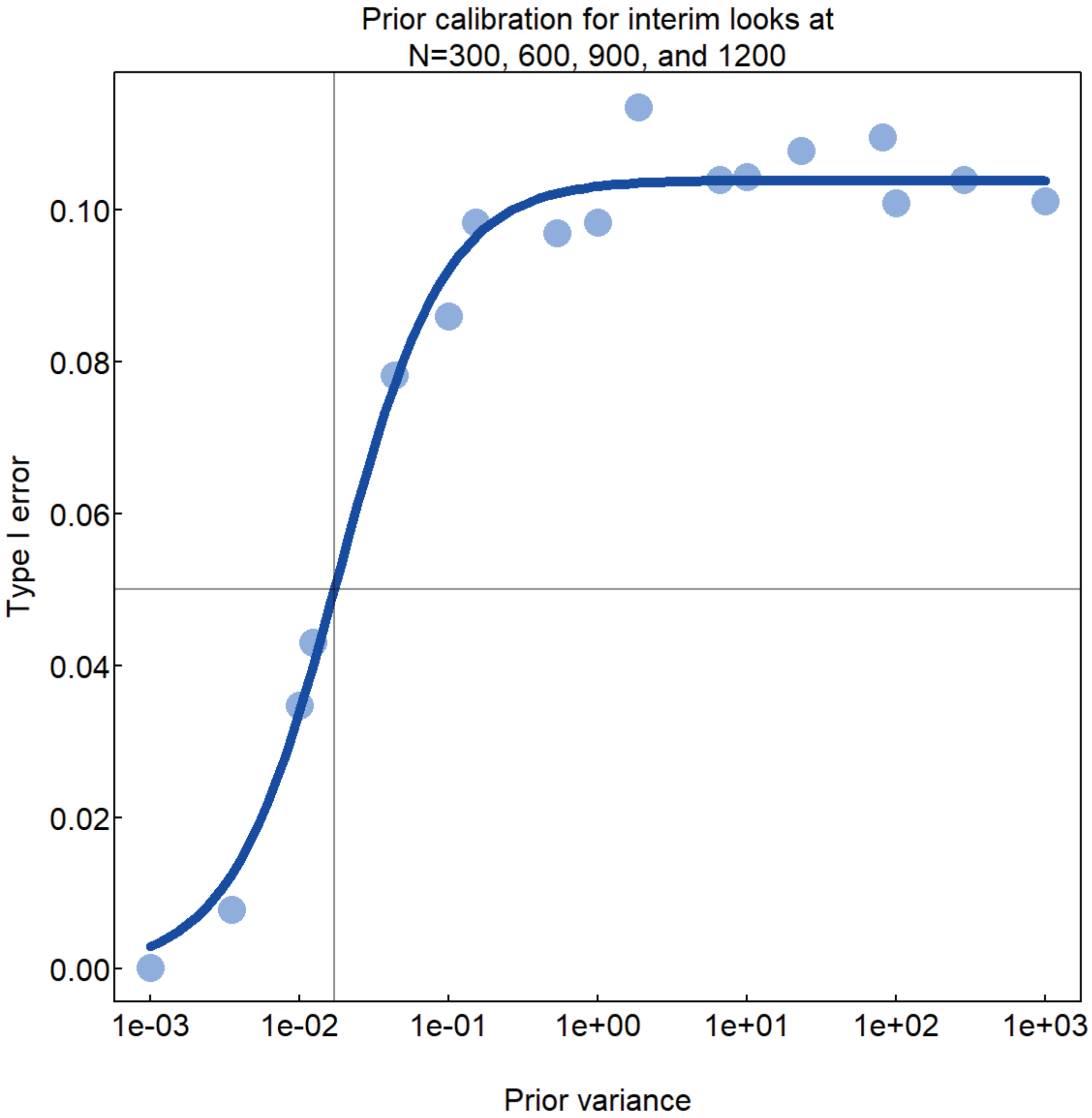

7.3.7 Power

Using the prior standard deviation identified in the calibration plot above, we perform a simulation study to get at the power of a trial with looks at 300, 600, 900, and 1200 subjects. The minimal detectable difference at 80% power is HR=1.17 when the data follow the distribution used in this simulation.

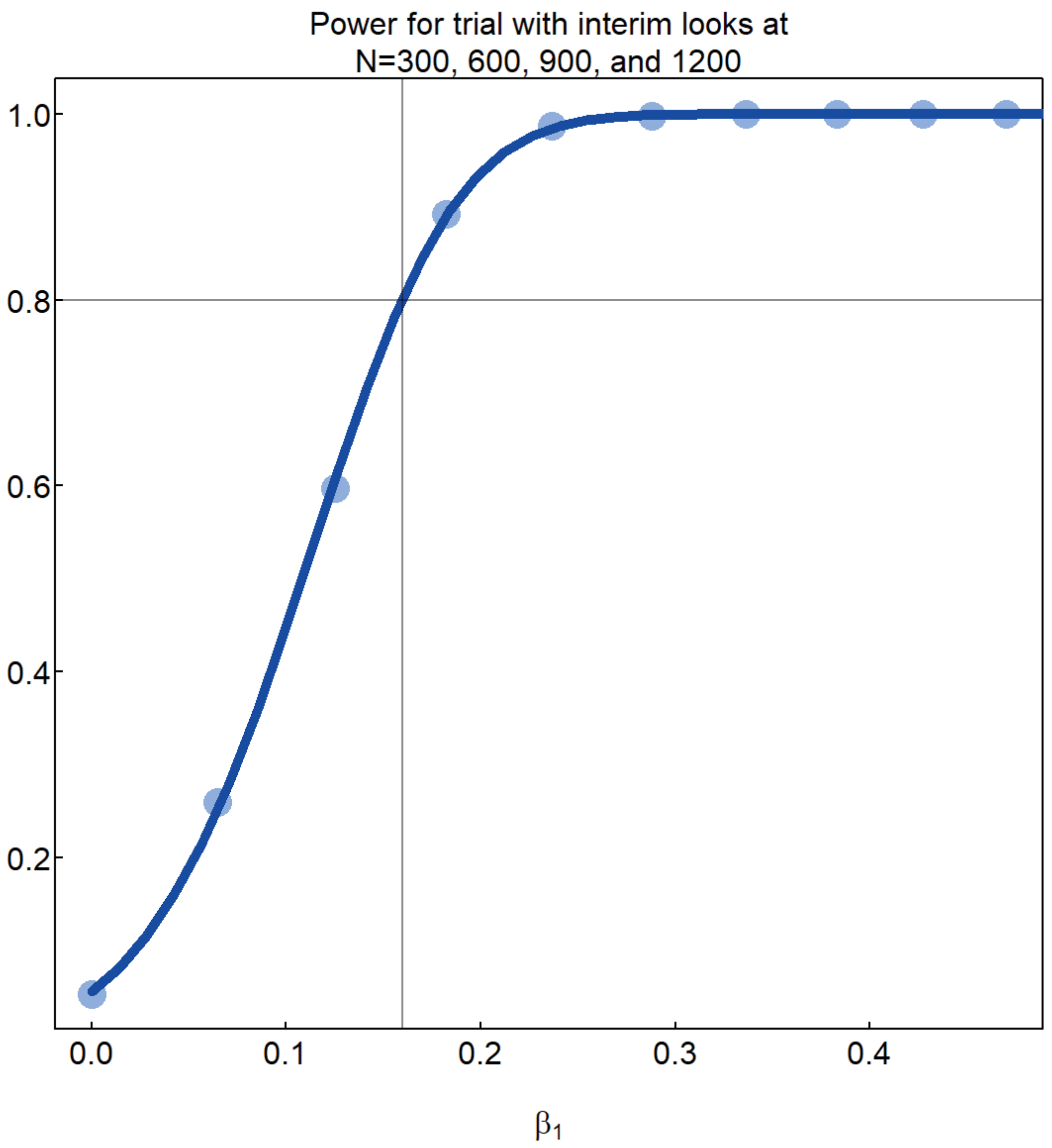
Power for trial with interim looks at
N=300, 600, 900, and 1200
1.0
0.8
0.6
0.4
0.2
0.0
0.1
0.2
0.3
0.4
β1
