# Supplementary material for: Time to Sustained Recovery Among Outpatients With COVID-19 Receiving Montelukast vs Placebo: The ACTIV-6 Randomized Clinical Trial
Source: JAMA Netw Open. 2024 Oct 18;7(10):e2439332. doi: 10.1001/jamanetworkopen.2024.39332 (PMC11581631; doi:10.1001/jamanetworkopen.2024.39332)
Supplement: Supplement 2. — eAppendix eMethods eTable 1. Baseline Symptom Prevalence and Severity eTable 2. Serious Adverse Events eFigure 1. Time From Symptom Onset to Receipt of Drug eFigure 2. Sensitivity Analysis of Hazard Ratio for Time to Sustained Recovery eFigure 3. All-Cause Hospitalization or Death for Montelukast Vs Placebo eFigure 4. All-Cause Hospitalization, Urgent Care, Emergency Department Visit, or Death for Montelukast Vs Placebo eFigure 5. Participants’ COVID-19 Clinical Progression Scale at Day 7, 14, and 28 eFigure 6. Kaplan-Meier Curves of Sustained Recovery by Onset of Symptoms and Symptom Severity eFigure 7. Heterogeneity of Treatment Effect Between Montelukast and Placebo for Time to Recovery eFigure 8. Posterior Distribution of Treatment Effect Adjusted for Site eFigure 9. Heterogeneity of Treatment Effect by Site eFigure 10. Posterior Densities of Primary End Point Model [file jamanetwopen-e2439332-s002.pdf]

Supplementary Online Content

Rothman RL, Stewart TG, Mourad A, et al; Accelerating COVID-19 Therapeutic Interventions and Vaccines–6 Study Group and Investigators. Time to sustained recovery among outpatients with COVID-19 receiving montelukast vs placebo: the ACTIV-6 randomized clinical trial. *JAMA Netw Open*. 2024;7(10):e2439332. doi:10.1001/jamanetworkopen.2024.39332

eAppendix

eMethods

eTable 1. Baseline Symptom Prevalence and Severity

eTable 2. Serious Adverse Events

eFigure 1. Time From Symptom Onset to Receipt of Drug

eFigure 2. Sensitivity Analysis of Hazard Ratio for Time to Sustained Recovery

eFigure 3. All-Cause Hospitalization or Death for Montelukast Versus Placebo

eFigure 4. All-Cause Hospitalization, Urgent Care, Emergency Department Visit, or Death for Montelukast Versus Placebo

eFigure 5. Participants' COVID-19 Clinical Progression Scale at Day 7, 14, and 28

eFigure 6. Kaplan-Meier Curves of Sustained Recovery by Onset of Symptoms and Symptom Severity

eFigure 7. Heterogeneity of Treatment Effect Between Montelukast and Placebo for Time to Recovery

eFigure 8. Posterior Distribution of Treatment Effect Adjusted for Site

eFigure 9. Heterogeneity of Treatment Effect by Site

eFigure 10. Posterior Densities of Primary End Point Model

This supplementary material has been provided by the authors to give readers additional information about their work.

© 2024 Rothman RL et al. *JAMA Network Open*.

eAppendix

ACTIV-6 Executive Committee

Adrian F. Hernandez, Duke Clinical Research Institute (Clinical Coordinating Center PI)

Susanna Naggie, Duke Clinical Research Institute (Clinical Coordinating Center Co-PI)

G. Michael Felker, Duke Clinical Research Institute (Medical Monitor, blinded)

Allison DeLong, Duke Clinical Research Institute

Rhonda Wilder, Duke Clinical Research Institute

Christopher J. Lindsell, Duke Clinical Research Institute (Data Coordinating Center Executive Director)

Russell L. Rothman, Vanderbilt University Medical Center (Data Coordinating Center PI)

Thomas G. Stewart, University of Virginia

Sean Collins, Vanderbilt University Medical Center

Sarah Dunsmore, National Center for Advancing Translational Sciences

Stacey Adam, Foundation for the National Institutes of Health

David R. Boulware, University of Minnesota

Elizabeth Shenkman, University of Florida

Florence Thicklin, Stakeholder Advisory Committee

Matthew William McCarthy, Weill Cornell Medicine, Stakeholder Advisory Committee

George Hanna, Biomedical Advanced Research and Development Authority

ACTIV-6 Protocol Oversight Committee

David Boulware, University of Minnesota, POC Co-Chair

Elizabeth Shenkman, University of Florida, POC Co-Chair

Adrian F. Hernandez, Duke Clinical Research Institute (Clinical Coordinating Center PI)

Susanna Naggie, Duke Clinical Research Institute (Clinical Coordinating Center co-PI)

G. Michael Felker, Duke Clinical Research Institute (Medical Monitor, blinded)

Allison DeLong, Duke Clinical Research Institute

Rhonda Wilder, Duke Clinical Research Institute

© 2024 Rothman RL et al. *JAMA Network Open*.

Christopher J. Lindsell, Duke Clinical Research Institute (Data Coordinating Center Executive Director)

Russell L. Rothman, Vanderbilt University Medical Center (Data Coordinating Center PI)

Thomas G. Stewart, University of Virginia

Sean Collins, Vanderbilt University Medical Center

Sarah Dunsmore, National Center for Advancing Translational Sciences

Stacey Adam, Foundation for the National Institutes of Health

Florence Thicklin, Stakeholder Advisory Committee

Matthew William McCarthy, Weill Cornell Medicine, Stakeholder Advisory Committee

George Hanna, Biomedical Advanced Research and Development Authority

Adit Ginde, University of Colorado Denver – Anschutz

Mario Castro, University of Kansas Medical Center

Dushyantha Jayaweera, University of Miami

Mark Sulkowski, John Hopkins University

Nina Gentile, Lewis Katz School of Medicine at Temple University

Kathleen McTigue, University of Pittsburgh Medical Center

Kim Marschhauser, PCORI

ACTIV-6 Clinical Trial Team

Adrian F. Hernandez, Duke Clinical Research Institute (Clinical Coordinating Center PI)

Susanna Naggie, Duke Clinical Research Institute (Clinical Coordinating Center Co-PI)

G. Michael Felker, Duke Clinical Research Institute (Medical Monitor, blinded)

Allison DeLong, Duke Clinical Research Institute

Rhonda Wilder, Duke Clinical Research Institute

Christopher J. Lindsell, Duke Clinical Research Institute (Data Coordinating Center Executive Director)

Russell L. Rothman, Vanderbilt University Medical Center (Data Coordinating Center PI)

Thomas G. Stewart, University of Virginia

© 2024 Rothman RL et al. *JAMA Network Open*.

ACTIV-6 Clinical Coordinating Center

Adrian F. Hernandez, Duke Clinical Research Institute (Clinical Coordinating Center PI)

Susanna Naggie, Duke Clinical Research Institute (Clinical Coordinating Center Co-PI)

G. Michael Felker, Duke Clinical Research Institute (Medical Monitor, blinded)

Allison DeLong, Duke Clinical Research Institute

Rhonda Wilder, Duke Clinical Research Institute

Ryan Fraser, Duke Clinical Research Institute

Mark Ward, Duke Clinical Research Institute

Jennifer Gamboa Jackman, Duke Clinical Research Institute

M. Patricia McAdams, Duke Clinical Research Institute

Julia Vail, Duke Clinical Research Institute

Kayla Korzekwinski, Duke Clinical Research Institute

Martina Oyelakin, Duke Clinical Research Institute

Julie Chopp, Duke Clinical Research Institute

Desmon Randle, Duke Clinical Research Institute

Samantha Dockery, Duke Clinical Research Institute

Rodney Adkins, Duke Clinical Research Institute

Mathew Crow, Duke Clinical Research Institute

Erin Nowell, Duke Clinical Research Institute

Kadie Wells, Duke Clinical Research Institute

Alicia Herbert, Duke Clinical Research Institute

Allegra Stone, Duke Clinical Research Institute

Heather Heavlin, Duke Clinical Research Institute

Linley Brown, Duke Clinical Research Institute

Tina Harding, Duke Clinical Research Institute

Amanda Harrington, Duke Clinical Research Institute

Meaghan Beauchaine, Duke Clinical Research Institute

Kelly Lindblom, Duke Clinical Research Institute

Andrea Burns, Duke Clinical Research Institute

Ahmad Mourad, Duke Clinical Research Institute

© 2024 Rothman RL et al. *JAMA Network Open*.

ACTIV-6 Independent Data Monitoring Committee

Voting Members:

Clyde Yancy, Northwestern University Feinberg School of Medicine (Chair)

Adaora Adimora, University of North Carolina, Chapel Hill (Vice-chair)

Susan Ellenberg, University of Pennsylvania

Kaleab Abebe, University of Pittsburgh

Arthur Kim, Massachusetts General Hospital

John D. Lantos, Children's Mercy Hospital

Jennifer Silvey-Cason, Participant representative

Statistical Data Analysis Center:

Frank Rockhold, Duke Clinical Research Institute (Lead faculty statistician)

Sean O'Brien, Duke Clinical Research Institute (Faculty statistician)

Frank Harrell, Vanderbilt University Medical Center (DCC-SDAC Faculty Liaison)

Zhen Huang, Duke Clinical Research Institute (Lead statistician)

ACTIV-6 Clinical Events Classification Committee

Renato Lopes, (CEC PI)

Faculty Reviewers: W. Schuyler Jones, Antonio Gutierrez, Robert Harrison, David Kong, Robert McGarrah, Michelle Kelsey, Brad Kolls, Cina Sasannejad, Rajendra (Raj) Mehta, Thomas Povsic

Fellow Reviewers: Konstantin Krychtiuk, Alexander Graham, Mark Kittipibul

ACTIV-6 Data Coordinating Center

David Aamodt, Vanderbilt University Medical Center

Jess Collins, Vanderbilt University Medical Center

Sheri Dixon, Vanderbilt University Medical Center

Yue Gao, Vanderbilt University Medical Center

John Graves, Vanderbilt University Medical Center

James Grindstaff, Vanderbilt University Medical Center

© 2024 Rothman RL et al. *JAMA Network Open*.

Frank Harrell, Vanderbilt University Medical Center (DCC-SDAC Faculty Liaison)

Jessica Lai, Vanderbilt University Medical Center

Vicky Liao, Vanderbilt University Medical Center

Christopher J. Lindsell, Duke Clinical Research Institute (Data Coordinating Center Executive Director)

Itzel Lopez, Vanderbilt University Medical Center

Elizabeth Manis, Vanderbilt University Medical Center

Kalley Mankowski, Vanderbilt University Medical Center

Jessica Marlin, Vanderbilt University Medical Center

Alyssa Merkel, Vanderbilt University Medical Center

Sam Nwosu, Vanderbilt University Medical Center

Savannah Obregon, Vanderbilt University Medical Center

Dirk Orozco, Vanderbilt University Medical Center

Nelson Prato, Vanderbilt University Medical Center

Max Rohde, Vanderbilt University Medical Center

Russell Rothman, Vanderbilt University Medical Center (Data Coordinating Center PI)

Jana Shirey-Rice, Vanderbilt University Medical Center

Krista Vermillion, Vanderbilt University Medical Center

Jacob Smith, Vanderbilt University Medical Center

Thomas Stewart, Vanderbilt University Medical Center / University of Virginia (Lead statistician)

Hsi-nien Tan, Vanderbilt University Medical Center

Meghan Vance, Vanderbilt University Medical Center

Maria Weir, Vanderbilt University Medical Center

ACTIV-6 Site Investigators & Study Coordinators

Advanced Medical Care, Ltd: Ray Bianchi, Jen Premas

Ananda Medical Clinic: Madhu Gupta, Greg Karawan, Santia Lima, Carey Ziomek

Arena Medical Group: Joseph Arena, Sonaly DeAlmeida

Ascension St. John Anuj Malik, Jane Bryce, Sarah Swint

Assuta Family Medical Group APMC: Soroush Ramin, Jaya Nataraj

© 2024 Rothman RL et al. *JAMA Network Open*.

Boston Medical Center: Julien Dedier, Ricardo Cruz, Ana Maria Ramirez, Lori Henault

Brooke Army Medical Center (Geneva Foundation): Joseph Marcus, Alexis Southwell, Genice Jacques, Cedar Sexton

Chandler Regional Medical Center Brian Tiffany, Charlotte Tanner, Allegra Sahelian

Christ the King Health Care, P.C.: Constance George-Adebayo, Adeolu Adebayo

Christus Saint Frances Hospital: Jose Zapatero, Julie Clement

Christus St. Vincent Regional Medical Center: Theresa Ronan, Ashley Woods, Christopher Gallegos, Tamara Flys, Olivia Sloan

Clincept: Anthony Olofintuyi, Joshua Samraj, Jackelyn Samraj, Alma Vasbinder, Amaya Averett

Clinical Trials Center of Middle Tennessee: Alex Slandzicki, Jessica Wallan

Comprehensive Pain Management and Endocrinology: Claudia Vogel, Sebastian Munoz

David Kavtaradze MD, Inc.: David Kavtaradze, Casandra Watson

David Singleton MD, PA: David Singleton, Marcus Sevier, Maria Rivon

Del Pilar Medical and Urgent Care: Arnold Del Pilar, Amber Spangler

DHR Health Institute for Research: Sohail Rao, Luis Cantu

Diabetes and Endocrinology Assoc. of Stark County: Arvind Krishna, Heidi Daugherty, Brandi Kerr, Kathy Evans

Doctors Medical Group of Colorado Springs, P.C.: Robert Spees, Mailyn Marta

Duke University: G. Michael Felker, Meaghan Beauchaine, Amanda Harrington

Duke University Hospital: Rowena Dolor, Lorraine Vergara, Jackie Jordan

Elite Family Practice: Valencia Burruss, Terri Hurst

Emory University: Igho Ofotokun, Paulina A. Rebolledo, Cecilia Zhang, Jessica Traenkner, Mary M. Atha

Essentia Health: Rajesh Prabhu, Krystal Klicka, Amber Lightfeather

Essential Medical Care, Inc.: Vickie James, Marcella Rogers

Family Practice Doctors P.A.: Chukwuemeka Oragwu, Ngozi Oguego

First Care Medical Clinic: Rajesh Pillai, Santia Lima

Focus Clinical Research Solutions: Ahab Gabriel, Emad Ghaly, Marian Michal

Franciscan Health Michigan City: Michelle Vasquez, Angela Mamon, Michelle Sheets

G&S Medical Associates, LLC: Gammal Hassanien, Samah Ismail, Yehia Samir

© 2024 Rothman RL et al. *JAMA Network Open*.

George Washington University Hospital: Andrew Meltzer, Soroush Shahamatdar, Ryan S. Heidish, Aditya Loganathan

Geriatrics and Medical Associates: Scott Brehaut, Angelina Roche

GFC of Southeastern Michigan, PC: Manisha Mehta, Nicole Koppinger

Health Quality Primary Care: Jose Baez, Ivone Pagan

Highlands Medical Associates, P.A.: Dallal Abdelsayed, Mina Aziz

Hoag Memorial Hospital Presbyterian: Philip Robinson, Grace Lozinski, Julie Nguyen

HOPE Clinical Research: Alvin Griffin, Michael Morris, Nicole Love, Bonnie Mattox, Raykel Martin

Hugo Medical Clinic: Victoria Pardue, Teddy Rowland

Innovation Clinical Trials Inc.: Juan Ruiz-Unger, Lionel Reyes, Yadira Zamora, Navila Bacallao

Jackson Memorial Hospital: John Cienki

Jadestone Clinical Research, LLC: Jonathan Cohen, Ying Yuan, Jenny Li

Jeremy W. Szeto, D.O., P.A.: Jeremy Szeto

Johns Hopkins University: Mark Sulkowski, Lauren Stelmash, Sara Mekhael

L&A Morales Healthcare, Inc: Idania Garcia del Sol, Ledular Morales Castillo, Anya Gutierrez, Sabrina Prieto

Lakeland Regional Medical Center: Arch Amon, Andrew Barbera, Andrew Bugajski, Walter Wills, Kellcee Jacklin

Lamb Health, LLC: Deryl Lamb, Amron Harper

Lapis Clinical Research: Elmer Stout, Merischia Griffin

Lice Source Services Plantation: Nancy Pyram-Bernard, Arlen Quintero

Loyola University Medical Center: Nina Clark, Mary Barsanti-Sekhar, Christina Carbrera-Mendez, Mary Rose Evans

Lupus Foundation of Gainesville: Eftim Adhami, Giovanni Carrillo

Maria Medical Center, PLLC: Josette Maria, Diksha Paudel, Oksana Raymond

Medical Specialists of Knoxville: Jeffrey Summers, Tammy Turner

Medical University of South Carolina: Leslie Lenert, Ebony Panaccione, Elizabeth Szwast, Amy Reynolds

Mediversity Healthcare: Ahsan Abdulghani, Pravin Vasoya

Miller Family Practice, LLC: Conrad Miller, Hawa Wiley

© 2024 Rothman RL et al. *JAMA Network Open*.

Morehouse School of Medicine: Austin Chan, Saadia Khizer

North Shore University Health System/Evanston Hospital: Nirav Shah, Oluwadamilola Adeyemi, Wei Ning Chi, July Chen, Melissa Morton-Jost

Ochsner Clinic Foundation: Julie Castex, Ali Quirch

Olive View – UCLA Medical Center: Hrishikesh Belani, Rosario Machicado, Bjorn Bjornsson

Olivo Wellness Medical Center: Jacqueline Olivo, Maria Maldonado

Pine Ridge Family Medicine Inc.: Anthony Vecchiarelli, Diana Gaytan-Alvarez

Premier Health: Vijaya Cherukuri, Santia Lima

Providence Regional Medical Center: Radica Alicic, Allison A. Lambert, Carissa Urbat, Joni Baxter, Ann Cooper

Rapha Family Wellness: Dawn Linn, Laura Fisher

Raritan Bay Primary Care & Cardiology Associates: Vijay Patel, Yuti Patel, Roshan Talati, Priti Patel

Romancare Health Services: Leonard Ellison, Angee Roman, Jeffrey Harrison

Rush University Medical Center: James Moy, Dina Naquiallah

Spinal Pain and Medical Rehab, PC: Binod Shah, Santia Lima

Stanford University: Orlando Quintero, Jake Scott, Yasmin Jazayeri, Andrew O'Donnell, Divya Pathak

Sunshine Walk In Clinic: Anita Gupta, N. Chandrasekar

Superior Clinical Research: Clifford Curtis, Briana White, Martha Dockery

Tabitha B. Fortt, M.D., LLC: Tabitha Fortt, Anisa Fortt

Tallahassee Memorial Hospital: Ingrid Jones-Ince, Alix McKee

Tampa General Hospital: Jason Wilson, Jackie Marcelin, Brenda Farlow

Temple University Hospital: Nina Gentile, Casey Grady

Texas Health Physicians Group: Randall Richwine, Penny Pazier

Texas Tech University Health Sciences Center in El Paso: Edward Michelson, Susan Watts, Diluma Kariyawasam, Leann Rodriguez

The Angel Medical Research: Jose Luis Garcia, Ismarys Manresa, Angel A. Achong, Mari C. Garcia

The Heart and Medical Center: Sangeeta Khetpal, Faith Posey

Trident Health Center: Arvind Mahadevan, Santia Lima

TriHealth, Inc: Martin Gnoni

UF Health Precision Health Research: Carla VandeWeerd, Jeffrey Lowenkron, Erica Sappington, Mitchell Roberts

UMass Memorial Medical Center: Jennifer Wang, Melissa Adams, Xinyi Ding, Mary Co

University Diagnostics and Treatment Clinic: Mark D'Andrea, Mina Aziz

University Medical Center- New Orleans: Stephen Lim, Wayne Swink, Emily Bozant, Madeline Young

University of Arkansas: Michael Wilson, Carly Eastin, Allyson Cheathem, Ahad Nadeem, Crystal Walters

University of Cincinnati: Margaret Powers-Fletcher, Douglas Brown, Delia Miller, Sylvere Mukunzi

University of Florida Health: Elizabeth Shenkman, Brittney Manning, Melissa Terry-White, Maria Cristina Crizaldo

University of Florida-JAX-ASCENT: Carmen Isache, Jennifer Bowman, Angelique Callaghan-Brown, Debra Martin

University of Kansas – Wichita: Tiffany Schwasinger-Schmidt, Ashley Ast, Brent Duran, Ashlie Cornejo, Allie Archer

University of Miami: Dushyantha Jayaweera, Maria Almanzar, Vanessa Motel

University of Minnesota: Matt Pullen, Blake Anderson, Neeta Bhat, Daniela Parra, Paula Campora

University of Missouri – Columbia: Matthew Robinson, Michelle Seithel, Liz Kendrick, Dyann Helming, Kelly Pollock

University of Pittsburgh: Akira Sekikawa, Emily Klawson, Jonathan Arnold, Nathan Weiland

University of Texas Health Science Center at Houston: Luis Ostrosky-Zeichner, Bela Patel, Virginia Umana, Laura Nielsen, Carolyn Z. Grimes

University of Texas Health Science Center at San Antonio: Thomas F. Patterson, Robin Tragus, Bridgette T. Soileau

University of Texas Rio Grande Valley: Timothy Heath, Erik Hinjosa, Cesar Gutierrez

University of Virginia Health System: Patrick E. H. Jackson, Caroline Hallowell, Heather M. Haughey

Vaidya MD PLLC: Bhavna Vaidya-Tank, Cameron Gould

Vanderbilt University Medical Center: Parul Goyal, Sue Sommers, Haley Pangburn, Carly Jones, Lori Michalowski

Wake Forest University Health Sciences: John Williamson, Brittany Wortham, Rica Abbott,

Weill Cornell Medical College: Matthew McCarthy, Unwana Umana, Candace Alleyne, Britta Witting

© 2024 Rothman RL et al. *JAMA Network Open*.

Well Pharma Medical Research: Eddie Armas, Ramon O. Perez Landaburo, Michelle De La Cruz, Martha Ballmajo, Jorge Alvarez

© 2024 Rothman RL et al. *JAMA Network Open*.

eMethods

Participant Monitoring

The daily and follow-up assessments were monitored, and sites were actively notified of events requiring review, including serious adverse events (SAEs). In addition, participants were invited during assessments to request contact from the study team or to report any unusual circumstances. Failure to complete daily assessments also triggered a review for any possible SAEs. A missed assessment on the day after receiving the first dose of study medication (day 2) or any day of missed assessments up to day 14 prompted a notification to the site to contact the participant. All participants were instructed to self-report concerns either via an online event reporting system, by calling the site, or by calling a 24-hour hotline. Hospitalizations, a record of seeking other healthcare, or serious adverse events were extracted by site personnel from the participant's medical record. Medical occurrences occurring before the receipt of study drug/placebo but after obtaining informed consent were not considered an adverse event.

Independent Data Monitoring Committee Oversight

Interim analyses were planned at intervals of approximately 300 participants contributing to a study drug group, with an anticipated maximum of 1200 participants. There was also the potential to extend accrual for a study drug if there was potential to demonstrate benefit for hospitalization/death. Due to extremely rapid enrollment, the last planned interim analysis was not conducted. The independent data monitoring committee reviewed interim data when approximately 300 and 600 participants had complete primary endpoint data, resulting in a planned primary analysis highly conservative of type 1 error. To provide additional context, the primary analysis was additionally performed with a non-informative prior and without a prior.

Handling of Missing Data

In both the primary and secondary endpoint analyses, missing data among covariates was addressed with conditional mean imputation. Approximately 3–4.5% of participants did not report activity level for the COVID clinical progression score endpoint at each time point, but the participants were known to be alive and at home. The missing activity levels was a type of interval censored outcome, as the participants were known to be either a 1 or 2 on the scale. The ordinal regression models were fit accounting for the interval censoring.

© 2024 Rothman RL et al. *JAMA Network Open*.

Proportional Hazards

The proportional hazards assumption of the primary endpoint was evaluated by generating visual diagnostics such as the log-log plot and plots of time-dependent regression coefficients for each predictor in the model, a diagnostic which indicates deviations from proportionality if the time-dependent coefficients are not constant in time.

Heterogeneity of Treatment Effect Analysis

For each characteristic, a proportional hazards regression model was constructed using the same covariates as the primary endpoint model plus additional interaction terms between treatment assignment and the characteristic of interest. To allow the possibility of non-linear trends along continuous characteristics, like age or calendar time, continuous covariates were included in the model as restricted cubic splines. The hazard ratios and 95% confidence intervals were calculated from asymptotic, model-based estimates at specific values. The continuous variables were not discretized into bins (or groups).

COVID-19 Ordinal Outcome Scale

The COVID-19 outcomes for this trial are based on the World Health Organization's Ordinal Scale for Clinical Improvement and will be collected via the online system and from the medical record. The following outcomes will be assessed as part of the COVID Clinical Progression Scale:

0. No clinical or virological evidence of infection
1. No limitation of activities
2. Limitation of activities
3. Hospitalized, no oxygen therapy
4. Hospitalized, on oxygen by mask or nasal prongs
5. Hospitalized, on non-invasive ventilation or high-flow oxygen
6. Hospitalized, on intubation and mechanical ventilation
7. Hospitalized, on ventilation + additional organ support – pressors, renal replacement therapy, extracorporeal membrane oxygenation
8. Death

© 2024 Rothman RL et al. *JAMA Network Open*.

eTable 1. Baseline symptom prevalence and severity

Variable	Montelukast (n=628)	Placebo (n=622)	Overall (n=1250)
Symptom burden on study day 1, No./total (%)			
None	21/558 (3.76)	20/553 (3.62)	41/1111 (3.69)
Mild	186/558 (33.33)	183/553 (33.09)	369/1111 (33.21)
Moderate	341/558 (61.11)	337/553 (60.94)	678/1111 (61.03)
Severe	10/558 (1.79)	13/553 (2.35)	23/1111 (2.07)
Symptoms on study day 1			
Fatigue, No./total (%)			
None	172/538 (31.97)	158/533 (29.64)	330/1071 (30.81)
Mild	139/538 (25.84)	138/533 (25.89)	277/1071 (25.86)
Moderate	207/538 (38.48)	213/533 (39.96)	420/1071 (39.22)
Severe	20/538 (3.72)	24/533 (4.50)	44/1071 (4.11)
Dyspnea, No./total (%)			
None	378/538 (70.26)	347/533 (65.10)	725/1071 (67.69)
Mild	107/538 (19.89)	137/533 (25.70)	244/1071 (22.78)
Moderate	47/538 (8.74)	41/533 (7.69)	88/1071 (8.22)
Severe	6/538 (1.12)	8/533 (1.50)	14/1071 (1.31)
Fever, No./total (%)			
None	386/538 (71.75)	371/533 (69.61)	757/1071 (70.68)
Mild	67/538 (12.45)	73/533 (13.70)	140/1071 (13.07)
Moderate	85/538 (15.80)	85/533 (15.95)	170/1071 (15.87)
Severe	0/538 (0.00)	4/533 (0.75)	4/1071 (0.37)
Cough, No./total (%)			
None	167/537 (31.10)	138/533 (25.89)	305/1070 (28.50)
Mild	146/537 (27.19)	141/533 (26.45)	287/1070 (26.82)
Moderate	206/537 (38.36)	233/533 (43.71)	439/1070 (41.03)
Severe	18/537 (3.35)	21/533 (3.94)	39/1070 (3.64)
Nausea, No./total (%)			
None	458/537 (85.29)	451/533 (84.62)	909/1070 (84.95)
Mild	59/537 (10.99)	60/533 (11.26)	119/1070 (11.12)
Moderate	19/537 (3.54)	19/533 (3.56)	38/1070 (3.55)
Severe	1/537 (0.19)	3/533 (0.56)	4/1070 (0.37)
Vomiting, No./total (%)			
None	501/537 (93.30)	500/533 (93.81)	1001/1070 (93.55)
Mild	29/537 (5.40)	26/533 (4.88)	55/1070 (5.14)
Moderate	6/537 (1.12)	4/533 (0.75)	10/1070 (0.93)
Severe	1/537 (0.19)	3/533 (0.56)	4/1070 (0.37)

© 2024 Rothman RL et al. *JAMA Network Open*.

Variable	Montelukast (n=628)	Placebo (n=622)	Overall (n=1250)
Diarrhea, No./total (%)			
None	454/537 (84.54)	429/533 (80.49)	883/1070 (82.52)
Mild	60/537 (11.17)	70/533 (13.13)	130/1070 (12.15)
Moderate	19/537 (3.54)	30/533 (5.63)	49/1070 (4.58)
Severe	4/537 (0.74)	4/533 (0.75)	8/1070 (0.75)
Body aches, No./total (%)			
None	189/537 (35.20)	188/533 (35.27)	377/1070 (35.23)
Mild	132/537 (24.58)	132/533 (24.77)	264/1070 (24.67)
Moderate	207/537 (38.55)	188/533 (35.27)	395/1070 (36.92)
Severe	9/537 (1.68)	25/533 (4.69)	34/1070 (3.18)
Sore throat, No./total (%)			
None	264/537 (49.16)	269/533 (50.47)	533/1070 (49.81)
Mild	122/537 (22.72)	127/533 (23.83)	249/1070 (23.27)
Moderate	141/537 (26.26)	119/533 (22.33)	260/1070 (24.30)
Severe	10/537 (1.86)	18/533 (3.38)	28/1070 (2.62)
Headache, No./total (%)			
None	245/537 (45.62)	260/533 (48.78)	505/1070 (47.20)
Mild	126/537 (23.46)	116/533 (21.76)	242/1070 (22.62)
Moderate	157/537 (29.24)	136/533 (25.52)	293/1070 (27.38)
Severe	9/537 (1.68)	21/533 (3.94)	30/1070 (2.80)
Chills, No./total (%)			
None	371/537 (69.09)	363/532 (68.23)	734/1069 (68.66)
Mild	80/537 (14.90)	74/532 (13.91)	154/1069 (14.41)
Moderate	84/537 (15.64)	88/532 (16.54)	172/1069 (16.09)
Severe	2/537 (0.37)	7/532 (1.32)	9/1069 (0.84)
Nasal symptoms, No./total (%)			
None	244/537 (45.44)	230/533 (43.15)	474/1070 (44.30)
Mild	148/537 (27.56)	144/533 (27.02)	292/1070 (27.29)
Moderate	131/537 (24.39)	139/533 (26.08)	270/1070 (25.23)
Severe	14/537 (2.61)	20/533 (3.75)	34/1070 (3.18)
New loss of sense of taste or smell, No./total (%)			
None	389/537 (72.44)	367/533 (68.86)	756/1070 (70.65)
Mild	68/537 (12.66)	76/533 (14.26)	144/1070 (13.46)
Moderate	57/537 (10.61)	65/533 (12.20)	122/1070 (11.40)
Severe	23/537 (4.28)	25/533 (4.69)	48/1070 (4.49)

© 2024 Rothman RL et al. *JAMA Network Open*.

eTable 2. Serious adverse events

Variable	Measure	Montelukast	Placebo	Overall
Serious adverse events	N			
Pneumonia		1	1	2
Acute appendicitis		0	1	1
Cellulitis of legs		1	0	1
Torsion of ovary		1	0	1

© 2024 Rothman RL et al. *JAMA Network Open*.

eFigure 1. Time from symptom onset to receipt of drug

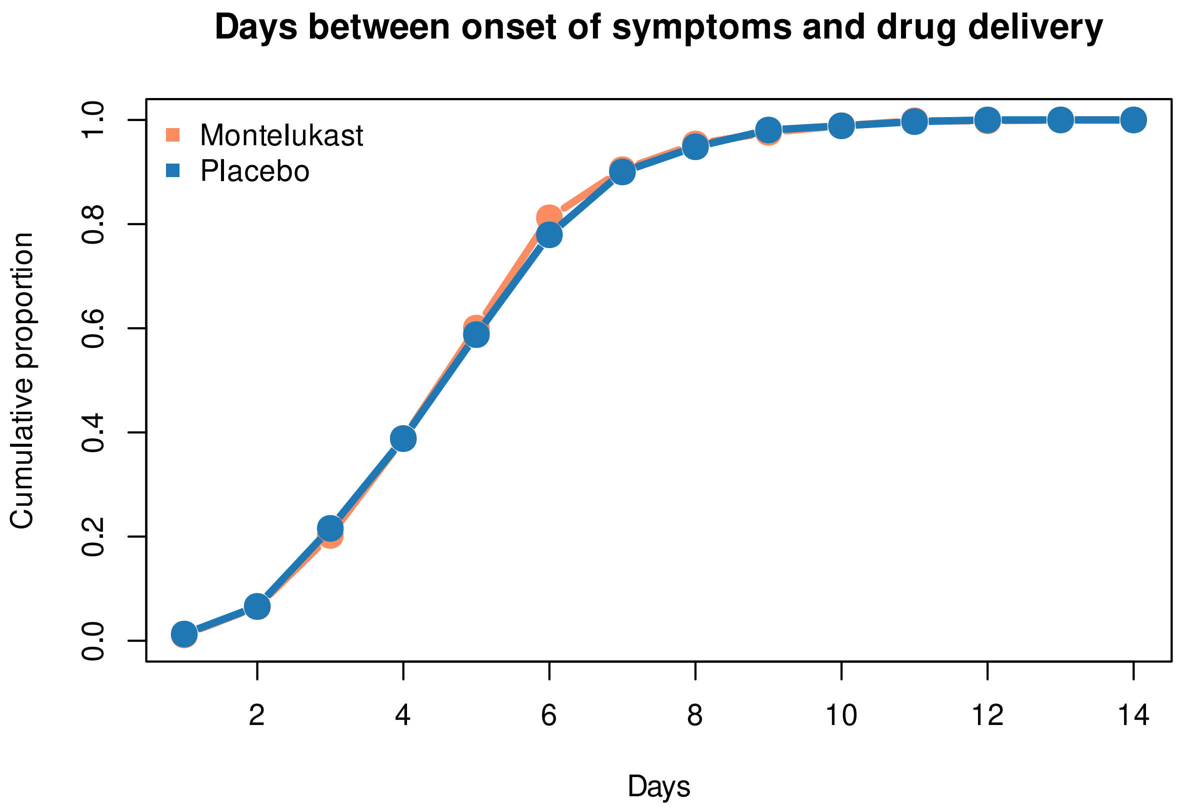

Cumulative proportion by day														
	1	2	3	4	5	6	7	8	9	10	11	12	13	14
Montelukast	0.01	0.07	0.20	0.39	0.60	0.81	0.9	0.95	0.98	0.99	1	1	1	1
Placebo	0.01	0.07	0.22	0.39	0.59	0.78	0.9	0.95	0.98	0.99	1	1	1	1

© 2024 Rothman RL et al. *JAMA Network Open*.

eFigure 2. Sensitivity analysis of hazard ratio for time to sustained recovery

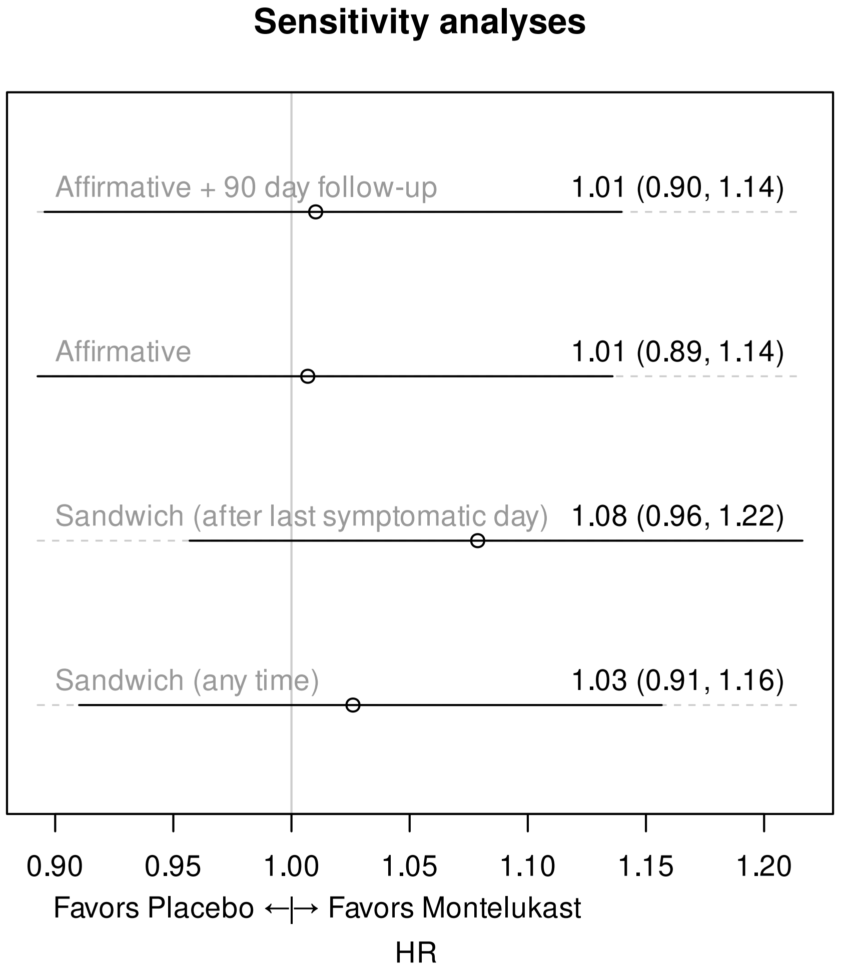

Sustained recovery is defined as three consecutive symptom-free days. This figure illustrates the hazard ratio of the treatment effect estimate for different strategies of handling missing patient responses. The primary "Affirmative + 90 day follow-up" definition assumes that participants are symptomatic on the days without a response. The strategy does not censor recovery times earlier than day 28 if the day 90 survey is provided. The "Affirmative" assumes the same about days without a response. It differs for the primary definition in that follow-up is only based on responses during the first 28 days. A participant that fails to respond to surveys after day 14 is censored at day 14. The second and third alternatives are imputation strategies, treating missing responses as symptom-free days if surrounded by symptom-free days. For instance, if a participant lacked a day two response but reported no symptoms on days one and three, the "Sandwich" definition marks the missing day as "no symptoms." The "Sandwich after last symptomatic day" definition only applies this rule after the last reported symptomatic day.

The hazard ratios in the figure are from the covariate-adjusted, proportional hazards regression model without prior. Notably, irrespective of the sustained recovery definition, the treatment effect remains largely consistent.

© 2024 Rothman RL et al. *JAMA Network Open*.

eFigure 3. All-cause hospitalization or death for montelukast versus placebo

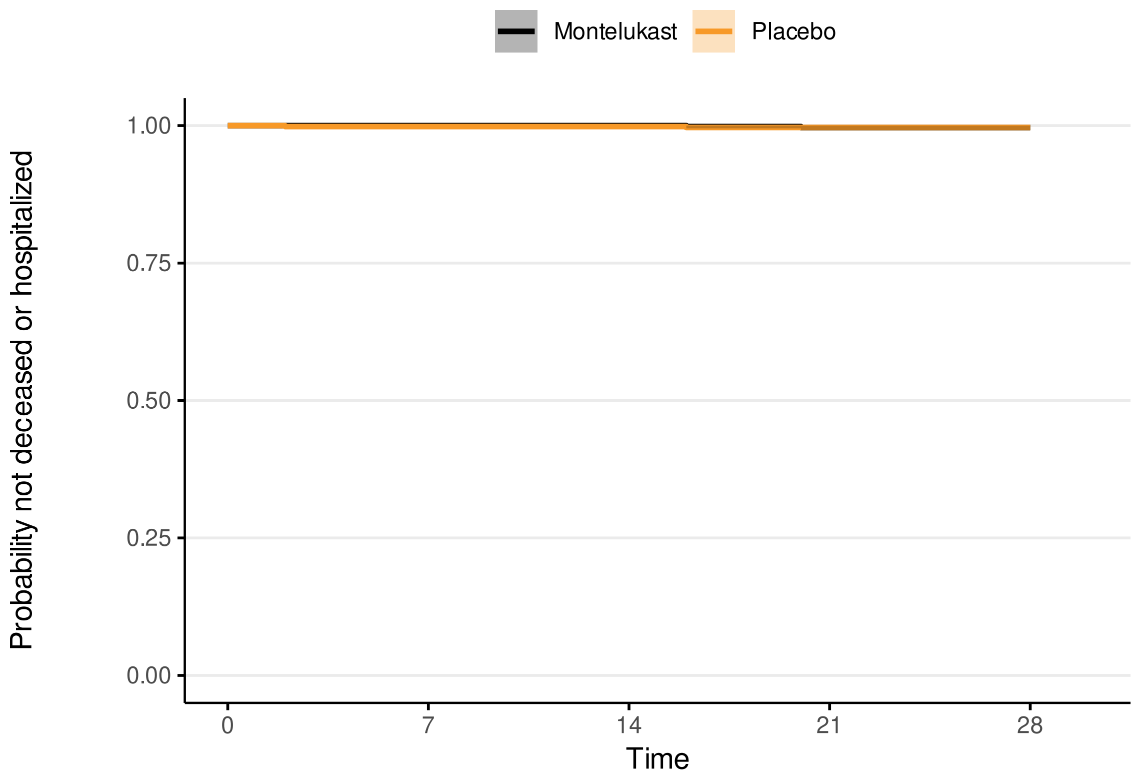

No. at risk

	0	7	14	21	28
Montelukast	628	606	594	589	583
Placebo	622	601	595	585	582

No deaths were reported and 2 hospitalizations occurred in each group.

© 2024 Rothman RL et al. *JAMA Network Open*.

eFigure 4. All-cause hospitalization, urgent care, emergency department visit, or death for montelukast versus placebo

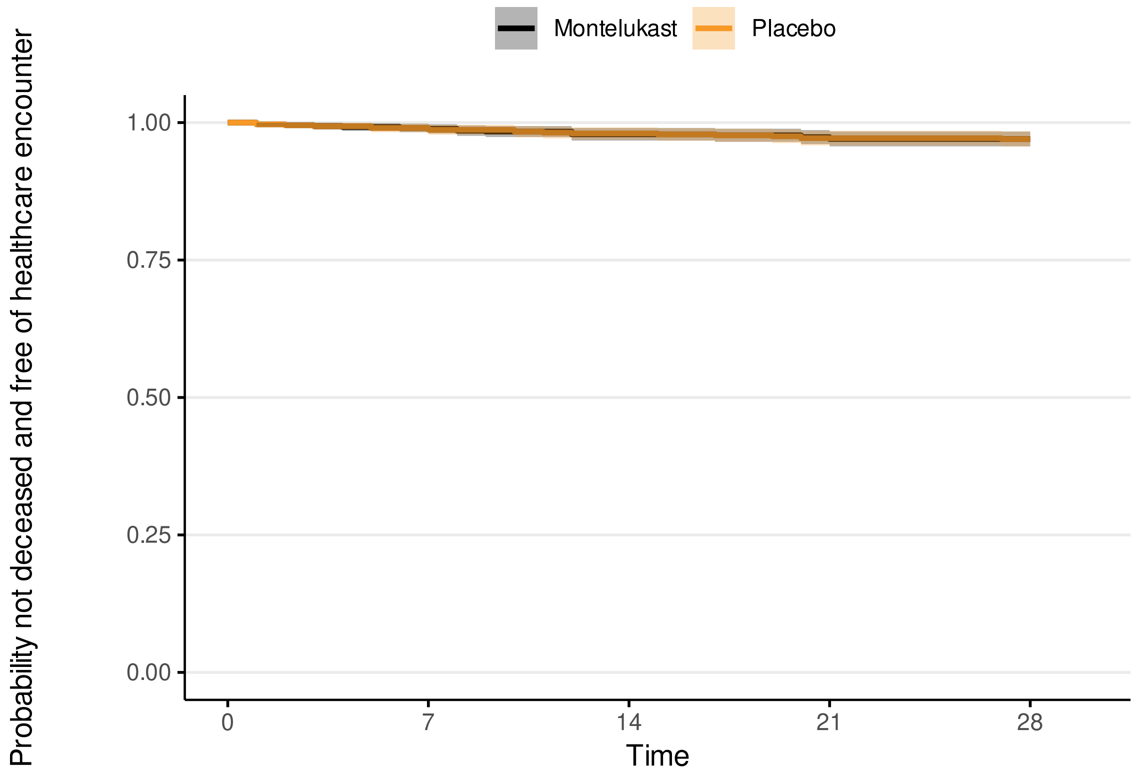

No. at risk

Montelukast	628	601	582	576	568
Placebo	622	596	584	571	567

Overall, 36 participants reported healthcare utilization events (a priori defined as death, hospitalization, emergency department/urgent care visit); 18 in the montelukast group compared with 18 in the placebo group (HR 1.01, 95% CrI 0.45–1.84; P(efficacy)=0.48).

eFigure 5. Participants' COVID clinical progression scale at day 7, 14, and 28

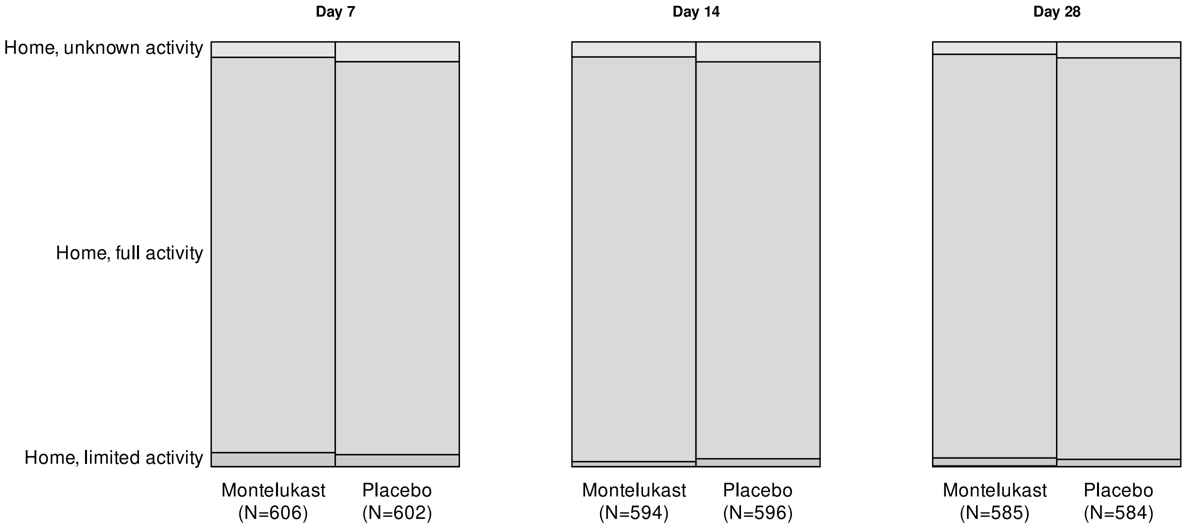

© 2024 Rothman RL et al. *JAMA Network Open*.

eFigure 6. Kaplan-Meier curves of sustained recovery by onset of symptoms and symptom severity

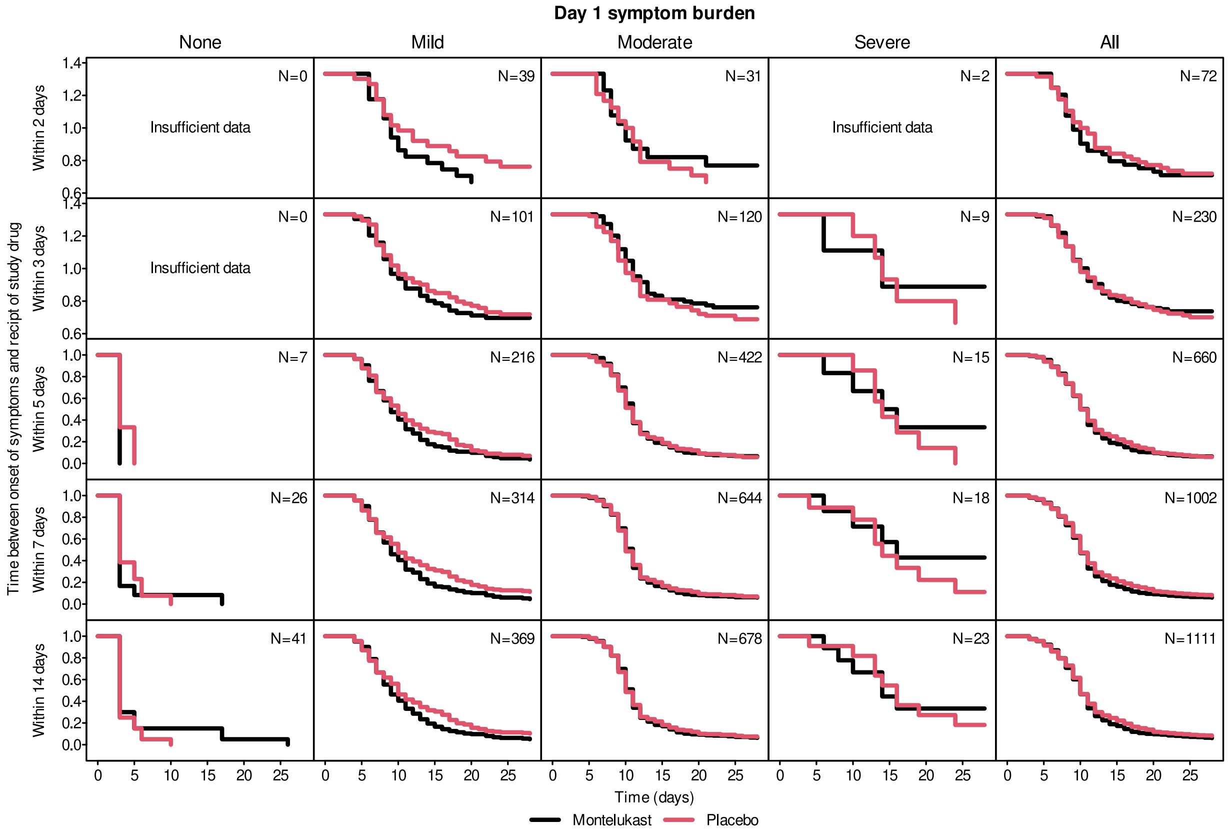

© 2024 Rothman RL et al. *JAMA Network Open*.

eFigure 7. Heterogeneity of treatment effect between montelukast and placebo for time to recovery

Characteristic	Value	Montelukast N	Placebo N	HR (95% CI)	HTE p-value
Vaccination status	Vaccinated	349	355	0.99 (0.83, 1.17)	0.679
	Not vaccinated	279	267	1.04 (0.87, 1.23)	
Sex	Male	240	257	1.04 (0.87, 1.26)	0.654
	Female	388	365	0.99 (0.84, 1.15)	
Calendar time	2023-02-15			0.94 (0.76, 1.16)	0.274
	2023-03-15			0.99 (0.92, 1.07)	
	2023-04-15			1.00 (0.99, 1.00)	
	2023-05-15			0.94 (0.86, 1.03)	
	2023-06-15			0.86 (0.68, 1.08)	
Symptom onset, days	3			1.01 (0.83, 1.24)	0.021
	5			1.13 (0.97, 1.32)	
	7			0.91 (0.77, 1.09)	
	9			0.64 (0.45, 0.91)	
Age, years	40			1.01 (0.85, 1.20)	0.136
	50			1.07 (0.91, 1.27)	
	60			1.11 (0.95, 1.31)	
	70			1.14 (0.94, 1.39)	
Body mass index, kg/m^2	20			0.87 (0.57, 1.35)	0.739
	25			0.96 (0.79, 1.16)	
	30			1.03 (0.88, 1.20)	
	35			1.06 (0.90, 1.25)	
	40			1.07 (0.83, 1.39)	
	45			1.08 (0.72, 1.63)	
Symptoms on study day 1	None	21	20	0.14 (0.07, 0.26)	< 0.001
	Mild	186	183	1.19 (0.95, 1.48)	
	Moderate	341	337	1.03 (0.88, 1.20)	
	Severe	10	13	0.73 (0.26, 2.05)	
Overall mITT population		628	622	1.01 (0.90, 1.14)	

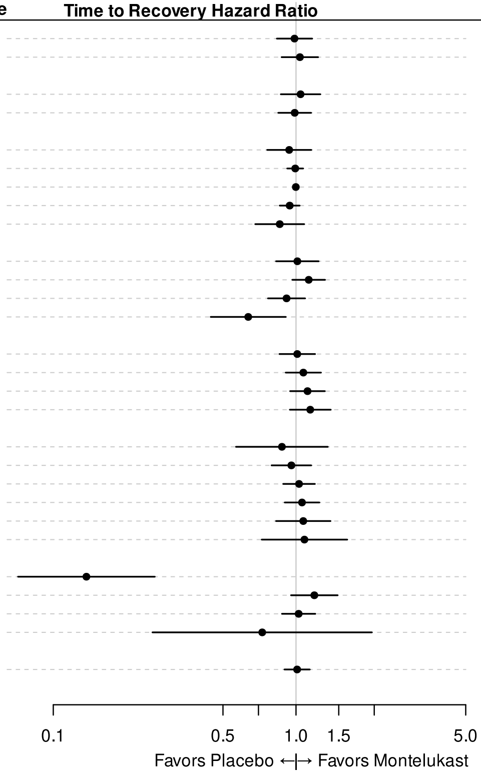

This figure presents the analyses exploring heterogeneity of treatment effect derived from the primary endpoint—time to sustained recovery. These exploratory analyses, outlined in the statistical analysis plan, did not undergo multiplicity adjustment. A hazard ratio larger than 1 signifies an accelerated recovery. Study day 1 corresponds to the participant's drug delivery day.

For each characteristic, a proportional hazards regression model was constructed using the same covariates as the primary endpoint model plus additional interaction terms between treatment assignment and the characteristic of interest. For example, the interaction of vaccination status and treatment assignment was added to the primary endpoint regression model to calculate a treatment effect for the vaccinated and unvaccinated subgroups.

To allow the possibility of non-linear trends along continuous characteristics, like age or calendar time, the additional terms were interactions between treatment assignment and restricted cubic splines. Because the primary endpoint model did not include body mass index (BMI), the restricted cubic spline terms for BMI were also added to the model (sometimes call main effects) in addition to the interaction terms. Because the primary endpoint model only included a single linear term for symptom onset, the nonlinear terms of the restricted cubic spline were also added to the model in addition to the interaction terms. The hazard ratios and 95% confidence intervals were calculated from asymptotic, model-based contrasts.

The hazard ratio for the full study population was generated from the primary endpoint model without prior adjustments. The estimates shown in the HTE plot are estimates calculated from the smooth, modeled relationship. For the continuous characteristics, there are no discrete categories or ranges or bins over which to tabulate the number of participants.

© 2024 Rothman RL et al. *JAMA Network Open*.

eFigure 8. Posterior distribution of treatment effect adjusted for site

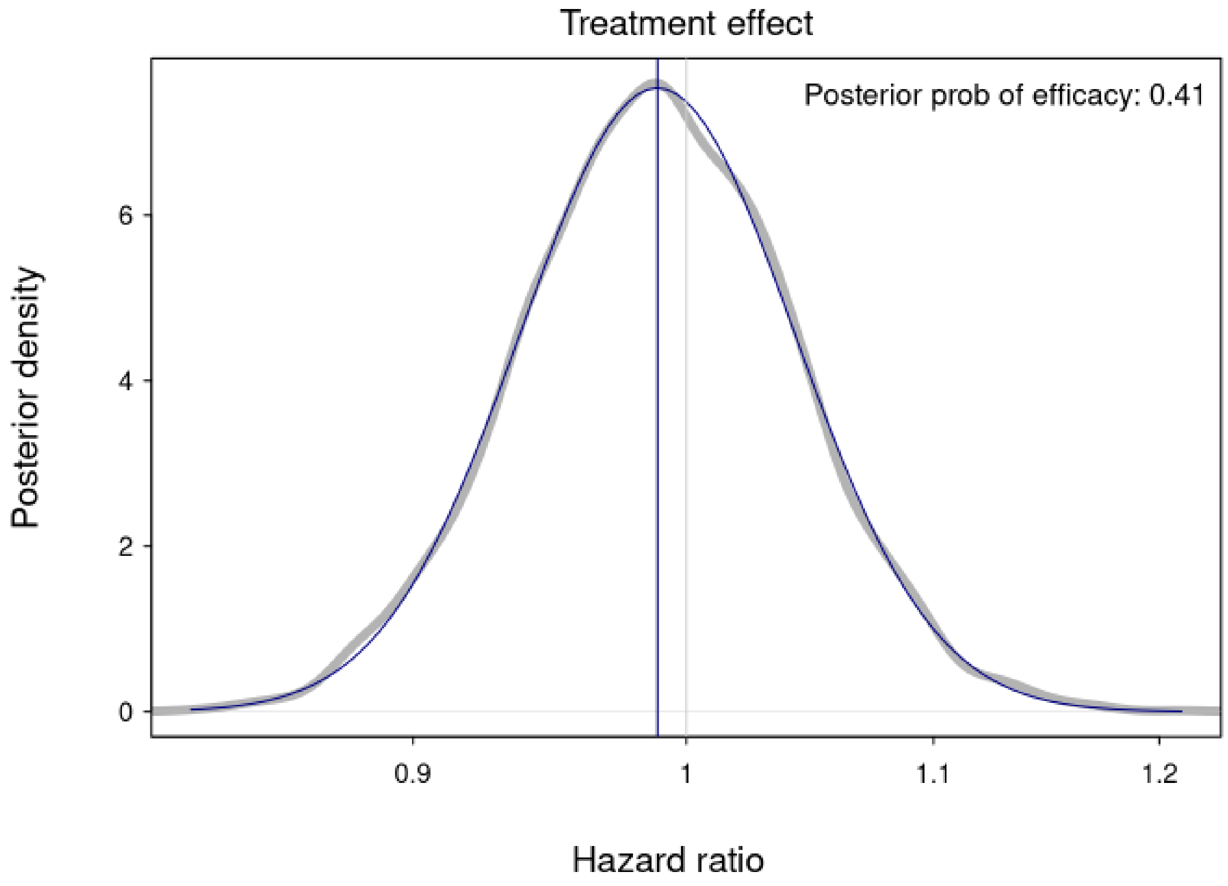

As a post-hoc, sensitivity analysis, the primary endpoint model was expanded to include indicators for individual sites. The resulting posterior distribution for treatment effect derived from the model is shown above. The thicker grey line represents the kernel density estimate constructed from the posterior draws. The thinner black line represents the parametric density estimate constructed from the same draws.

© 2024 Rothman RL et al. *JAMA Network Open*.

eFigure 9. Heterogeneity of treatment effect by site

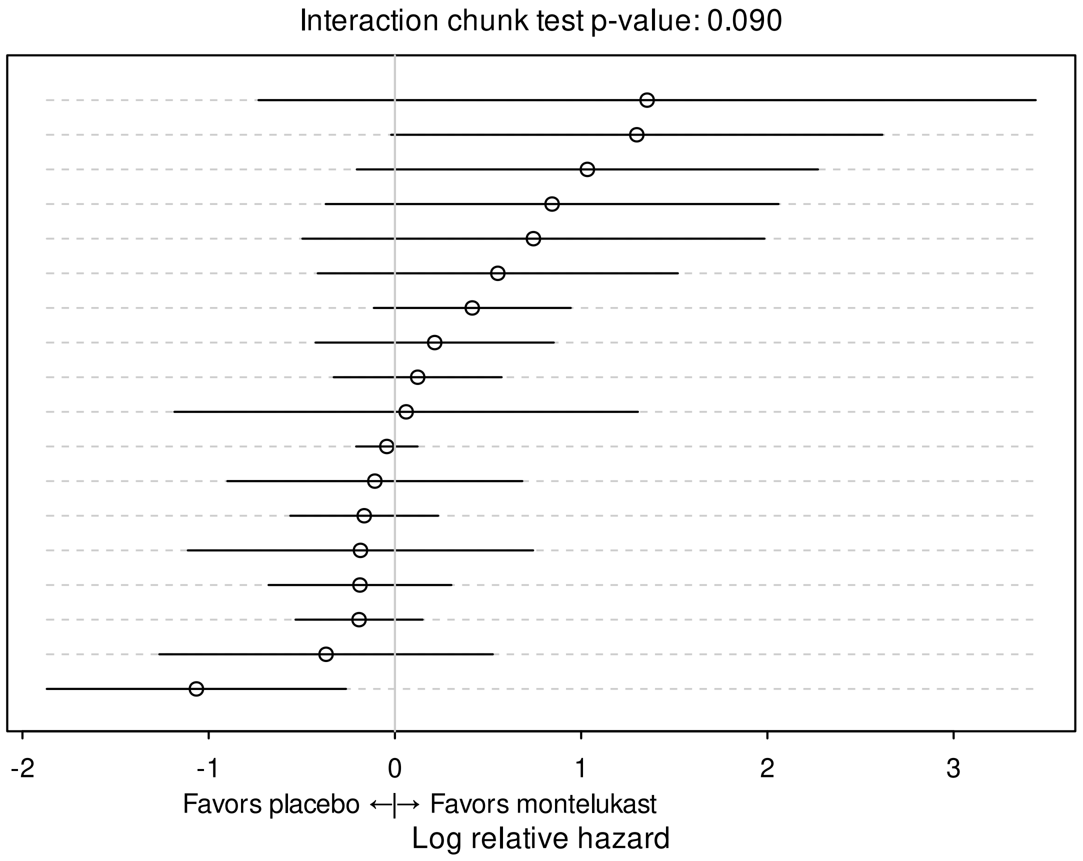

As a post-hoc, sensitivity analysis, heterogeneity of treatment effect by site was investigated, implementing the same methods as those used for the planned analysis in eFigure 7. Sites with fewer than 11 participants were combined into a single group. This figure shows the site-specific treatment effects and 95% confidence intervals.

© 2024 Rothman RL et al. *JAMA Network Open*.

eFigure 10. Posterior densities of primary end point model

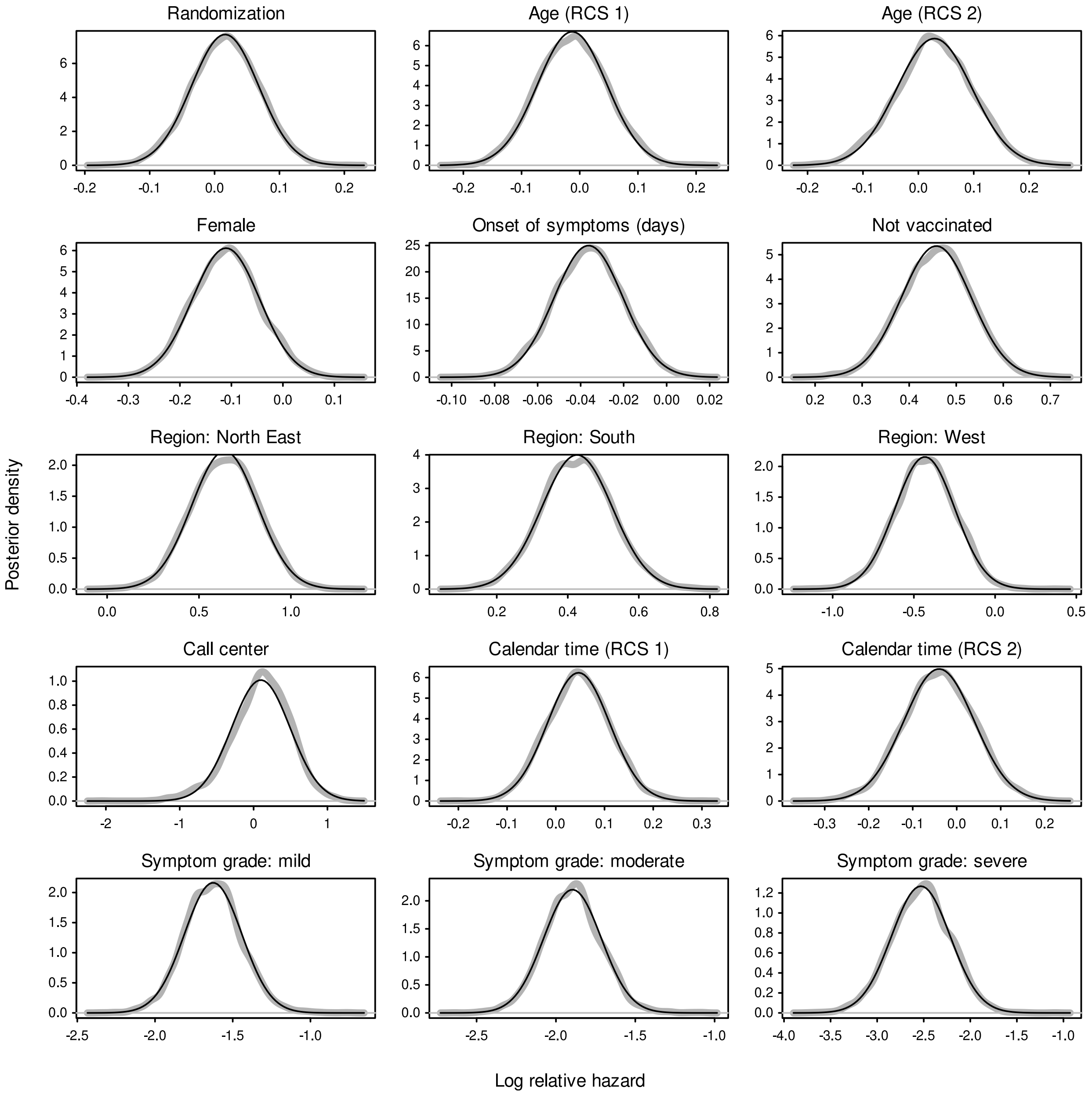

This figure provides the posterior densities of each regression coefficient in the primary endpoint model. The x-axis is the log relative hazard; the x-axis is density. The thick, grey lines are the kernel density estimates; thin, black lines represent parametric normal density estimates of the same.

© 2024 Rothman RL et al. *JAMA Network Open*.
