# Supplementary material for: Time to Sustained Recovery Among Outpatients With COVID-19 Receiving Montelukast vs Placebo: The ACTIV-6 Randomized Clinical Trial
Source: JAMA Netw Open. 2024 Oct 18;7(10):e2439332. doi: 10.1001/jamanetworkopen.2024.39332 (PMC11581631; doi:10.1001/jamanetworkopen.2024.39332)
Supplement: Supplement 3. — Accelerating COVID-19 Therapeutic Interventions and Vaccines (ACTIV)–6 Study Group and Investigators [file jamanetwopen-e2439332-s003.pdf]

Supplemental Online Content: Nonauthor Collaborators
*First name, last name, and suffix (if applicable) are required and will appear in PubMed.

*Group Name(s): Accelerating COVID-19 Therapeutic Interventions and Vaccines (ACTIV)–6 Study Group and Investigators							
***First Name and Middle Initial(s)**	***Last Name**	***Suffix (eg, Jr, III)**	Academic Degrees	Institution	Location (city, state/province, country)	Role or Contribution, eg, chair, principal investigator	Group (if more than 1 Group listed in the byline) and/or Subgroup (eg, Steering Committee)
George	Hanna						
Ryan	Fraser						
Mark	Ward						
Jennifer	Gamboa Jackson						
M. Patricia	McAdams						
Julia	Vail						
Kayla	Korzekwinski						
Martina	Oyelakin						
Julie	Chopp						
Desmon	Randle						
Samantha	Dockery						
Rodney	Adkins						
Matthew	Crow						
Erin	Nowell						
Kadie	Wells						
Alicia	Herbert						
Allegra	Stone						
Heather	Heavlin						
Linley	Brown						
Tina	Harding						
Amanda	Harrington						
Meaghan	Beauchaine						
Kelly	Lindblom						
Andrea	Burns						
David	Aamodt						
Jess	Collins						
Sheri	Dixon						
Yue	Gao						
John	Graves						

Supplemental Online Content: Nonauthor Collaborators
*First name, last name, and suffix (if applicable) are required and will appear in PubMed.

***First Name and Middle Initial(s)**	***Last Name**	***Suffix (eg, Jr, III)**	Academic Degrees	Institution	Location (city, state/province, country)	Role or Contribution, eg, chair, principal investigator	Group (if more than 1 Group listed in the byline) and/or Subgroup (eg, Steering Committee)
James	Grindstaff						
Frank	Harrell						
Jessica	Lai						
Vicky	Liao						
Itzel	Lopez						
Elizabeth	Manis						
Kalley	Mankowski						
Jessica	Marlin						
Alyssa	Merkel						
Sam	Nwosu						
Savannah	Obregon						
Dirk	Orozco						
Nelson	Prato						
Max	Rhode						
Jana	Shirey-Rice						
Krista	Vermillion						
Jacob	Smith						
Hsi-nien	Tan						
Meghan	Vance						
Maria	Weir						
Ray	Bianchi						
Jen	Premas						
Madhu	Gupta						
Greg	Karawan						
Santia	Lima						
Carey	Ziomek						
Joseph	Arena						
Sonaly	DeAlmeida						
Anuj	Malik						
Jane	Bryce						
Sarah	Swint						

Supplemental Online Content: Nonauthor Collaborators
*First name, last name, and suffix (if applicable) are required and will appear in PubMed.

***First Name and Middle Initial(s)**	***Last Name**	***Suffix (eg, Jr, III)**	Academic Degrees	Institution	Location (city, state/province, country)	Role or Contribution, eg, chair, principal investigator	Group (if more than 1 Group listed in the byline) and/or Subgroup (eg, Steering Committee)
Soroush	Ramin						
Jaya	Nataraj						
Julien	Deider						
Ricardo	Cruz						
Ana Maria	Ramirez						
Lori	Henault						
Joseph	Marcus						
Alexis	Southwell						
Genice	Jacques						
Cedar	Sexton						
Brian	Tiffany						
Charlotte	Tanner						
Allegra	Sahelian						
Constance	George-Adebayo						
Adeolu	Adebayo						
Jose	Zapatero						
Julie	Clement						
Theresa	Ronan						
Ashley	Woods						
Christopher	Gallegos						
Tamara	Flys						
Olivia	Sloan						
Anthony	Olofintuyi						
Joshua	Samraj						
Alma	Vasbinder						
Amaya	Averett						
Alex	Slandzicki						
Jessica	Wallen						
Claudia	Vogel						
Sebastian	Munoz						
David	Kavtaradze						

Supplemental Online Content: Nonauthor Collaborators
*First name, last name, and suffix (if applicable) are required and will appear in PubMed.

***First Name and Middle Initial(s)**	***Last Name**	***Suffix (eg, Jr, III)**	Academic Degrees	Institution	Location (city, state/province, country)	Role or Contribution, eg, chair, principal investigator	Group (if more than 1 Group listed in the byline) and/or Subgroup (eg, Steering Committee)
Casandra	Watson						
David	Singleton						
Marcus	Sevier						
Maria	Rivon						
Arnold	Del Pilar						
Amber	Spangler						
Sohail	Rao						
Luis	Cantu						
Arvind	Krishna						
Heidi	Daugherty						
Brandi	Kerr						
Kathy	Evans						
Robert	Spees						
Mailyn	Marta						
Rowena	Dolor						
Lorraine	Vergara						
Jackie	Jordan						
Valencia	Burruss						
Terri	Hurst						
Igho	Ofotokun						
Cecilia	Zhang						
Jessica	Traenkner						
Mary M.	Atha						
Rajesh	Prabhu						
Krystal	Klicka						
Amber	Lightfeather						
Vickie	James						
Marcella	Rogers						
Chukwuemeka	Oragwu						
Ngozi	Oguego						
Rajesh	Pillai						

Supplemental Online Content: Nonauthor Collaborators
*First name, last name, and suffix (if applicable) are required and will appear in PubMed.

***First Name and Middle Initial(s)**	***Last Name**	***Suffix (eg, Jr, III)**	Academic Degrees	Institution	Location (city, state/province, country)	Role or Contribution, eg, chair, principal investigator	Group (if more than 1 Group listed in the byline) and/or Subgroup (eg, Steering Committee)
Ahab	Gabriel						
Emad	Ghaly						
Marian	Michal						
Michelle	Vasquez						
Angela	Mamon						
Michelle	Sheets						
Gammal	Hassanien						
Samah	Ismail						
Yehia	Samir						
Andrew	Meltzer						
Soroush	Shahamatdar						
Ryan S.	Heidish						
Aditya	Loganathan						
Scott	Brehaut						
Angelina	Roche						
Manisha	Mehta						
Nicole	Koppinger						
Jose	Baez						
Ivone	Pagan						
Dallal	Abdelsayed						
Mina	Aziz						
Philip	Robinson						
Grace	Lozinski						
Julie	Nguyen						
Alvin	Griffin						
Michael	Morris						
Nicole	Love						
Bonnie	Mattox						
Raykel	Martin						
Victoria	Pardue						
Teddy	Rowland						

Supplemental Online Content: Nonauthor Collaborators
*First name, last name, and suffix (if applicable) are required and will appear in PubMed.

***First Name and Middle Initial(s)**	***Last Name**	***Suffix (eg, Jr, III)**	Academic Degrees	Institution	Location (city, state/province, country)	Role or Contribution, eg, chair, principal investigator	Group (if more than 1 Group listed in the byline) and/or Subgroup (eg, Steering Committee)
Juan	Ruiz-Unger						
Lionel	Reyes						
Yadira	Zamora						
Navila	Bacallao						
John	Cienki						
Jonathan	Cohen						
Ying	Yuan						
Jenny	Li						
Jeremy	Szeto						
Lauren	Stelmash						
Sara	Mekhael						
Ledular	Morales Castillo						
Anya	Gutierrez						
Sabrina	Prieto						
Arch	Amon						
Andrew	Barbera						
Andrew	Bugajski						
Walter	Willis						
Kellcee	Jacklin						
Deryl	Lamb						
Amron	Harper						
Elmer	Stout						
Merischia	Griffin						
Nancy	Pyram-Bernard						
Arlen	Quintero						
Nina	Clark						
Mary	Barsanti-Sekhar						
Christina	Carbrera-Mendez						
Mary Rose	Evans						
Eftim	Adhami						
Giovanni	Carillo						

Supplemental Online Content: Nonauthor Collaborators
*First name, last name, and suffix (if applicable) are required and will appear in PubMed.

***First Name and Middle Initial(s)**	***Last Name**	***Suffix (eg, Jr, III)**	Academic Degrees	Institution	Location (city, state/province, country)	Role or Contribution, eg, chair, principal investigator	Group (if more than 1 Group listed in the byline) and/or Subgroup (eg, Steering Committee)
Josette	Maria						
Diksha	Paudel						
Oksana	Raymond						
Jeffrey	Summers						
Tammy	Turner						
Leslie	Lenert						
Ebony	Panaccione						
Elizabeth	Szwast						
Amy	Reynolds						
Ahsan	Abdulghani						
Pravin	Vasoya						
Conrad	Miller						
Hawa	Wiley						
Austin	Chan						
Saadia	Khizer						
Oluwadamilola	Adeyemi						
Wei Ning	Chi						
July	Chen						
Melissa	Morton-Jost						
Julie	Castex						
Ali	Quirch						
Hrishikesh	Belani						
Rosario	Machicado						
Bjorn	Bjornsson						
Jacqueline	Olivo						
Maria	Maldonado						
Anthony	Vecchiarelli						
Diana	Gaytan-Alvarez						
Vijaya	Cherukuri						
Radica	Alicic						
Allison A.	Lambert						

Supplemental Online Content: Nonauthor Collaborators
*First name, last name, and suffix (if applicable) are required and will appear in PubMed.

***First Name and Middle Initial(s)**	***Last Name**	***Suffix (eg, Jr, III)**	Academic Degrees	Institution	Location (city, state/province, country)	Role or Contribution, eg, chair, principal investigator	Group (if more than 1 Group listed in the byline) and/or Subgroup (eg, Steering Committee)
Carissa	Urbat						
Joni	Baxter						
Ann	Cooper						
Dawn	Linn						
Laura	Fisher						
Vijay	Patel						
Roshan	Talati						
Priti	Patel						
Leonard	Ellison						
Angee	Roman						
Jeffrey	Harrison						
James	Moy						
Dina	Naquiallah						
Binod	Shah						
Orlando	Quintero						
Jake	Scott						
Yasmin	Jazayeri						
Andrew	O'Donnell						
Divya	Pathak						
Anita	Gupta						
N.	Chandrasekar						
Clifford	Curtis						
Briana	White						
Martha	Dockery						
Tabitha	Fortt						
Anisa	Fortt						
Ingrid	Jones-Ince						
Alix	McKee						
Jason	Wilson						
Jackie	Marcelin						
Brenda	Farlow						

Supplemental Online Content: Nonauthor Collaborators
*First name, last name, and suffix (if applicable) are required and will appear in PubMed.

***First Name and Middle Initial(s)**	***Last Name**	***Suffix (eg, Jr, III)**	Academic Degrees	Institution	Location (city, state/province, country)	Role or Contribution, eg, chair, principal investigator	Group (if more than 1 Group listed in the byline) and/or Subgroup (eg, Steering Committee)
Casey	Grady						
Randall	Richwine						
Penny	Pazier						
Edward	Michelson						
Susan	Watts						
Diluma	Kariyawasam						
Leann	Rodriguez						
Ismarys	Manresa						
Angel A.	Achong						
Mari C.	Garcia						
Sangeeta	Khetpal						
Faith	Posey						
Arvind	Mahadevan						
Martin	Gnoni						
Carla	Van de Weerd						
Jeffrey	Lowenkron						
Erica	Sappington						
Mitchell	Roberts						
Jennifer	Wang						
Melissa	Adams						
Xinyi	Ding						
Mary	Co						
Mark	D'Andrea						
Stephen	Lim						
Wayne	Swink						
Emily	Bozant						
Madeline	Young						
Michael	Wilson						
Carly	Eastin						
Allyson	Cheathem						
Ahad	Nadeem						

Supplemental Online Content: Nonauthor Collaborators
*First name, last name, and suffix (if applicable) are required and will appear in PubMed.

***First Name and Middle Initial(s)**	***Last Name**	***Suffix (eg, Jr, III)**	Academic Degrees	Institution	Location (city, state/province, country)	Role or Contribution, eg, chair, principal investigator	Group (if more than 1 Group listed in the byline) and/or Subgroup (eg, Steering Committee)
Crystal	Walters						
Margaret	Powers-Fletcher						
Douglas	Brown						
Delia	Miller						
Sylvere	Mukunzi						
Brittney	Manning						
Melissa	Terry-White						
Maria Christina	Crizaldo						
Carmen	Isache						
Jennifer	Bowman						
Angelique	Callaghan-Brown						
Debra	Martin						
Ashley	Ast						
Brent	Duran						
Ashlie	Cornejo						
Allie	Archer						
Maria	Almanzar						
Vanessa	Motel						
Matt	Pullen						
Blake	Anderson						
Neeta	Bhat						
Daniela	Parra						
Paula	Campora						
Matthew	Robinson						
Michelle	Seithel						
Liz	Kendrick						
Dyann	Helming						
Kelly	Pollock						
Akira	Sekikawa						
Emily	Klawson						
Jonathan	Arnold						

*First name, last name, and suffix (if applicable) are required and will appear in PubMed.

*First Name and Middle Initial(s)	*Last Name	*Suffix (eg, Jr, III)	Academic Degrees	Institution	Location (city, state/province, country)	Role or Contribution, eg, chair, principal investigator	Group (if more than 1 Group listed in the byline) and/or Subgroup (eg, Steering Committee)
Nathan	Weiland						
Luis	Ostrosky-Zeichner						
Bela	Patel						
Virginia	Umana						
Laura	Nielsen						
Carolyn Z.	Grimes						
Thomas F.	Patterson						
Robin	Tragus						
Bridgette T.	Soileau						
Timothy	Heath						
Erik	Hinjosa						
Cesar	Gutierrez						
Patrick E.H.	Jackson						
Caroline	Hallowell						
Heather M.	Haughey						
Bhavna	Vaidya-Tank						
Cameron	Gould						
Parul	Goyal						
Sue	Sommers						
Haley	Pangburn						
Carly	Jones						
Lori	Michalowski						
Brittany	Wortham						
Rica	Abbott						
Unwana	Umana						
Candace	Alleyne						
Britta	Witting						
Eddie	Armas						
Ramon O.	Perez Landaburo						
Michelle	De La Cruz						
Martha	Ballmajo						

Supplemental Online Content: Nonauthor Collaborators
*First name, last name, and suffix (if applicable) are required and will appear in PubMed.

***First Name and Middle Initial(s)**	***Last Name**	***Suffix (eg, Jr, III)**	Academic Degrees	Institution	Location (city, state/province, country)	Role or Contribution, eg, chair, principal investigator	Group (if more than 1 Group listed in the byline) and/or Subgroup (eg, Steering Committee)
Jorge	Alvarez						
