# Supplementary material for: Time to Sustained Recovery Among Outpatients With COVID-19 Receiving Montelukast vs Placebo: The ACTIV-6 Randomized Clinical Trial
Source: JAMA Netw Open. 2024 Oct 18;7(10):e2439332. doi: 10.1001/jamanetworkopen.2024.39332 (PMC11581631; doi:10.1001/jamanetworkopen.2024.39332)
Supplement: Supplement 4. — Data Sharing Statement [file jamanetwopen-e2439332-s004.pdf]

Data Sharing Statement

Rothman. Time to Sustained Recovery Among Outpatients With COVID-19 Receiving Montelukast vs Placebo. *JAMA Netw Open*. Published October 18, 2024. doi:10.1001/jamanetworkopen.2024.39332

Data
Additional Information: ClinicalTrials.gov (NCT04885530).
Data available: Yes
Data types: Deidentified participant data
How to access data: ACTIV-6 is a platform trial using shared placebos. On completion of the platform trial, when there is no risk of unblinding across study arms, the data will be made publicly available by depositing it in an approved data repository such as NHLBI's BioData Catalyst.
When available: beginning date: 07-31-2024

Supporting Documents
Document types: Other (please specify)
Additional Information: Not applicable
How to access documents: Not applicable
When available: beginning date: 07-31-2024

Additional Information
Who can access the data: ACTIV-6 is a platform trial using shared placebos. On completion of the platform trial, when there is no risk of unblinding across study arms, the data will be made publicly available by depositing it in an approved data repository such as NHLBI's BioData Catalyst.
Types of analyses: ACTIV-6 is a platform trial using shared placebos. On completion of the platform trial, when there is no risk of unblinding across study arms, the data will be made publicly available by depositing it in an approved data repository such as NHLBI's BioData Catalyst.
Mechanisms of data availability: ACTIV-6 is a platform trial using shared placebos. On completion of the platform trial, when there is no risk of unblinding across study arms, the data will be made publicly available by depositing it in an approved data repository such as NHLBI's BioData Catalyst.
